# Modulation of H4K16Ac levels reduces pro-fibrotic gene expression and mitigates lung fibrosis in aged mice

**DOI:** 10.7150/thno.62760

**Published:** 2022-01-01

**Authors:** Xiangyu Zhang, Hui Liu, Jennifer Q Zhou, Stefanie Krick, Jarrod W Barnes, Victor J Thannickal, Yan Y Sanders

**Affiliations:** Division of Pulmonary, Allergy, and Critical Care Medicine, Department of Medicine, University of Alabama at Birmingham, Birmingham, AL 35294, USA.

**Keywords:** H4K16Ac, Mof, aging, lung fibrosis, fibrotic gene expression

## Abstract

Histone H4 lysine16 acetylation (H4K16Ac) modulates chromatin structure by serving as a switch from a repressive to a transcriptionally active state. This euchromatin mark is associated with active transcription. In this study, we investigated the effects of H4K16Ac on the expression of pro-fibrotic genes in lung fibroblasts from patients with idiopathic pulmonary fibrosis (IPF) and in an aging murine model of lung fibrosis.

**Methods:** The lung tissues and fibroblasts from human IPF/non-IPF donors and from aged mice with/without bleomycin induced lung fibrosis were used in this study. The H4K16Ac levels were examined by immunohistochemistry or western blots. RNA silencing of H4K16Ac acetyltransferase Mof was used to reduce H4K16Ac levels in IPF fibroblasts. The effects of reduced H4K16Ac on pro-fibrotic gene expression were examined by western blots and real-time PCR. The association of H4K16Ac with these genes' promoter region were evaluated by ChIP assays. The gene expression profile in siRNA Mof transfected IPF cells were determined by RNA-Seq. The impact of H4K16Ac levels on lung fibrosis was evaluated in an aging murine model.

**Results:** Aged mice with bleomycin induced lung fibrosis showed increased H4K16Ac levels. Human lung fibroblasts with siRNA Mof silencing demonstrated reduced H4K16Ac, and significantly down-regulated profibrotic genes, such as α-smooth muscle actin (α-SMA), collagen I, Nox4, and survivin. ChIP assays confirmed the associations of these pro-fibrotic genes' promoter region with H4K16Ac, while in siRNA Mof transfected cells the promoter/H4K16Ac associations were depleted. RNA-seq data demonstrated that Mof knockdown altered gene expression and cellular pathways, including cell damage and repair. In the aging mice model of persistent lung fibrosis, 18-month old mice given intra-nasal siRNA Mof from week 3 to 6 following bleomycin injury showed improved lung architecture, decreased total hydroxyproline content and lower levels of H4K16Ac.

**Conclusions:** These results indicate a critical epigenetic regulatory role for histone H4K16Ac in the pathogenesis of pulmonary fibrosis, which will aid in the development of novel therapeutic strategies for age-related diseases such as IPF.

## Introduction

Aging is associated with many chronic diseases, including idiopathic pulmonary fibrosis (IPF) 1. IPF is a progressive and irreversible lung disease with unknown etiology, and usually occurs in aging populations with no effective therapeutic methods 2. IPF is a result of dysregulated lung injury and repair, characterized by extensive scarring of lung tissues. Lung fibroblasts are the main effector cells of lung tissue repair 3. After injury, lung fibroblasts are activated and differentiate into myofibroblasts for wound repair. These myofibroblasts express alpha-smooth muscle actin (α-SMA) 4, with increased Nox4 and H_2_O_2_ production 5, increased extracellular matrix, such as Col1A1 6, and are resistant to apoptosis, which is partially due to the up-regulation of anti-apoptotic genes such as survivin 7.

Although many studies have shown that lung fibroblasts differentiate into myofibroblasts in response to injury and repair 4, the mechanisms are not completely clear. The response of the lung to extrinsic and intrinsic stimuli involves epigenetic processes 8. Epigenetic processes refer to the cellular alterations in response to environmental changes without alterations in DNA sequence 9. One of the major epigenetic regulation processes encompasses histone modification. Histone acetylation is one of the most common modifications of histone tails, which regulates DNA accessibility to various transcriptional factors to control gene expression 10. Histone acetylation is usually associated with euchromatin, whose genomic DNA is accessible for transcriptional complexes to regulate gene expression 11. H4K16 acetylation triggers the unfolding of chromatin by disrupting the interaction between the nucleosomes, indicating active transcription 12. In contrast, deacetylated H4K16 is usually associated with heterochromatin and transcriptional silencing 13. For example, in female mammals, the inactive X chromosome is distinguished by lack of H4K16 acetylation 14.

While there are other lysine sites of H4 that can be acetylated (such as K5,K8, K12), K16 is the first and most frequently acetylated lysine of the H4, which is present in about 60% of the total H4 in mammalian cells 13. Members of the MYST family of histone acetyltransferases (HAT), and class I and class III histone deacetylases (HDACs) control the balance of H4K16 acetylation. Mof (also known as KAT8 or MYST1) is the primary acetyltransferase responsible for H4K16 acetylation 15, and there are at least 5 reported HDACs (HDAC1 and HDAC2 of class I HDAC; SIRT1, SIRT2, SIRT3 of class III HDAC) that deacetylate H4K16Ac 16-19. HDAC1/2 are nuclear enzymes, while SIRT1, 2, and 3 can be located in the nucleus but also shuttle to the cytoplasm (SIRT1 and 2) or mitochondria (SIRT3) 19, 20.

Altered histone acetylation is one of the epigenetic marks changed significantly during the aging process 21. Dysregulated H4K16 acetylation has been implicated in aging 22, 23. In particular, decreased H4K16Ac has been observed in senescent fibroblasts 22. Reduced H4K16Ac levels were also reported to associate with a defective DNA damage response and double-strand break repair 24. However, no studies have explored the role of H4K16Ac in the aging lung in responses to injury and repair or in the age-related disease, IPF. It is also unclear why aged mice with bleomycin injury-induced lung fibrosis have reduced resolution capacity 25. In this study, we investigated the role of H4K16Ac in regulating pro-fibrotic genes in IPF lung fibroblasts and in the persistent lung fibrosis induced by bleomycin in aged mice.

## Materials and Methods

### Cell Culture and Treatments

Human primary IPF and non-IPF lung fibroblasts were derived from de-identified lung tissues (Table S1) from the University of Alabama at Birmingham (UAB) Tissue Procurement Facility, which was approved by the UAB Institutional Review Board. The diagnosis of IPF was made by a multidisciplinary approach according to the ATS/ERS guidelines 26. All primary cells were used before passage 5. The cells were collected for various assays at about 80% confluence.

### siRNA Transfection

siRNA transfections were performed with Opti-MEM (Thermo Fisher Scientific) and lipofectamine RNAi/MAX (Invitrogen). The sequences for Mof siRNA and for non-targeting (NT) are in Table S2. Lung fibroblasts were kept in medium with 10% FBS for 72 h after transfection, then were subjected to various assays.

### RNA extraction and real-time RT-PCR

RNA was extracted with RNeasy Mini Kit (Qiagen, Valencia, CA), and transcribed into cDNA with a cDNA synthesis kit (Takara Bio, Mountain View, CA). Real-time RT-PCR was performed in triplicate and normalized to β-actin using the ΔΔCt method 27. All primers are listed in Table S2.

### RNA sequencing

Total RNA were prepared from three different primary IPF fibroblasts after transfection with either siRNA Mof or NT as described above. RNA-sequencing (RNA-Seq) was performed and data was analyzed at the UAB Genomics Core Facility. RNA-Seq was carried out on the Illumina NextSeq500 according to the protocol from the manufacturer (Illumina Inc., San Diego, CA). More details are online in supplementary data.

### Protein, nuclear extraction and immunoblotting

Whole cell lysates were collected and quantified with a Micro BCA Protein Assay kit (Thermo Scientific). Nuclear extracts were collected with EpiQuick Nuclear Extraction kit (Epigentek, Farmingdale, NY), then quantified. Lysates or nuclear extracts were subjected to SDS-PAGE and western immunoblotting (WB) as described before 28. The following antibodies were used: Anti-Mof (A300-992A) from Bethyl Laboratories (Montgomery, TX); anti-α-smooth muscle actin (03-61001) from ARP, anti-Nox4 (AF8158) from R&D systems (Minneapolies, MN), anti-Col1A1(cat# GTX82720) from Genetex (Irvine, CA); anti-survivin (#2808), anti-β-actin (#2128), and anti-H3 (#9715) were from Cell signaling (Beverly, MA), anti-H4K16Ac(#61529) and anti-H4K20Me3 (#39671) were from Active Motif (Carlsbad, CA). Immunoblots were imaged with an Amersham Biosciences 600 Imager (GE Healthcare). Densitometry analysis was done using Image J software.

### Immunohistochemistry

Sections from paraffin-embedded lung tissues were immunostained with anti-H4K16Ac antibody (ab109463) from Abcam (Cambridge, MA). Briefly, slides from paraffin block were rehydrated with xylene, ethanol, and water. After heat induced epitope retrieval in Thermo citrate buffer (Thermo Scientific, Cheshire, WA), slides were immunostained according to the Dako Rabbit Envision System (Dako, Carpinteria, CA) protocol. Images were obtained with a Keyence BZ-X700 All-in-one microscope (Itasca, IL).

### Chromatin immunoprecipitation assays

Chromatin immunoprecipitation (ChIP) assays were performed as per the manufacturer's protocol (ab500, Abcam, Cambridge, MA) with minor modifications 29. An antibody against H4K16Ac (Active Motif #61529) was used to pull down the associated DNA. ChIP-DNA were amplified by real-time PCR with primers listed in Table S2, using SYBR® Green PCR Master Mix (Life Technologies, Carlsbad, CA). Results were normalized to input DNA.

### Aged mice model of bleomycin induced lung fibrosis

All animal studies were performed in accordance with UAB Institutional Animal Care and Use Committee approved protocols. 18-month old healthy C57BL mice were used for the study. A single dose of normal saline or bleomycin sulfate at 1.5 U/kg body weight was instilled intratracheally. Mouse siRNA Mof (mMof) or NT control (sequence list in Table S2, ordered from Invitrogen) were given intranasally every 3 days at 50 μg in 75 μl PBS starting week 3 to week 6 after bleomycin injury. Whole lung tissues were prepared for whole-lung lysates, or nuclear extract, or subjected to hydroxyproline assays. Some of the mice lung tissues were prepared for histology with H&E or trichrome stains. The nuclear extracts of primary lung fibroblasts cultured at passage 1 were prepared from 18-month old mouse lung tissues 6-week after saline or bleomycin injury.

#### Hydroxyproline analysis

Mice lung tissues were collected and hydroxyproline content was analyzed using a kit from QuickZyme BioSciences. Briefly, lung tissues were dried in an oven at 70 C for 48 h, and then hydrolyzed in 6 N HCL at 95 C for 20 h. The hydroxyproline content was measured following the manufacturer's protocol using hydroxyproline as a standard.

### Statistical Analysis

Data are presented as the mean ± standard deviation (SD). All data were statistically analyzed using GraphPad Prism 5.0 (La Jolla, CA). One-way ANOVA was used to compare between multiple groups; comparison between two groups were performed using Student's t test or nonparametric Mann Whitney test. A *P* value of <0.05 was considered to be statistically significant.

## Results

### Bleomycin injury altered H4K16Ac levels in lung tissues of 18-month old mice

**Figure 1:**
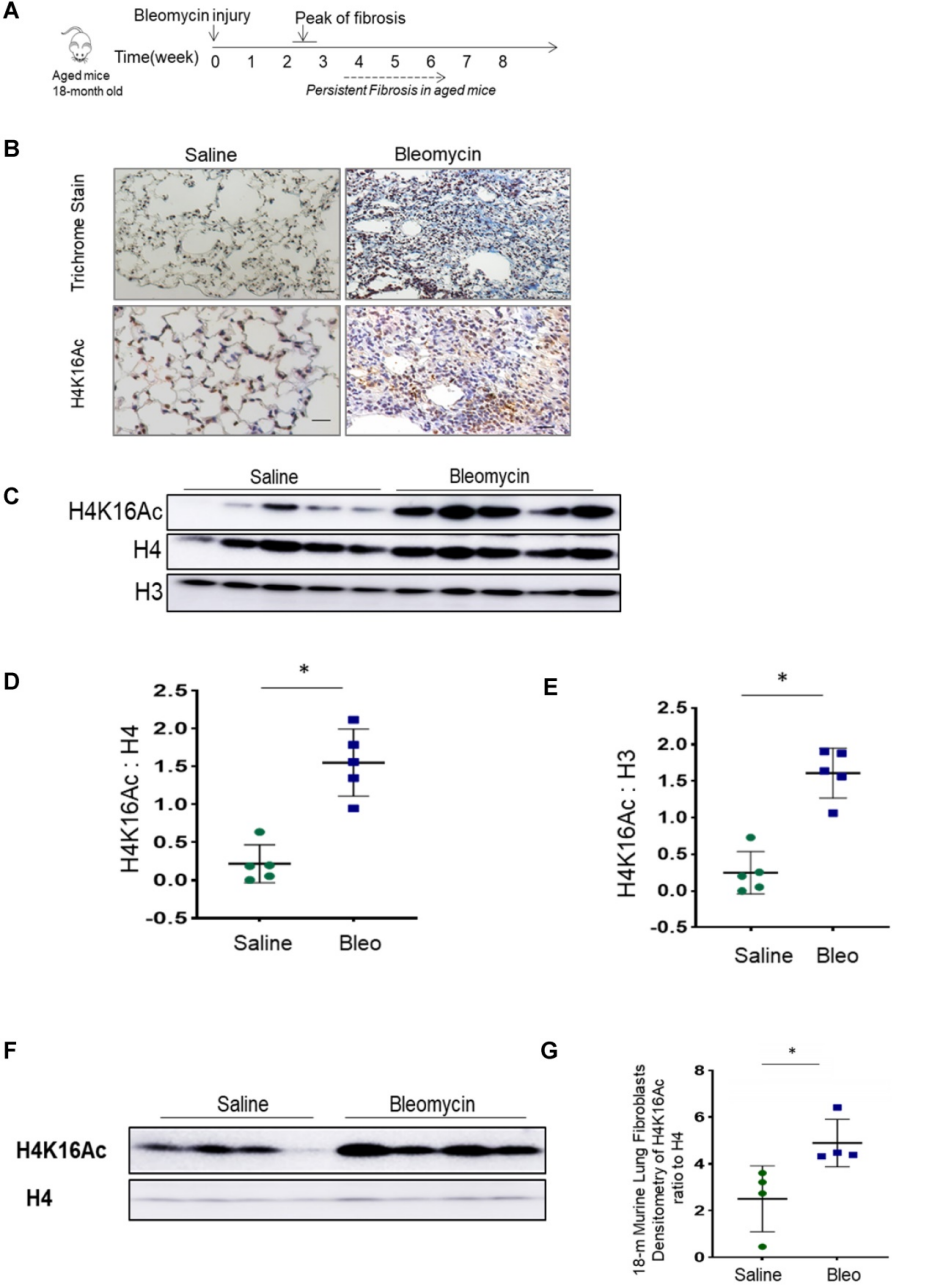
** Histone H4K16Ac levels in aged mice subjected to bleomycin injury. (A)** The schedule of induced lung fibrosis by bleomycin injury in 18-month old mice. **(B)** Trichrome stain and immunohistochemistry stain of H4K16Ac in 18-month old mouse lung tissues six weeks after saline (left) or bleomycin (right) injury. H4K16Ac stained in brown. Nuclei stained with DAPI in blue. Scale bar: 100 µm. **(C)** Histone H4K16Ac levels in lung tissues of 18-month old mice six weeks after saline or bleomcyin injury by western blots. H4 or H3 servers as loading control. **(D and E)** Densitometry of H4K16Ac relative to H4 (D) or H3 (E) as shown in C. **(F)** Primary lung fibroblasts cultured from 18-month old mice after subjected to 6 weeks of saline or bleomycin injury. Nuclear extracts are subject to western blots to examine the H4K16Ac levels in lung fibroblasts, H4 as loading control. **(G)** Densitometry of H4K16Ac relative to H4 as shown in F. *P < 0.05, bleomycin injured group *vs* saline group.

During normal aging, hypoacetylation of H4K16 is reported 22, 30, 31. We assessed the H4K16Ac levels in lung tissues of 18-month old mice at baseline and after bleomycin injury. Lower levels of H4K16Ac were noticed in the lung tissues and in lung fibroblasts of aged mice when compared to the young ones at baseline (supplementary Figure S1A-B). In persistent lung fibrosis model, the aged mice with bleomycin injury have impaired resolution of lung fibrosis 25. In this model at 6-week post injury (Figure 1A), these 18-month old mice with non-resolving lung fibrosis 32 showed elevated H4K16Ac levels in the lung tissues by immunohistochemistry (Figure 1B) and western blots (Figure 1C-E, and Figure 1F-G for isolated primary lung fibroblasts). These data suggest that the aged mice with non-resolving lung fibrosis exhibited increased levels of H4K16Ac.

### H4K16Ac levels in lung tissues and fibroblasts of human IPF subjects

**Figure 2:**
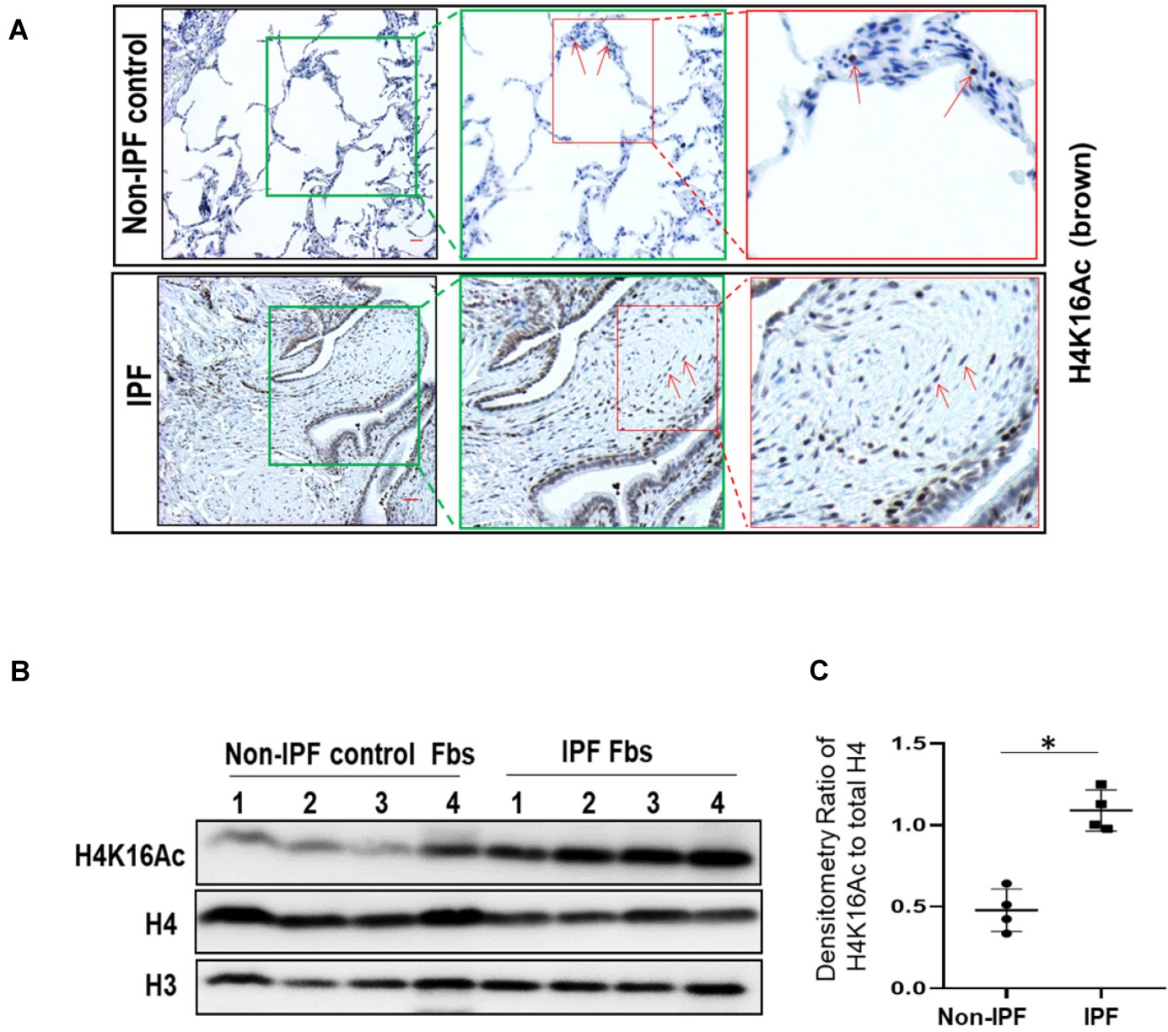
** Histone H4K16Ac in non-IPF and IPF lung tissues and fibroblasts. (A)** Immunohistochemistry stain of H4K16Ac (brown) with lung tissues from non-IPF and IPF subjects. Red arrows indicate examples of positive stains of H4K16Ac. The green or red squares indicate the enlarged area. Scale bar: 100 µm. **(B)** Western blots of H4K16Ac in primary human lung fibroblasts (Fbs) from non-IPF or IPF subjects. H3 or H4 is for loading control. **(C)** Densitometry of H4K16Ac relative to H4 as indicated in B. *P < 0.05, IPF *vs* non-IPF (n = 4 in each group).

We then examined H4K16Ac levels in human IPF lungs. By immunohistochemistry, the non-IPF lung tissues have less H4K16Ac staining than the IPF lung tissues (Figure 2A, top panel). In contrast, IPF lung tissues exhibited increased H4K16Ac staining in fibroblasts, as well as surrounding epithelial cells, and in the fibroblastic foci of IPF lung tissue (Figure 2A, lower panel and online Figure S2). We further examined H4K16Ac levels in primary human lung fibroblast cultures. Compared to the non-IPF primary lung fibroblasts, we noticed significantly increased levels of H4K16Ac in the IPF fibroblasts that we examined (Figure 2B-C). These data indicate that the H4K16Ac levels are increased, at least in some, IPF lung tissues and fibroblasts, which is similar to our *in vivo* murine model of persistent lung fibrosis.

### Knockdown of Mof decreases H4K16Ac levels and down-regulates the expression of multiple pro-fibrotic genes associated with H4K16Ac in IPF lung fibroblasts

**Figure 3:**
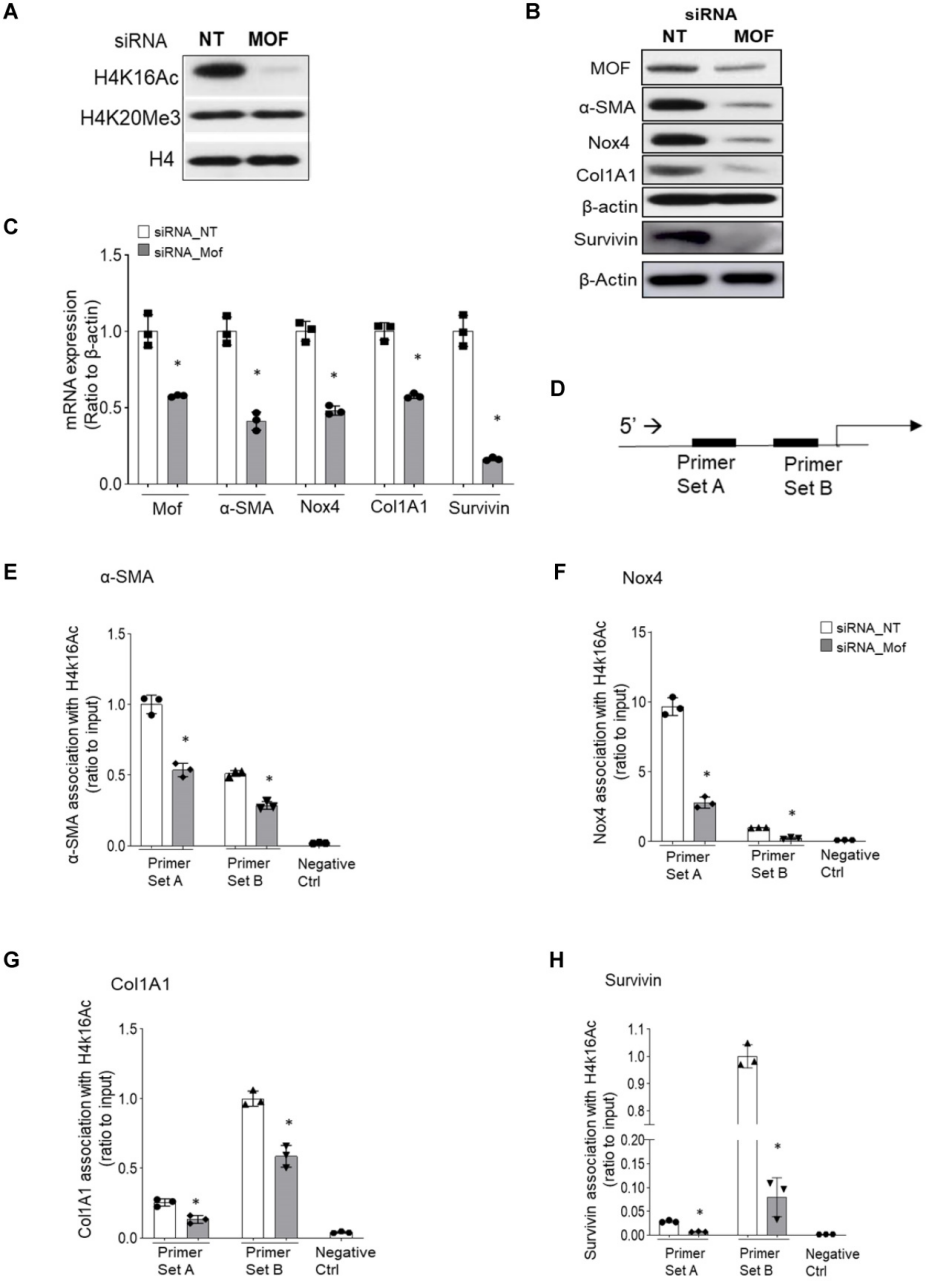
** Silencing Mof decreases H4K16Ac levels, and down-regulates multiple pro-fibrotic genes that are associated with H4K16Ac in IPF lung fibroblasts.** Representative figures of IPF lung fibroblasts with siRNA non-targeting (NT) control or Mof. **(A)** Histone H4K16Ac and H4K20Me3 levels were examined by western blots with histone extracts. H4 is loading control. Mof expression in these cells is shown in B. **(B)** The whole cell lysate were collected for western blots (Survivin is on a separate blot, the Mof knockdown was shown in online Figure S3B); and (**C**) RNA were collected for real-time RT-PCR to examine pro-fibrotic genes α-SMA, Nox4, Col1A1 and survivin expression at protein (B) or mRNA (C) levels. **(D)** Schematic of the relative locations of the PCR primers sets used to examine the association of H4K16Ac and the promoter regions with the genes listed in E-H by ChIP assays, primer sequences are listed in Table S2. **(E-H)** Primary IPF lung fibroblasts with siRNA NT or Mof (treated as in A), were subject to ChIP assays. DNA was immunoprecipitated with H4K16Ac antibody. The relative levels of PCR products were represented by bars of the association of H4K16Ac with promoter region of α-SMA (E), Nox4 (F), Col1A1 (G) or Survivin (H). Quantitative PCR data were analyzed by 2^ΔΔCt^ method, normalized to input DNA, expressed as fold changes relative to primer set A or B. Negative control represents IgG pull-down. The values are expressed as mean ± SD from average of three independent experiments of one representative cell line. *P < 0.05, siRNA Mof *vs* siRNA NT control.

H4K16Ac levels are controlled by Mof, one of the major acetyltransferases 15, and by at least five reported deacetylases 16-19. Either decreased acetyltransferase or increased deacetylase could contribute to the down-regulation of H4K16Ac levels. In this study, we examined if knockdown the major acetyltransferase Mof by RNA silencing in IPF patient lung fibroblasts could reduce H4K16Ac levels and affect the expression of some pro-fibrotic genes. Mof knockdown significantly decreased H4K16Ac levels, while other histone modifications, such as H4K20me3 levels did not appear to be affected (Figure 3A, also online Figure S3A). We further determined if some of the well-documented pro-fibrotic markers are down-regulated directly due to the decreased levels of H4K16Ac.

The pro-fibrotic genes α-SMA, Nox4, Col1A1, and survivin all have been reported to be up-regulated in IPF fibroblasts 5, 7, 33. In Mof silenced IPF fibroblasts that showed decreased H4K16Ac levels (Figure 3A), significantly down-regulation of these markers at protein (Figure 3B) and mRNA (Figure 3C) levels were noticed when compared to the siRNA non-targeting (NT) control. We then examined if the down-regulation of the pro-fibrotic markers (α-SMA, Nox4, Col1A1 and survivin) are due to the decreased association of H4K16Ac with their promoter region. We used two sets of primers for each gene at the promoter region (Figure 3D, all primers sequences in online Table S2) and determined their association with H4K16Ac by ChIP assays. These four well-established pro-fibrotic genes all showed depleted association with H4K16Ac in Mof silenced IPF fibroblasts compared to the NT control (Figure 3E-H). The enrichment of H4K16Ac varied at different gene and region, which may suggest H4K16Ac participating differentially in regulating these target genes. For instance, we noticed a nearly 5-fold enrichment of H4K16Ac at the primer set A than set B region of Nox4, while both regions showed significantly reduced binding of H4K16Ac with siRNA Mof compared to the NT control (Figure 3F). We also noticed at the set B region, the association of H4K16Ac with Col1A1 promoter region decreased nearly 20% in siRNA Mof cells; however, H4K16Ac binding with survivin decreased nearly 80% in siRNA Mof cells (Figure 3G-H). These data indicate that H4K16Ac is directly involved in regulating the expression of these pro-fibrotic genes, however, at varied degrees.

### Silencing Mof alters gene expression profile in IPF lung fibroblasts

**Figure 4:**
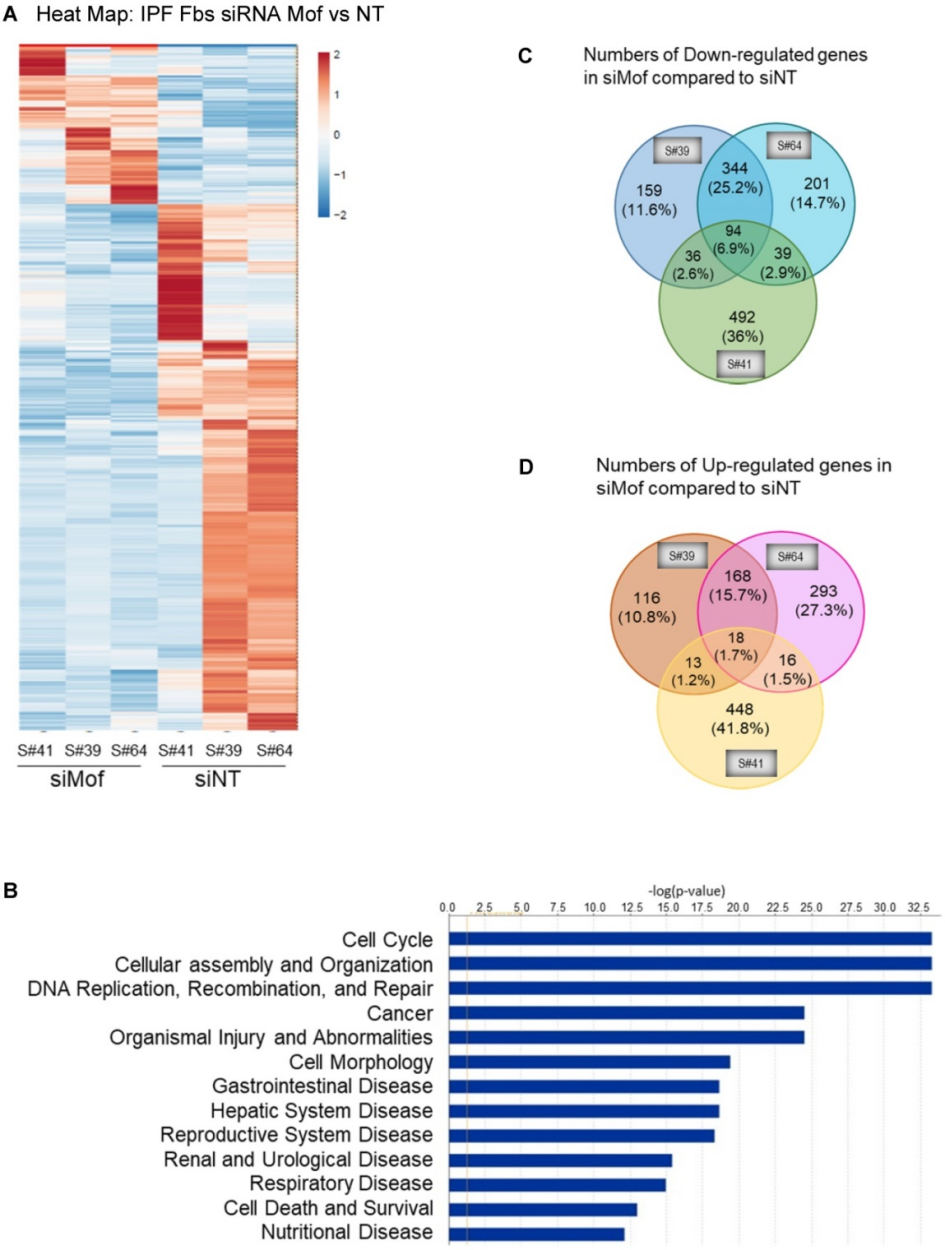
** RNA-Seq data with IPF lung fibroblasts transfected with siRNA Mof or NT control.** Three different IPF primary lung fibroblast cell lines were transfected with siRNA Mof or NT control. The RNA were collected after 3 days of transfection (as in Figure 3B) and subjected to RNA-Seq. **(A)** Heatmap showed the differentially expressed genes in siRNA Mof *vs* NT transfected cells. **(B)** Analysis of altered genes in top categories according to biological functions from siRNA Mof vs NT (fold change ≥ ± 2 & p-value < 0.05). **(C)** The Venn diagram of down-regulated genes of three different cell lines with siRNA Mof compared to siRNA NT. There are 94 genes are down-regulated in all three cell lines silenced with siRNA Mof, while 344 genes are down-regulated in both sample #39 and #64, 36 in sample #39 and #41, 39 in sample #41 and #64. **(D)** The Venn diagram of the genes are up-regulated in siRNA Mof compared to siRNA NT. There are 18 genes that are up-regulated in all three cell lines silenced with siRNA Mof, while 168 genes are up-regulated in sample #39 and #64, 13 in sample #39 and #41, 16 in sample #41 and #64. All genes analyzed are fold change ≥ ±2, and p value < 0.05. More RNA-Seq data are in online supplementary materials.

To obtain a genome-wide transcriptomic overview of altered gene expression in IPF lung fibroblasts by Mof silencing, we transfected siRNA Mof or NT control in lung fibroblasts from three different IPF donors as in Figure 3, then subjected them to RNA-sequencing. The data showed marked heterogeneity of these individual IPF lung fibroblasts (Figure 4). However, we observed differentially expressed genes in at least two of the three cell lines transfected with Mof compared to controls (Figure 4A, overall heatmap). Despite the heterogeneity of the cells, we observed that most altered gene expression patterns were involved in cellular assembly and organization/DNA replication, recombination, repair/post-translational modification and cell cycle (Figure 4B, and online supplementary data Figure S4, Figure S5, and Table S3, data are analyzed with n = 3 of each category). Among the genes that showed significant expression differences, 94 genes were down-regulated (Figure 4C) while 18 genes were up-regulated (Figure 4D) in all three Mof knocked-down compared to their control cells. These data confirmed that Mof is critical for cell damage and repair 24, as well as post-translational modifications 34, such as to control H4K16Ac levels. The RNA-seq data indicates that besides many down-regulated genes with Mof knockdown, there are up-regulated genes, likely due to secondary effects. In summary, silencing Mof alters gene expression profiles in human IPF lung fibroblasts.

### Improved lung fibrosis resolution by mMof knockdown in lungs of aged mice with established lung fibrosis

**Figure 5:**
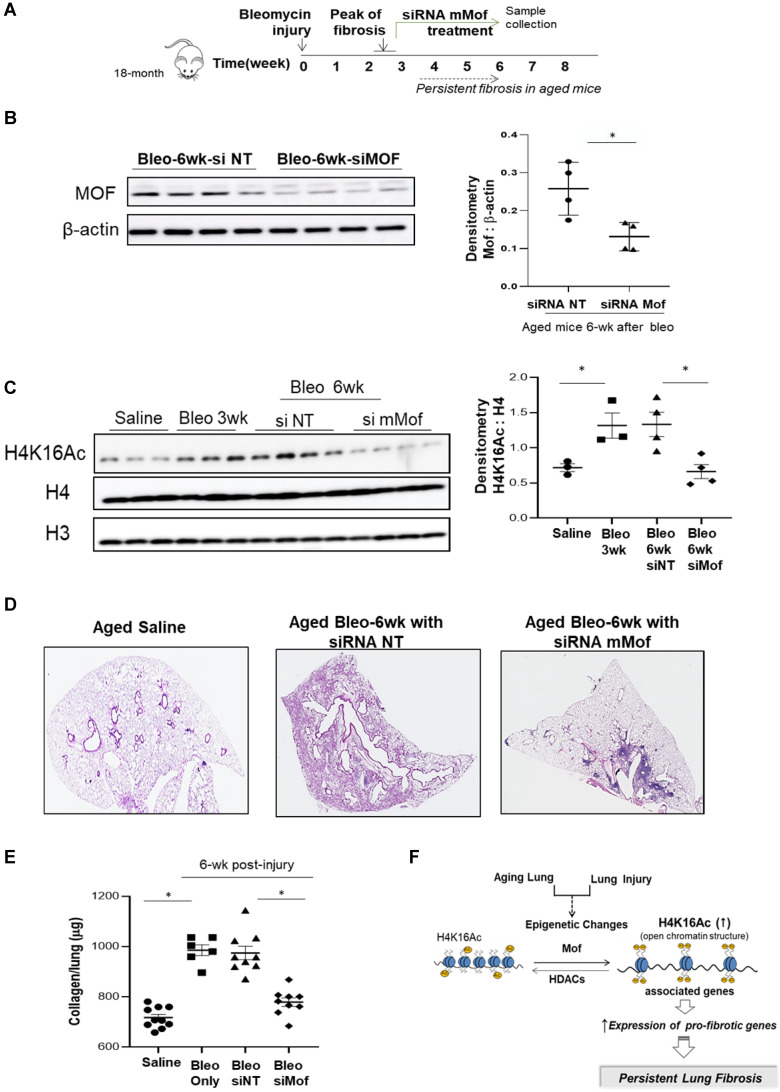
** Inhibition of Mof improves the resolution of lung fibrosis in aged mice. (A)** Schematic timeline of drug administration and sample collection. Lung fibrosis was induced by bleomycin injury in 18-month old mice. Treatment with siRNA mMof or NT was given every 3 days at 50 μg in 75 μl PBS from week 3 to 6 after bleomycin injury. Samples were collected at the end of week 6 after bleomycin injury. **(B)** Mice lung tissues were collected and Mof expression were examined by western blot after 6-week in the bleomycin injured mice group treated with siRNA NT or siRNA mMof. β-actin is the loading control. Right, the densitometry of Mof relative to β-actin as shown on the western blot on the left. *P < 0.05, siRNAT NT group vs siRNA Mof group. **(C)** Nuclear extract from mice lung tissues, examined with the levels of H4K16Ac under conditions as marked, loading control is H4 or H3. Right, densitometry of H4K16Ac to H4 as shown on the western blots on the left. *P < 0.05, saline group vs 3-week bleomycin injured group, or 6-week after bleomycin injured groups treated with siRNAT NT group vs siRNA Mof group. **(D)** Lung tissue histology by H&E stain in lungs of 6-week after saline or bleomycin injured mice with siRNA NT or siRNA mMof treatment. **(E)** Hydroxyproline content in lungs of mice 6-week after subjected to saline, bleomycin, bleomycin with siRNA NT or bleomycin with siRNA mMof, by mean ± SD. *P < 0.05, for comparisons of indicted groups as compared with the saline group, or bleomycin treated with siRNA mMof to siRNA NT. **(F)** Schematic diagram of possible mechanisms of the findings from this study. Injury in aging lung causes epigenetic changes, such as increased H4K16Ac levels, which will result in up-regulation of some pro-fibrotic genes and eventually lead to the development of persistent lung fibrosis.

Since we observed increased H4K16Ac levels in aged mice with persistent lung fibrosis (Figure 1), we then evaluated whether lowering H4K16Ac levels in the aged mice with established lung fibrosis would improve fibrosis resolution. We first examined the murine Mof (mMof) siRNA knockdown efficacy in primary murine lung fibroblasts, which demonstrated efficient Mof knockdown and noticeable down-regulated H4K16Ac levels in these cells (supplementary data Figure S6). We induced lung fibrosis in 18-month old mice by intra-tracheal instillation of bleomycin. The mice developed lung fibrosis persistent up to two months or longer after the injury 25. To confine the knockdown of mMof to the lungs, intranasal delivery of stealth siRNA against mMof (NT for control) was given to the bleomycin injured aged mice every 3 days starting week 3 after the injury, when the lung fibrosis is established. The treatment lasted to week six, when the samples were collected (Figure 5A). mMof knock down was confirmed by western blots showing significantly decreased mMof in the mice lungs treated with siRNA targeting mMof (Figure 5B), which also showed significantly decreased levels of H4K16Ac compared to the control group (Figure 5C). The mice in siRNA mMof treated group showed improved resolution of lung fibrosis 6-week after bleomycin injury (Figure 5D and Figure S7). The whole lung collagen content was assessed by measuring hydroxyproline content from the whole lung tissues. The siRNA mMof treated group showed significantly decreased hydroxyproline compared to the control siRNA NT group after 6-week of bleomycin injury (Figure 5E). These data support the concept that reduced H4K16Ac levels could improve the resolution of persistent lung fibrosis in the aged mice.

## Discussion

Epigenetic alterations are involved in aging and age-related diseases 35, in particular, H4K16Ac plays an important role in the aging process 22, 30. H4K16Ac marks actively transcribed genes 36, regulates chromatin remodeling and global activation of gene expression 37. In this study, we showed that H4K16Ac levels are elevated in aged mice lung tissues after six weeks of bleomycin injury induced lung fibrosis, which may indicate persistent repair processes in the aged mice. H4K16Ac levels were also increased in the human IPF lung tissues and fibroblasts that we examined, likely reflecting an injury and repair response, and parallel the up-regulation of related genes in myofibroblasts 38. Silencing H4K16 acetyltransferase Mof in IPF fibroblasts resulted in decreased H4K16Ac levels, reduced binding of H4K16Ac with pro-fibrotic gene promoter regions, and reduced expression of these genes. In the aging mice model of persistent lung fibrosis, nasal delivery of siRNA mMof reduced H4K16Ac levels in the lungs leading to improved lung fibrosis resolution. Our data suggest that injury in the aging lung (a “double hit”) increases H4K16Ac levels to activate and sustain the transcription of, at least some, pro-fibrotic genes and ultimately result in persistent lung fibrosis (Figure 5F).

Histone post-translational modifications are critical for chromatin structure, which affect many biological processes, such as DNA transcription, replication and repair 39. Histone acetylation is consistently associated with transcription activation 3. Previous studies have demonstrated that deacetylation of H4K16Ac contributes to DNA compaction during senescence 22. In a progeroid mouse model, premature aging was associated with decreased H4K16Ac levels 30. The decreased H4K16Ac levels in senescent cells may suggest the increased heterochromatic state of these cells. Despite the general levels of decreased H4K16Ac in senescent cells, H4K16Ac can be enriched at specific genomic loci 29, or be redistributed 23. A study comparing H4K16Ac distribution in Alzheimer's disease patients with young and old normal controls of the lateral temporal lobe revealed that H4K16Ac was redistributed with aging, while subjects with Alzheimer demonstrated a different pattern of H4K16Ac redistribution when compared to normal aging 23. We observed reduced H4K16Ac levels in mice lung tissues during normal aging, but increased levels were seen in aged mice with persistent lung fibrosis.

Although H4K16Ac levels are increased, changes associated with acetylation of other histones in this lung fibrosis model have not been determined. Many studies have shown that HDAC inhibitors are beneficial in pre-clinical models in treating lung fibrosis; however the mechanisms are not defined, and no study has examined the effects of HDAC inhibitors on H4K16Ac in lung fibrosis 3, 40. The HDAC inhibitors not only affect histones but also numerous non-histone proteins that may directly affect master regulatory proteins to alter gene expression 41. HDAC inhibitors may affect the acetylation of other histone lysines besides H4K16 or other proteins, which might benefit for lung fibrosis resolution. In addition, the timing at which HDAC inhibitors are given in these models is also critical. Different intervention times could generate diverse effects, which depends whether the purpose was to study prevention, reduction of inflammation, or targeting resolution of established fibrosis. In this study, the treatment was given after established fibrosis to target fibrosis resolution. Currently, it is unknown which specific histone acetylation drive the development, persistence or progression of IPF, neither is clear if pro-fibrotic genes are regulated by the enriched acetylated histone induced by HDAC inhibitors. Our study is the first to show that reduced H4K16Ac contributed to decrease pro-fibrotic gene expression as demonstrated in ex-vivo fibroblasts from IPF patients.

As mentioned above, Mof is only one of multiple enzymes that regulate H4K16Ac levels 13, 16-19. The upregulated H4K16Ac levels could be directly related to increased acetyltransferases, decreased deacetylases, or both. The H4K16Ac levels controlled by Mof are critical. A recent report found that H4K16Ac deficiency caused by Mof mutation directly links to epilepsy 42. Although Mof knockdown resulted in decreased H4K16Ac levels and altered the expression of genes that are associated with this histone modification, Mof itself may directly regulate gene expression 43. However, we provide direct evidence with ChIP assays that the downregulated pro-fibrotic genes by Mof knockdown in this study is due to the depleted H4K16Ac at these genes' promoter regions. This shows that H4K16Ac directly participates in regulating pro-fibrotic gene expression in IPF lung fibroblasts.

The observed increase in H4K16Ac levels in fibrotic lung tissues may contribute to an over-exuberant repair process. Increased H4K16Ac levels have been linked with increased DNA repair at gene-rich regions 44. In the animal model of lung fibrosis, silencing Mof at the peak of lung fibrosis to reduce H4K16Ac in the lung tissues demonstrated the feasibility of lowering H4K16Ac locally and temporarily. However, this may not be practical in clinical settings. Mof is not only critical for embryonic development, but also regulates essential cellular functions such as cell cycle progression 15, 45 and DNA damage responses 24, 46, 47. We have explored other methods to block the increased acetylation of histone at pro-fibrotic gene loci in another study. We tested the efficacy of an “epigenetic reader” bromodomain inhibitor to mitigate downstream effects of increased H4K16Ac, and successfully decreased the associated expression of pro-fibrotic gene 32.

IPF is an age-related disease 1. Until recently, many studies have used young mice only as model system for bleomycin-induced lung fibrosis. Interestingly, this model has been shown to undergo fibrosis resolution roughly 2-month after bleomycin injury 25. Our data shows that the young and aged mice have different basal levels of H4K16Ac. It is likely that young and aged mice have distinct injury/repair responses as well as differential H4K16Ac levels. The aging mouse model system with persistent lung fibrosis would have differential gene expression and overall changes, making it difficult to compare to the young mouse model. This study focuses on the aging mouse model of lung fibrosis. We showed improved fibrosis resolution with Mof knockdown in the lung tissues by nasal delivery. However, one limitation of this method is the inability to discriminate specific cell types that are more susceptible to the nasal delivered siRNA knockdown. Although previous study from our group showed that nasal delivered siRNA would knockdown target gene in lung fibroblasts 25, other studies indicated that nasal delivered siRNA has whole lung effects 48. It is possible that different cell types were knockdown by Mof, which may all contribute to the improved fibrosis resolution. Cell-type specific knockdown, such as lung fibroblast specific Cre-mediated conditional knockdown in animal studies, is needed to elucidate the role of Mof in specific cell type.

Fibroblasts in IPF are well-known for their heterogeneity 49. The current study was focused on cells with increased H4K16Ac levels, while we also noticed sub-populations of IPF lung fibroblasts with lower H4K16Ac levels (data not shown). In addition, primary lung fibroblasts from different subjects have different levels of H4K16Ac. When silencing Mof, different cell line showed differential responses. Regardless of these differences, all cells we examined with siRNA Mof, showed similar results with decreased fibrotic gene expression and depleted H4K16Ac at the promoter region of these genes. We acknowledge that our RNA-Seq sample size is small, and an increase in the sample population is needed to validate our findings. Nonetheless, our data indicate a critical role of H4K16Ac in the pathogenesis of IPF, and provide support for more extensive studies to discover the role of histone H4K16Ac in lung fibrosis.

Overall, our study demonstrates that histone H4K16Ac regulates a group of pro-fibrotic genes in age-related lung fibrosis, and the potential importance of developing personalized epigenetic modifiers as targeted therapies. Aging and environmental stress are associated with epigenetic changes that are crucial for the fibrotic process. Targeting disease-specific epigenetic changes in IPF would provide a novel therapeutic strategy for this fatal lung disease.

## Supplementary Material

Supplementary methods, figures, tables 1-2, and table 3 heading.

Supplementary table 3.
