# Supplementary material for: Modulation of H4K16Ac levels reduces pro-fibrotic gene expression and mitigates lung fibrosis in aged mice
Source: Theranostics. 2022 Jan 1;12(2):530–41. doi: 10.7150/thno.62760 (PMC8692895; doi:10.7150/thno.62760)
Supplement: Supplementary file 1 — Supplementary methods, figures, tables 1-2, and table 3 heading. [file thnov12p0530s1.pdf]

Modulation of H4K16Ac levels reduces pro-fibrotic gene expression and mitigates lung fibrosis in aged mice

Xiangyu Zhang, Hui Liu, Jennifer Q Zhou, Stefanie Krick, Jarrod W Barnes, Victor J Thannickal, Yan Y Sanders *

Division of Pulmonary, Allergy, and Critical Care Medicine, Department of Medicine, University of Alabama at Birmingham, Birmingham, AL 35294, USA

*Correspondence author:

Yan Y Sanders, email: yans@uab.edu

Online Supplementary Materials:

Methods:

RNA sequencing

Total RNA were prepared from three different primary IPF fibroblasts after transfection with either siRNA Mof or NT as described. RNA-sequencing (RNA-Seq) was performed on the Illumina NextSeq500 following the manufacturer's protocol (Illumina Inc., San Diego, CA) at the UAB Genomics Core Facility.

Briefly, RNA quality was assessed using the Agilent 2100 Bioanalyzer. RNA with a RNA Integrity Number (RIN) of ≥7.0 was used for sequencing library preparation. RNA passing quality control was converted to a sequencing ready library using the Agilent SureSelect Strand Specific mRNA library kit as per the manufacturer's instructions (Agilent, Santa Clara, CA). The cDNA libraries were quantitated using qPCR in a Roche LightCycler 480 with the Kapa Biosystems kit for Illumina library quantitation (Kapa Biosystems, Woburn, MA) prior to cluster generation. Cluster generation was performed according to the manufacturer's recommendations for onboard clustering (Illumina, San Diego, CA). We generated between 30-35 million paired end 75bp sequencing reads per sample for transcript level abundance.

Data assessment:

STAR (version 2.5.3a) was used to align the raw RNA-Seq fastq reads to the reference genome from Gencode [1]. Following alignment, HTSeq-count was used to count the number of reads mapping to each gene [2]. Normalization and differential expression was then applied to the count files using DESeq2 [3].

Supplementary Reference:

1. Dobin, A., et al., *STAR: ultrafast universal RNA-seq aligner.* Bioinformatics, 2013. **29**(1): p. 15-21.
2. Anders, S., P.T. Pyl, and W. Huber, *HTSeq--a Python framework to work with high-throughput sequencing data.* Bioinformatics, 2015. **31**(2): p. 166-9.
3. Love, M.I., W. Huber, and S. Anders, *Moderated estimation of fold change and dispersion for RNA-seq data with DESeq2.* Genome Biol, 2014. **15**(12): p. 550.

Supplementary Figures:

A

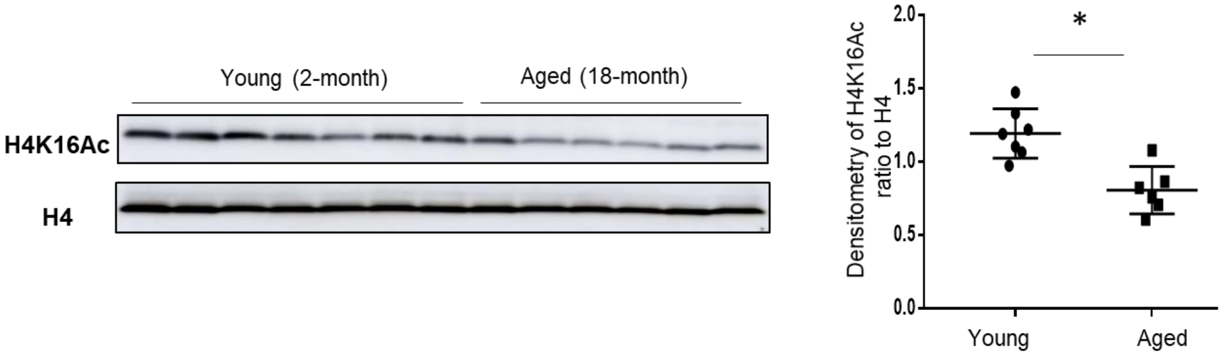

B

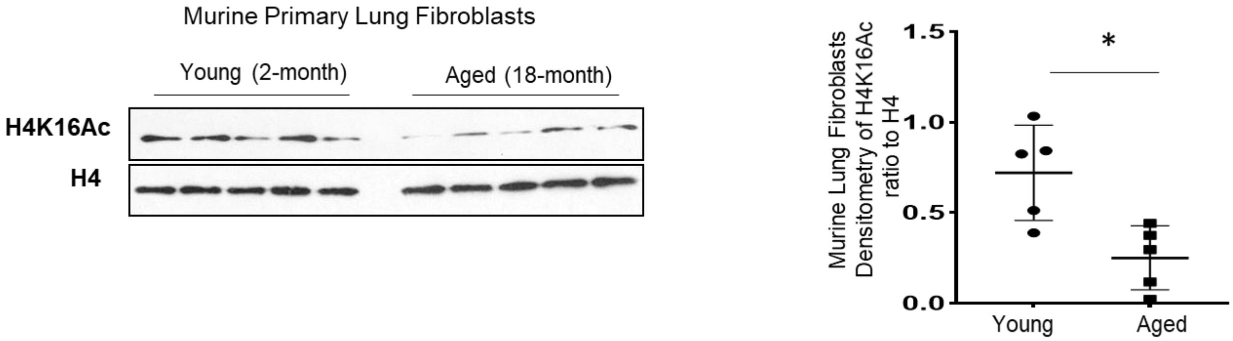

Figure S1. Histone H4K16Ac levels in young and aged mice lung tissues / primary murine lung fibroblasts.

(A) The H4K16Ac levels in young (2-month old) and aged (18-month old) mice lung tissues at baseline by western blots, H4 is the loading control. *Right,* Densitometry of H4K16Ac relative to H4, as in A. *P < 0.05, aged (n = 6) compared to young (n = 7) mice. **(B)** Baseline levels H4K16Ac and H4 by WB in lung fibroblasts isolated from young (2-month) or aged (18-month) mice. *Right,* Densitometry of H4K16Ac relative to H4 as in C. *P < 0.05, n = 5 in each group.

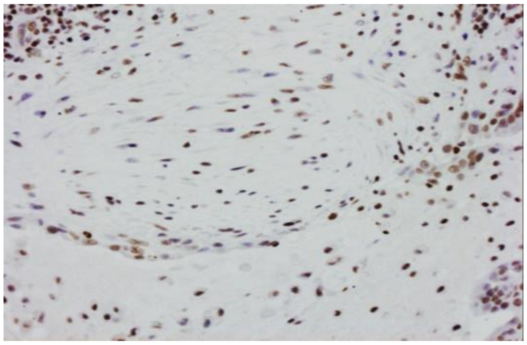

Figure S2. IPF lung fibrotic focus stain of H4K16Ac (brown), picture taken at 20 x.

A

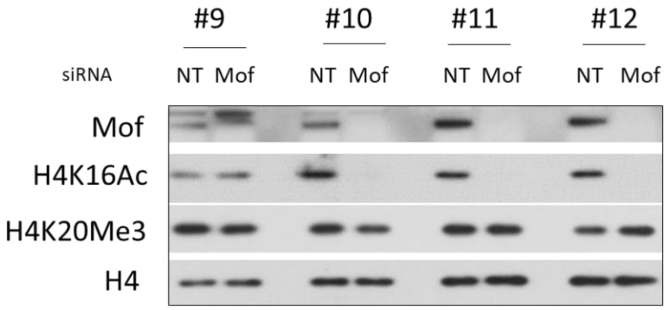

B

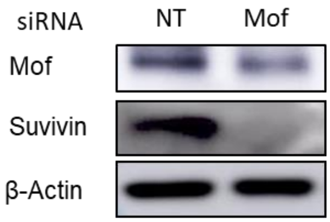

Figure S3. (A) Four different siRNA Mof from Thermo Scientific were tested for knocking down Mof in lung fibroblasts. # 12 was used in the rest of the experiments. Western blots demonstrated the Mof, histone H4K16Ac, and H4K20me3 levels in nuclear extracts. H4 is the loading control. **(B)** IPF fibroblasts transfected with siRNA or NT, western blots demonstrated the Mof knockdown for the blot show in Figure 3B for survivin expression by western blot.

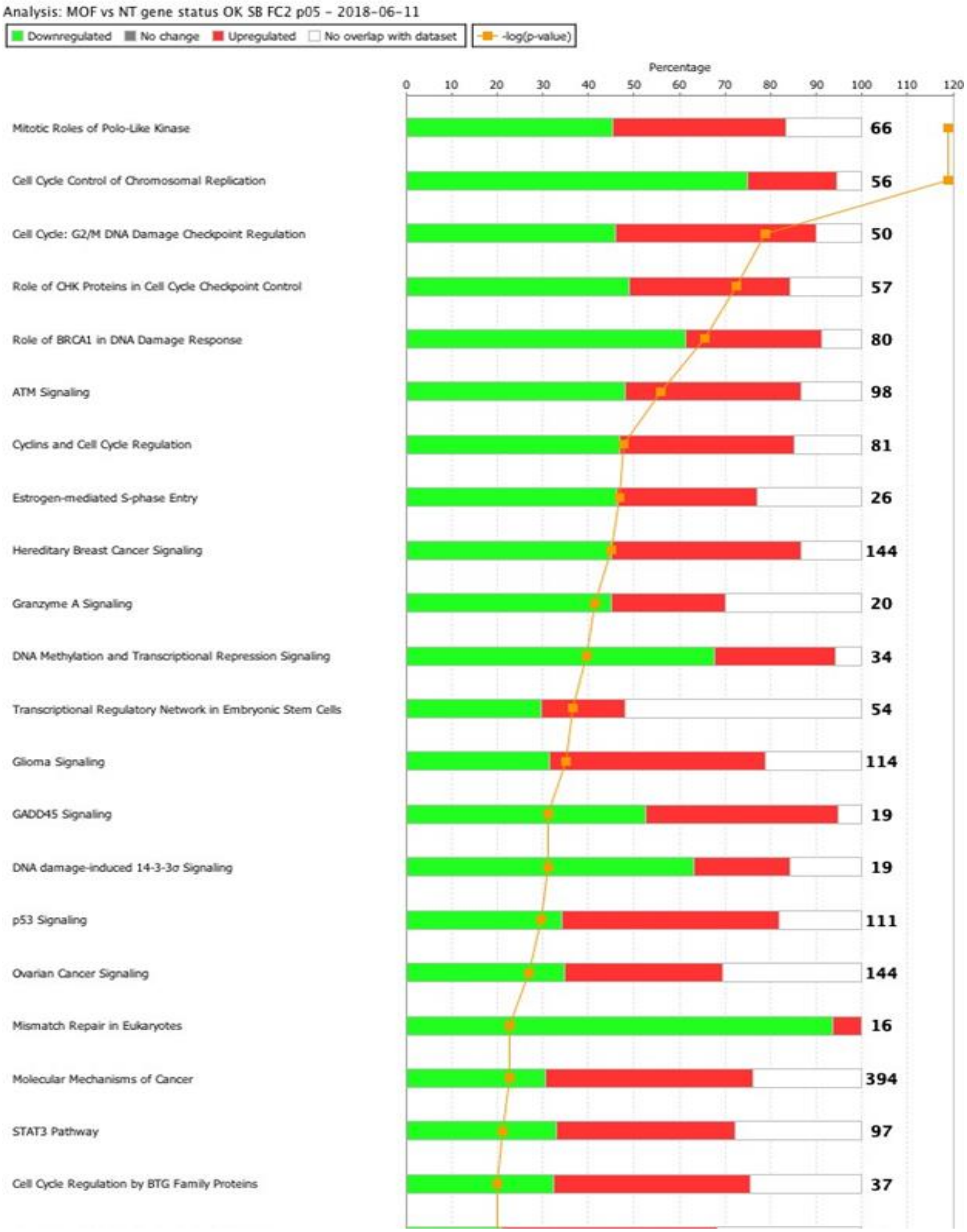

Figure S4. Pathways of IPF fibroblasts transfected with siRNA Mof vs NT by RNA-Seq from 3 different IPF fibroblast cell lines.

ID	Analysis	Molecules in Network	Score	Focus Molecu	Top Diseases and Functions
1	MOF vs NT gene status OK SB FC2 p05 - 2018-06-11	↓ASF1B, ↓C21orf58, ↓CHAF1B, ↓GRB2, ↓H3F3A/H3F3B*, ↓HIST1H2AB, ↓HIST1H2AD, ↓HIST1H2AH, ↓HIST1H2AJ, ↓HIST1H2BB, ↓HIST1H2BH, ↓HIST1H2BM, ↓HIST1H2BO, ↓HIST1H3A, ↓HIST1H3C, ↓HIST1H3E, ↓HIST1H3F, ↓HIST1H3G, ↓HIST1H3I, ↓HIST1H3J, ↓HIST1H4A, ↓HIST1H4C, ↓HIST1H4D, ↓HIST1H4F, ↓HIST1H4L, ↓HIST2H2BF, ↓KAT8, ↓KIF18B, ↓LBR, ↓MCM2, ↓MCM5, ↓MFAP4, ↓SMU1, ↓UBE2B, ↑USP53	61	35	Cellular Assembly and Organization, DNA Replication, Recombination, and Repair, Post-Translational Modification
2	MOF vs NT gene status OK SB FC2 p05 - 2018-06-11	↓AURKB, ↓BUB1, ↓BUB1B, ↓CDCA3, ↓CDCA5, ↓CDCA8, ↓CENPA, ↓CENPF, ↓CENPH, ↓CENPI, ↓CENPK, ↓CENPM, ↓CENPU, ERK, ↓ESCO2, farnesyl transferase, ↓GAS2L3, ↑IMPACT, ↓KIF2C, ↓KNL1, Lamin b, ↓NCAPG, ↓NCAPG2, ↓NCAPH, ↓NDC80, ↓NUF2, ↓POC1A, ↓SGO1, ↓SGO2, ↓SKA1, ↓SPC24, ↓SPC25, ↓TACC3, ↓TTK, ↓ZWINT	52	32	Cell Cycle, Cellular Assembly and Organization, DNA Replication, Recombination, and Repair
3	MOF vs NT gene status OK SB FC2 p05 - 2018-06-11	↑ARHGAP22, ATM/ATR, ↓C4orf33, ↓CEP128, ↓CNTRL, ↑DDAH2, ↓DDX11, ↓FAIM2, ↓FAM198B, ↓FAM83D, ↑FAM83H, ↓GTSE1, ↓HAUS8, ↓HJURP, ↓HMMR, ↓KIF15, ↑LPXN, ↓MKI67, Pde4, ↓PI4K2B, ↑PLEKHN1, ↓PLK4, ↑PLSCR4, ↓PM20D2, ↓RRM2, ↓SASS6, ↓SP4, ↓SPATA24, ↓STIL, ↓TNRC6B, ↓TPX2, ↓TRIP13, ↑TRPC4, Vegf, ↓WDR62	52	32	Cell Cycle, Cellular Assembly and Organization, DNA Replication, Recombination, and Repair
4	MOF vs NT gene status OK SB FC2 p05 - 2018-06-11	↓ANLN, ↑ARHGAP28, ↓ARHGAP11A, AURK, ↑BTG2, ↓CDC7, ↓CDC45, CDK2-CyclinE, ↓CNOT8, DNA Polymerase, ↑DPYSL4, E2f, ↑E2F7, ↑EDA2R, ↑FDXR, ↓GINS2, ↓GOLGA4, Hat, ↓HIST1H3B, ↓HIST3H2BB, ↓IRAK1BP1, Mcm, ↓MCM6, ↓MCM8, ↓MCM10, ↓MYO1E, NFkB (complex), ↓NUAK2, ↓ORC6, ↑RAB31, ↓RNF112, ↑RRAD, ↓TCEAL7, ↑TNFRSF10C, ↓TTC39B	42	28	Connective Tissue Disorders, Developmental Disorder, Gastrointestinal Disease
5	MOF vs NT gene status OK SB FC2 p05 - 2018-06-11	APC/APC2, Beta Tubulin, ↓CCNF, ↓CDCA2, ↓CENPE, ↓CIT, ↓DEPDC1, ↓DIXDC1, DNA-PK, Dynein, ↓FOXM1, ↓H1F0, ↓HIST1H1A, ↓HIST1H1B, ↓HIST1H1D, Histone H1, Importin alpha, Importin alpha/beta, Importin beta, Jnk, ↓KIF14, ↓KIF22, ↓KIF23, ↓KIF20A, ↓KIF20B, ↓KIF4A, ↓KPNA5, ↓PGM2L1, ↑POM121/POM121C*, ↑PPM1L, ↓PRC1, ↓PTMA, ↓SAPCD2, ↓SLC12A2, ↓TNFAIP8L1	38	26	Cell Cycle, Cellular Assembly and Organization, DNA Replication, Recombination, and Repair
6	MOF vs NT gene status OK SB FC2 p05 - 2018-06-11	20s proteasome, 26s Proteasome, alcohol group acceptor phosphotransferase, Alpha tubulin, ↓AURKA, ↓BIRC5, ↓CEP55, ↓CLSPN, ↑CPNE8, ↑CRIP1, ↓CXCL12, ↑DAPK2, ↓DLGAP5, ↓EXO1, ↑FAM234B, ↓LMNB1, MAP1LC3, ↓MAPK6, Mlc, MutS alpha, ↓MYBL2, ↓NEK2, ↓PIMREG, ↓PLK1, PP2A, ↓PSIP1, ↓RACGAP1, ↑RAI14, Rb, ↓RFC3, ↓SPAG5, ↓TOP2A, ↓TRO, ↓TROAP, ↓TYMS	38	26	Cell Cycle, Cellular Movement, Cancer
7	MOF vs NT gene status OK SB FC2 p05 - 2018-06-11	APC (complex), APC-CDC20, ↓CCNA2, ↓CCNB1, ↓CCNB2, ↓CD200, Cdc2, ↓CDC6, ↓CDC20, Cdc25, ↓CDCA7L, Cdk, ↓CDK1, CDK1-Cyclin B, ↓CDT1, ↓CHEK1, Cyclin A, Cyclin B, ↓FBXO5, ↓GINS1, ↓GMNN, ↓HELLS, ↓MAD2L1, ↓MCM4, ↓MMS22L, Mpf, ↓ORC1, PI3K (complex), ↓PKMYT1, ↑PODXL, ↓PTTG1, RPS6KA, ↓SCML2, ↓UBE2C, Vla-4	31	23	Cell Cycle, Cellular Assembly and Organization, DNA Replication, Recombination, and Repair
8	MOF vs NT gene status OK SB FC2 p05 - 2018-06-11	↑ADH1B, ↓ATP6V1C1, C/ebp, ↑CD276, ↓CEMIP, ↓COL11A1, ↑CXCL8, ↑DLL1, estrogen receptor, ↓EZH2, ↓GMCL1, Hdac, ↓HIST1H2AE, histone deacetylase, Histone h4, ↓KAT5, ↓LMAN1, ↑MMP15, ↓MXD1, N-cor, ↓NEURL1B, Notch, Nr1h, ↓NUSAP1, ↓OIP5, P-TEFb, ↑PCDH19, ↓POLE2, Rxr, Secretase gamma, Serine Protease, ↓SGPL1, ↓SPEG, thymidine kinase, ↓TMPO	29	22	Cancer, Organismal Injury and Abnormalities, Hematological System Development and Function
9	MOF vs NT gene status OK SB FC2 p05 - 2018-06-11	ADRB, ↓ASPM, ↓CDC25A, ↓CDKN3, ↑CDKN1A, ↑CLCA2, cytochrome C, ↓E2F1, ↑FGF2, FSH, Gamma tubulin, ↓GLCE, Hedgehog, HISTONE, Hsp27, Hsp70, Immunoglobulin, ↑KCTD12, Lh, ↓MAGEF1, ↑mir-137, ↓MYOCD, ↑NEFL, PARP, ↑PNP, ↑PTP4A1, ↑PTPRB, ↓SALL2, ↑SERPINB2, ↓SKIL, Sod, SRC (family), ↓SUMO3, Ubiquitin, ↓ZNF365	27	21	Cell Cycle, Hematological System Development and Function, Hematopoiesis
10	MOF vs NT gene status OK SB FC2 p05 - 2018-06-11	↓ALDH1A1, ↓B4GALT6, BRCA1-BRCA2-FANCD2-FANCN-RAD51, ↓BRIP1, Ctbp, elastase, ↑ELF2, ERK1/2, ETS, FA, Fanc, ↓FANCB, ↓FANCG, Fibrin, ↓HHEX, ↑HIPK2, ↓HS2ST1, ↓MYBL1, Pkg, ↓RAD51, ↓RAD51AP1, ↓RASD1, ↓RECQL4, Rfc, ↑RHBDF2, RPA, ↓SLC7A11, Smad1/5/8, Smad2/3, TCF, ↑TCF7, TGFBR, TLR7/8, ↓TMEFF2, ↓UBE2T	24	19	Cardiovascular Disease, Developmental Disorder, Hematological Disease
11	MOF vs NT gene status OK SB FC2 p05 - 2018-06-11	↓ADAMTS3, Alpha Actinin, Alpha catenin, calpain, Collagen type I, Collagen type II, Collagen(s), ↑CYP3A5, ↓DCP2, ↓DHRS13, EGLN, Erm, ↓EZR, ↓F3, F Actin, ↓FEN1, ↑GEMIN5, LDL-cholesterol, ↓LZTFL1, ↓MBP, P glycoprotein, P38 MAPK, ↓PBK, ↓PCLAF, ↓PDGFRL, Rock, ↑SELPLG, ↓SRP9, SYK/ZAP, ↓TAF10, trypsin, UBE2, ↓VLDLR, ↓WDR76, ↑ZMAT3	24	19	Cellular Function and Maintenance, Cellular Movement, Hematological System Development and Function
12	MOF vs NT gene status OK SB FC2 p05 - 2018-06-11	↑ATP5MC1, ↑CAPG, ↑CD68, ↓CDK15, ↓CKAP2L, ↓CKS2, ↓CSTF2T, ↓DAGLA, ↓DENND5B, ↓FAM173B, Filamin, ↓HIST1H2AI, ↓HIST1H2AL, ↓HIST1H2AM, ↓HIST2H3D, ↑IGF1R, ↓KIF20A, ↓MBOAT7, ↓MXD1, ↓NEIL3, ↑NRG1, ↑NUPR1, ↓P3H1, ↓PECR, ↓POMGNT2, ↓PRELID2, ↓RNFT2, ↓SLC25A20, ↑SPTLC2, ↓SPTSSA, ↓STX18, ↓TMEM164, ↓TMEM181, ↓VPS45, ↑ZFP36L2	24	19	Small Molecule Biochemistry, Lipid Metabolism, Cell-To-Cell Signaling and Interaction
13	MOF vs NT gene status OK SB FC2 p05 - 2018-06-11	↓ANKRD46, ↓ARMCX4, ↑C17orf82, ↓C4orf46, ↑CARMIL1, ↓CCDC34, CCDC198, CDC20B, ↓CHSY1, ↓CIT, ↓ELAVL1, FAM90A1, ↓GOLGA1, ↓IMPAD1, ↓INHBE, ↑JOSD1, KRT31, ↓MIGA1, ↓NEK2, ↑PDE4DIP, ↓PHTF2, ↓PON2, ↑RABL2A, ↑RCOR3, ↓SLC18B1, ↑SMG9, TMED7, ↓TMEM59, ↓TMEM106C, ↓TMEM30A, ↓TRAF2, ↑TRIOBP, ↓TROAP, ↓UST, ↓ZBTB43	20	17	Post-Translational Modification, Cell Cycle, Cellular Assembly and Organization
14	MOF vs NT gene status OK SB FC2 p05 - 2018-06-11	↓APP, ↓ARL6IP6, ↑ARMC1, ↑BLOC1S2, ↓C1D, ↑C4orf22, ↓CEND1, ↑CFDP1, ↑DACH2, DNAJC6, ↓DNAJC12, ↑DNAJC21, ↓ECI1, ↓GALNT12, ↓GYG2, ↓HIST1H2BL, ↓HIST1H3H, ↑HSPA8, ↓HSPB7, ↑HTT, ↓IFT80, ↓JPH2, ↑MT-ATP6, ↓NAPB, ↓ODF2L, ↑OSBPL9, ↑OSBPL11, ↓PSRC1, ↓SHISA3, ↑THAP4, TPH1, ↓TRAF3IP1, ↓TRIM16L, ↑TTC23, ↑VSNL1	18	16	Neurological Disease, Organismal Injury and Abnormalities, Cell Death and Survival
15	MOF vs NT gene status OK SB FC2 p05 - 2018-06-11	ADRA2B, ↓B3GALNT1, ↓BTD, ↓C12orf76, ↓CCND1, Cdc25, Cdc25B/C, ↓CDK1, ↓CENPK, ↑CUEDC2, ↓CUL3, ↓DEPDC1B, ↓DONSON, ↑DUSP14, ESR1, ↓FAM103A1, ↓GAS2L3, ↓HIST1H2BE, ↑LIX1L, ↑MAPK8, ↑MDFIC, ↓MTFR2, ↑NXF1, ↓PBK, ↓PHF6, ↑PPM1H, PPP1R1A, RBMXL2, ↑RNF187, RPRM, ↓SLC2A12, ↓SPIN4, ↓TMF1, ↓TTK, ↓ZNF367	18	16	Amino Acid Metabolism, Post-Translational Modification, Small Molecule Biochemistry
16	MOF vs NT gene status OK SB FC2 p05 - 2018-06-11	Akt, ↑CAV2, CDK4/6, CDK4/6-Cyclin D1, ↓CDKN2C, ↑CLCN3, ↓CTH, Cyclin D, Cyclin E, Gcn5l, Hif1, ↑MALL, NADPH oxidase, ↓NDNF, ↓NOX4, p70 S6k, Pdgf (complex), PDGF BB, PDGF-AA, PDGF-DD, ↓PDGFD, ↑PEA15, PLA2, ↓SEMA3B, ↓SEMA6D, ↓SLC1A1, ↑SLC39A10, Smad, Sos, ↓SV2A, TEAD, ↑THBD, transglutaminase, VAV, VitaminD3-VDR-RXR	17	15	Amino Acid Metabolism, Small Molecule Biochemistry, Cardiovascular System Development and Function
17	MOF vs NT gene status OK SB FC2 p05 - 2018-06-11	↑ARHGEF26, c-Src, Caspase 3/7, CD3 group, Cg, Creb, ↑CREB5, ↑CRH, cyclooxygenase, Dlg, Fgf, Fgfr, Filamin, ↑HAS2, ↓HIST1H2AG, ↓HMGN2, ↓IMPA1, Integrin, ↓ITGB8, ↑IVL, Lfa-1, ↓LIN7C, MAP2K1/2, ↓MCAM, Mek, ↓MEST, Pkc(s), PLC gamma, Ppp2c, Raf, ↓RNASEH2A, Rsk, ↑SPRY1, ↓STAR, Wnt	17	15	Endocrine System Development and Function, Lipid Metabolism, Small Molecule Biochemistry
18	MOF vs NT gene status OK SB FC2 p05 - 2018-06-11	↑ACSL4, Alp, Ap1, ↓CADM1, caspase, ↑CCNG1, CD3, ↓CD44, chemokine, ↓CHN1, collagen, Collagen type IV, cytokine, Growth hormone, Gsk3, GTPase, Hsp90, ↓ID2, Ifn gamma, ↓IGF1, ↓IL11, IL12 (family), Insulin, Interferon alpha, Laminin (complex), ↓MAK16, Mmp, MTORC1, ↑PDK4, ↑PIDD1, ↑RPS27L, ↓SEC23A, ↑SH3GLB1, Tgf beta, ↓TGFBR1	17	15	Cell Death and Survival, Embryonic Development, Organ Development
19	MOF vs NT gene status OK SB FC2 p05 - 2018-06-11	↓ACKR3, Actin, ↓ADRA1B, Calmodulin, Clathrin, ↓EDNRB, ↓ESPL1, Focal adhesion kinase, ↓FZD6, G protein, G protein alphai, G-protein beta, ↑GCNT4, GNRH, Gpcr, ↓GPR1, ↓GPR173, ↓GRPR, Histone h3, IKK (complex), ↑KCNQ5, ↓LGR4, ↓LMBRD2, ↓MCHR1, p85 (pik3r), PLC, Rac, ↑RAP2B, Ras homolog, Relaxin, Sfk, STAT, ↓TBXA2R, TCR, tubulin	17	15	Cell Signaling, Cellular Function and Maintenance, Vitamin and Mineral Metabolism
20	MOF vs NT gene status OK SB FC2 p05 - 2018-06-11	↑AHNAK2, ↑ANXA2, ↓ATG4D, ↓CBR3, CYP3A7, CYP4F3, ↓DNAJC27, DNAJC28, ↑EXT1, ↑FDXR, ↑FMO2, ↓GLCE, ↓GNS, HCAR2, ↓HIST1H2BF, HNF4A, HSPA12B, LAD1, ↑LGALS3, ↑MAOA, ↑MAPK1, miR-143-3p (and other miRNAs w/seed GAGAUGA), ↓NUCB2, ↓OSTM1, ↑PIGF, ↑PNPLA3, ↑PODXL, ↓PRIM1, ↑RNF152, ↓SGCE, ↓SLC46A3, SORCS3, ↓STARD10, ↑TGFB1, ↑ZNF300	17	15	Cell-To-Cell Signaling and Interaction, Connective Tissue Development and Function, Skeletal and Muscular System Development and Function
21	MOF vs NT gene status OK SB FC2 p05 - 2018-06-11	14-3-3, ADCY, Angiotensin II receptor type 1, ↑ATP2B4, ↓CACNG7, Calcineurin A, Calcineurin protein(s), ↓CAMK4, CaMKII, ↓CNIH2, ↑CNIH3, Cofilin, Collagen Alpha1, ↓EDN1, ↑GAL, GRIA, ↓GRIA1, ↓IQGAP3, Ldh (complex), MEF2, ↓MEF2A, Nfat (family), NMDA Receptor, Nos, Pka, PP1 protein complex group, ↑PRKAG1, PTPase, ↓RAB39B, Rap1, ↓SHCBP1, Tropomyosin, TSH, voltage-gated calcium channel, ↑XIRP2	15	14	Cardiovascular System Development and Function, Tissue Morphology, Cell-To-Cell Signaling and Interaction
22	MOF vs NT gene status OK SB FC2 p05 - 2018-06-11	↑ARHGAP39, ↓ARHGEF37, ↑BIN3, ↑CCDC6, ↑CYTH3, DLGAP1, DNAJC6, ↑ELFN1, ↑ELP5, ↓ENOSF1, ↑ESRRA, ↓FRAT2, ↑GIPC2, ↑KLHL15, ↑KRTAP1-5, ↑KRTAP2-3/KRTAP2-4, ↓MTMR4, ↑MYPN, ↓OSBPL1A, ↓PKMYT1, ↑PPP1CA, ↓RELL1, ↑RNF34, ↑SACM1L, ↑SACS, ↓SEC63, ↑SLC7A14, TLDC2, ↓TMEM9, ↑TMEM132B, ↑TRIM25, ↓TRIM59, ↓UBXN2A, ↑WDR91, ↑WIPF2	15	14	Cancer, Endocrine System Disorders, Gastrointestinal Disease
23	MOF vs NT gene status OK SB FC2 p05 - 2018-06-11	↓BRCA1, BRCA1-BARD1, BRCA1-BRCA2-FANCD2-FANCN-RAD51, ↓C5orf15, ↓CAND2, Cdsn, Ck2, ↑DCTPP1, ↓DDIAS, ↓DHRS7B, ↓EXD2, ↓FANCD2, ↑FBXL17, FOXJ1, ↑GGN, ↑GMNC, ↓GOLT1B, ↑GPD1L, HP1, ↑MAN2C1, MATN1, ↓MATN2, MATN4, ↓NMRAL1, Proinsulin, Ptprv, RFX4, RNA polymerase II, ↑RTL1, ↓SCG5, SCGN, ↑SGPP1, SRL, STK31, TFIIF	14	13	Cellular Assembly and Organization, DNA Replication, Recombination, and Repair, Cancer
24	MOF vs NT gene status OK SB FC2 p05 - 2018-06-11	↓AFF3, ↓AIFM2, AQP9, ATP6V0D2, ↓BCL2L13, Casein, ↓CREBRF, ↓CRISPLD2, DCSTAMP, ↓FOXO1, HRK, ↑KLF7, LAD1, ↑LCE3D, ↑LMNA, ↓LYSMD3, ↑MALL, ↓MAP4K2, MYCN, ↓NACAD, ↓NEK2, ↓OLFML2B, ↓PBK, ↓PRDM1, ↑SCARA5, SCAVENGER receptor CLASS A, ↓SLC16A7, ↑SLC25A37, ↑SORD, STAB1, ↓SYT11, ↓TM6SF1, TNF, TNFSF11, ↓ZFAND5	14	13	Cellular Development, Cellular Growth and Proliferation, Connective Tissue Development and Function
25	MOF vs NT gene status OK SB FC2 p05 - 2018-06-11	↓ABCA1, ↓ABCA8, ↓AGPAT1, ↑ANAPC13, ↓CANT1, ↓CANX, CD1E, ↑CRIP1, CST7, ↑CTDNEP1, ↑CTNNB1, ↓EPB41L4B, ↓ESCO2, ↓GALC, ↓H1F0, H3F3C, IL4, hl, ↓KDM1A, ↓KIF11, LRP1B, ↑MOV10, ↑MVK, ↓NCEH1, ↑NR3C1, ↓PCID2, ↓PHLDA2, ↑PMP22, ↓PRR11, SLC1A6, ↓SLC1A7, ↑SPTLC1, ↓TEDC2, ↑TMTC1, ↓ZNF420	14	13	Cancer, Endocrine System Disorders, Organismal Injury and Abnormalities

Figure S5. RNA-Seq data for related network changes in IPF fibroblasts transfected with siRNA Mof vs NT.

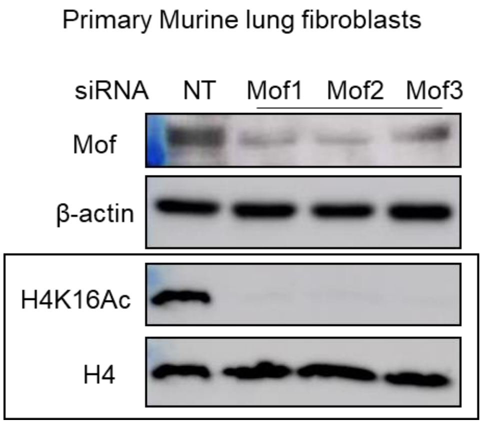

Figure S6. Testing of three different siRNA mMof for knocking down Mof in primary murine lung fibroblasts. Western blots showing Mof in whole cell lysate, or H4K16Ac in histone extracts by western blots. The loading control is β-actin for whole cell lysate, or H4 for histone extracts. Mof2 was used in the animal studies; sequence of siRNA mMof is in Table 2S.

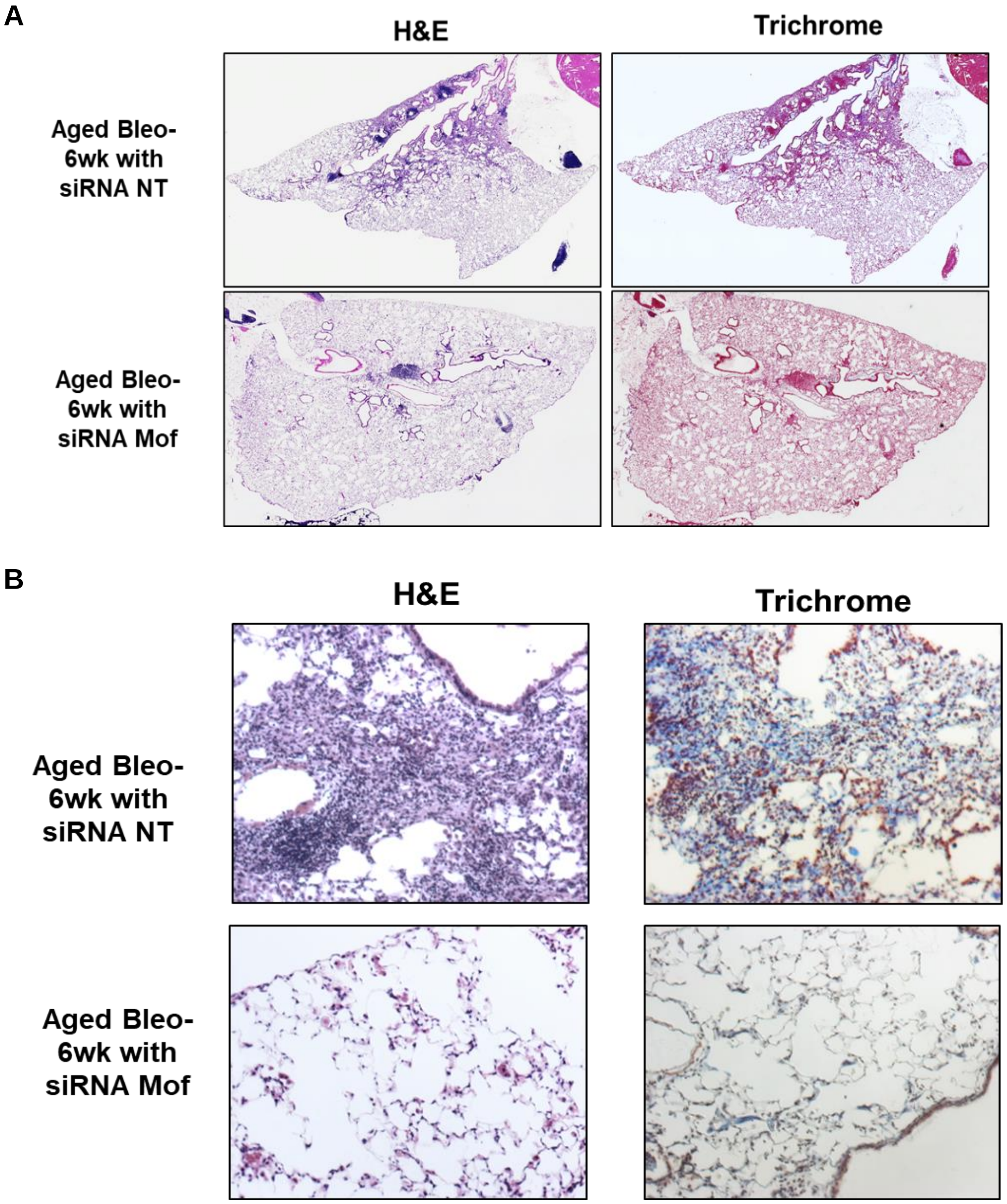

Figure S7. 18-month old mice were subjected to bleomycin injury, then treated with siRNA NT or Mof by nasal from 3-6week after bleomycin injury. Slides are stained with H&E or with trichrome. Pictures were taken at 4 x **(A)**, or at 20 x **(B)**.

Table S1. Information of the de-identified subjects of the non-IPF or IPF samples used in this study.

Non-IPF # 1	Non-IPF # 2	Non-IPF # 3	Non-IPF # 4	IPF-a	IPF-b	IPF-c	IPF-d	IPF-e	IPF-f
48yr, male	20yr, female	32yr, white, male	61yr, white, female	64yr, male	60yr, male	56yr, white, male	48yr, black, male	54yr, white, male	61yr, white, female

Table S2. siRNA sequences and Primers sequences for PCR

Name		Sequence
Mof (human) for cell culture (from Thermo Scientific, cat # J-014800-12)	siRNA	5'-ACUUUGACGUGGAGCCGUU-3'
Non-targeting (NT) (for cells culture)	siRNA	Sense: 5'-UAAGGCUAUGAAGAGAUACUU-3',<br>Anti-sense: 5'-GUAUCUCUUCAUAGCCUUAUU-3'.
mMof<br>(mouse, for *in vivo*)	siRNA	5'-UGAGGUGUUCCUCUACCAGCUUAGG -3'
NT Negative Control (used in vivo, mouse)	siRNA	5'-AGCUACACUAUCGAGCAAUUAACUU-3'
Mof (human)<br>(ENSG00000103510)	RT-PCR	F: 5'-AATGGCACAGCTGGGACTAGAACT-3'<br>R: 5'-GCTTGGCTATAGCAACTGCCGAAT-3'
a-SMA<br>(human)	RT-PCR	F: 5'-ATGGCTCTGGGCTCTGTAA-3'<br>R: 5'-GGAACCTAATCTGTGTCCTGTTATG-3'
	ChIP-PCR	Set A<br>F: 5'- CTTTCTTCTTTGCATGCTACCG<br>R: 5'- GCTGGAATTTCAGGCCATTTC<br>Set B<br>F: 5'-GAGGTCCCTATATGGTTGTGTTAG-3'<br>R: 5'-AGCTGAAAGCTGAAGGGTTAT-3'
Nox4 (human)<br>(ENSG00000086991)	RT-PCR	F: 5'- AGATGTTGGGGCTAGGATTG-3'<br>R: 5'- TCTCCTGCTTGGAACCTTCT-3'
	ChIP-PCR	Set A<br>F: 5'-ATCTCCTGACTCCGTGATCC-3'<br>R: 5'-GCGTGTTAGCACTCTCTCACTTTA-3'<br>Set B<br>F: 5'-GAACAGCAGCAGCCACAAC-3'<br>R: 5'-CTACCCAGAGCCGGTTTTC-3'
Col1A1 (human)<br>(ENSG00000108821)	RT-PCR	F: 5'-TCGAGGGCCAAGACGAAGAC-3'<br>R: 5'- CGCACAACACCTTGCCGTTG-3'
	ChIP-PCR	Set A:<br>F: 5'-CTCTCCATTCCAACTCCCAAA-3'<br>R: 5'-ATGGAGAGCAGGGAGGAA-3'<br>Set B:<br>F: 5'- CGTGAGTTGGTGCAAGAGAGAA-3'<br>R: 5'-GGCCTTCCTGATTGCTTCTACA-3'
Survivin (human) (ENSG000000089685)	RT-PCR	F: 5'- AGCCCTTTCTCAAGGACCAC-3'<br>R: 5'- CAGCTCCTTGAAGCAGAAGAA-3'

	ChIP-PCR	Set A:<br>F: 5'- TCACTTGAGGTCAGGAGTTTG-3'<br>R: 5'- CCCGAGTAGCTGAGATTAAAGG-3'<br>Set B:<br>F: 5'- ACCACGCCCAGCTAATTT -3'<br>R: 5'- CATCACTTGAGTCCTGGAGTTC -3'
β-actin (human)	RT-PCR	F: 5'-TGCTATCCAGGCTGTGCTAT-3'<br>R: 5'- AGTCCATCACGATGCCAG T-3'

Table S3. Excel file for the gene expression in siRNA Mof vs NT cells.
