# Supplementary material for: Modulation of H4K16Ac levels reduces pro-fibrotic gene expression and mitigates lung fibrosis in aged mice
Source: Theranostics. 2022 Jan 1;12(2):530–41. doi: 10.7150/thno.62760 (PMC8692895; doi:10.7150/thno.62760)
Supplement: Supplementary file 2 — Supplementary table 3. [file thnov12p0530s2.pdf]

Table 3S. Excel file for the gene expression in siRNA Mof vs NT cells.

| test_id | gene_id | gene | locus | sample_1 | sample_2 | status | NT FPKM | MOF FPKM | MOF vs. NT fold change | log2(fold_change) | test_stat | p_value | q_value | significant |
|---|---|---|---|---|---|---|---|---|---|---|---|---|---|---|
| XLOC_001667 | XLOC_001667 | MIR137,MIR137H | chr1:98453555-98515249 | NT | MOF | OK | 0.53098 | 35.6781 | 67.192926 | 6.07024 | 2.94508 | 5.00E-05 | 0.00368902 | yes |
| XLOC_000719 | XLOC_000719 | IVL | chr1:152881038-152884362 | NT | MOF | OK | 0.143068 | 1.664 | 11.630833 | 3.53988 | 2.85378 | 0.002 | 0.0614213 | no |
| XLOC_016013 | XLOC_016013 | TMEM14E | chr3:151980404-152183569 | NT | MOF | OK | 0.309345 | 2.24832 | 7.2680017 | 2.86156 | 0.246694 | 0.2391 | 0.842227 | no |
| XLOC_021929 | XLOC_021929 | SCARA5 | chr8:27727398-27850369 | NT | MOF | OK | 0.177836 | 1.01556 | 5.7106548 | 2.51366 | 2.28908 | 0.002 | 0.0614213 | no |
| XLOC_012489 | XLOC_012489 | XIRP2 | chr2:167744996-168116261 | NT | MOF | OK | 0.160818 | 0.885562 | 5.50661 | 2.46117 | 1.47467 | 0.01205 | 0.194666 | no |
| XLOC_001835 | XLOC_001835 | LCE3D | chr1:152551859-152552980 | NT | MOF | OK | 0.713907 | 3.10838 | 4.3540405 | 2.12236 | 1.61189 | 0.0385 | 0.376597 | no |
| XLOC_000989 | XLOC_000989 | LINC00260 | chr1:203595914-203713209 | NT | MOF | OK | 0.348741 | 1.50065 | 4.3030501 | 2.10536 | 0.132585 | 0.1677 | 0.756137 | no |
| XLOC_016112 | XLOC_016112 | GMNC | chr3:190570525-190580465 | NT | MOF | OK | 0.278838 | 1.18929 | 4.2651647 | 2.0926 | 2.14754 | 0.0018 | 0.0568668 | no |
| XLOC_007497 | XLOC_007497 | CSNK1A1P1 | chr15:36871811-37110707 | NT | MOF | OK | 0.209796 | 0.886098 | 4.2236172 | 2.07848 | 0.65062 | 0.32815 | 0.915657 | no |
| XLOC_023979 | XLOC_023979 | PCDH19 | chrX:99546641-99665271 | NT | MOF | OK | 0.258953 | 1.08682 | 4.1969778 | 2.06936 | 2.34881 | 0.0002 | 0.0112558 | yes |
| XLOC_021191 | XLOC_021191 | CYP3A5 | chr7:99245812-99277621 | NT | MOF | OK | 0.370165 | 1.54183 | 4.1652506 | 2.05841 | 1.54029 | 0.00125 | 0.0434626 | yes |
| XLOC_004579 | XLOC_004579 | BLID | chr11:121959810-122073770 | NT | MOF | OK | 0.414574 | 1.7153 | 4.1375002 | 2.04876 | 0.361647 | 0.3933 | 0.952607 | no |
| XLOC_016377 | XLOC_016377 | AREG | chr4:75310852-75320726 | NT | MOF | OK | 0.216185 | 0.839289 | 3.8822721 | 1.9569 | 1.28959 | 0.05775 | 0.473425 | no |
| XLOC_015988 | XLOC_015988 | PLSCR4 | chr3:145910123-145968966 | NT | MOF | OK | 9.72991 | 34.8272 | 3.5793959 | 1.83972 | 3.85904 | 5.00E-05 | 0.00368902 | yes |
| XLOC_023511 | XLOC_023511 | DACH2 | chrX:85403454-86087605 | NT | MOF | OK | 0.22939 | 0.795579 | 3.4682375 | 1.79421 | 1.32813 | 0.0058 | 0.123774 | no |
| XLOC_002528 | XLOC_002528 | C10orf11 | chr10:77542518-78317126 | NT | MOF | OK | 0.208893 | 0.719594 | 3.4447971 | 1.78442 | 1.01901 | 0.10075 | 0.611372 | no |
| XLOC_002763 | XLOC_002763 | DPYSL4 | chr10:134000413-134019280 | NT | MOF | OK | 2.5387 | 8.60505 | 3.3895498 | 1.76109 | 2.76342 | 5.00E-05 | 0.00368902 | yes |
| XLOC_015294 | XLOC_015294 | LOC100129550 | chr3:122605359-122611263 | NT | MOF | OK | 0.349275 | 1.1706 | 3.3515139 | 1.74481 | 2.21086 | 0.00085 | 0.0324448 | yes |
| XLOC_000487 | XLOC_000487 | CLCA2 | chr1:86889768-86922240 | NT | MOF | OK | 0.542981 | 1.80917 | 3.3319214 | 1.73635 | 2.08391 | 0.0014 | 0.0470556 | yes |
| XLOC_016172 | XLOC_016172 | SCARNA22 | chr4:1873122-1983934 | NT | MOF | OK | 24.1979 | 80.5439 | 3.3285492 | 1.7349 | 5.32008 | 0.5511 | 0.999021 | no |
| XLOC_005413 | XLOC_005413 | MIR1291,SNORA | chr12:49046994-49076035 | NT | MOF | OK | 30.0717 | 98.7973 | 3.2853912 | 1.71607 | 5.14652 | 0.4591 | 0.980057 | no |
| XLOC_007370 | XLOC_007370 | LOC100506874 | chr15:85070426-85123412 | NT | MOF | OK | 0.225433 | 0.738053 | 3.273935 | 1.71103 | 1.00373 | 0.1117 | 0.637834 | no |
| XLOC_006664 | XLOC_006664 | CRIP1 | chr14:105953256-105955124 | NT | MOF | OK | 0.949482 | 3.06672 | 3.2298875 | 1.69149 | 1.20635 | 0.04905 | 0.434484 | no |
| XLOC_007618 | XLOC_007618 | DAPK2 | chr15:64199234-64338521 | NT | MOF | OK | 0.356299 | 1.1486 | 3.223697 | 1.68871 | 1.67538 | 0.0093 | 0.167207 | no |
| XLOC_003230 | XLOC_003230 | SNORA52 | chr11:809935-812876 | NT | MOF | OK | 27.5148 | 86.3678 | 3.1389579 | 1.65028 | 1.68953 | 0.53075 | 0.999021 | no |
| XLOC_009696 | XLOC_009696 | KRTAP2-3 | chr17:39215492-39216344 | NT | MOF | OK | 6.36993 | 19.957 | 3.1330015 | 1.64754 | 2.47457 | 5.00E-05 | 0.00368902 | yes |
| XLOC_003693 | XLOC_003693 | FOLR3 | chr11:71846770-71850934 | NT | MOF | OK | 0.386072 | 1.19586 | 3.0975051 | 1.6311 | 1.1729 | 0.05055 | 0.442907 | no |
| XLOC_021916 | XLOC_021916 | NEFL | chr8:24808468-24814383 | NT | MOF | OK | 2.46186 | 7.55268 | 3.0678755 | 1.61724 | 2.87604 | 5.00E-05 | 0.00368902 | yes |
| XLOC_010685 | XLOC_010685 | SNORA68 | chr19:17970686-17974133 | NT | MOF | OK | 22.1234 | 67.312 | 3.0425703 | 1.60529 | 2.00641 | 0.55055 | 0.999021 | no |
| XLOC_000878 | XLOC_000878 | FMO2 | chr1:171154387-171181822 | NT | MOF | OK | 3.56279 | 10.7944 | 3.0297604 | 1.59921 | 3.00839 | 5.00E-05 | 0.00368902 | yes |
| XLOC_016055 | XLOC_016055 | SLC7A14 | chr3:170177341-170303863 | NT | MOF | OK | 0.567328 | 1.70219 | 3.0003631 | 1.58514 | 2.47395 | 5.00E-05 | 0.00368902 | yes |
| XLOC_023473 | XLOC_023473 | ITGB1BP2 | chrX:70430034-70948962 | NT | MOF | OK | 0.241486 | 0.720321 | 2.9828686 | 1.5767 | 0.109715 | 0.4304 | 0.96699 | no |
| XLOC_006068 | XLOC_006068 | DCLK1 | chr13:35516423-36705514 | NT | MOF | OK | 0.425784 | 1.26779 | 2.9775426 | 1.57412 | 0.818075 | 0.3081 | 0.903396 | no |
| XLOC_021321 | XLOC_021321 | PODXL | chr7:131185020-131241376 | NT | MOF | OK | 2.52374 | 7.51449 | 2.9775215 | 1.57411 | 2.19578 | 5.00E-05 | 0.00368902 | yes |
| XLOC_000473 | XLOC_000473 | GIPC2 | chr1:78511588-78603112 | NT | MOF | OK | 0.252926 | 0.750597 | 2.9676546 | 1.56933 | 1.49406 | 0.01785 | 0.245846 | no |
| XLOC_017318 | XLOC_017318 | TCF7 | chr5:133450401-133483920 | NT | MOF | OK | 2.27505 | 6.67351 | 2.9333465 | 1.55255 | 2.27628 | 0.0001 | 0.00661202 | yes |
| XLOC_022017 | XLOC_022017 | CRH | chr8:67088611-67090846 | NT | MOF | OK | 0.396196 | 1.14792 | 2.8973538 | 1.53474 | 1.22417 | 0.04105 | 0.391275 | no |
| XLOC_002022 | XLOC_002022 | TNFSF18 | chr1:173010359-173020103 | NT | MOF | OK | 0.775945 | 2.21861 | 2.8592362 | 1.51563 | 1.12232 | 0.06145 | 0.48789 | no |

| | | | | | | | | | | | | | |
|---|---|---|---|---|---|---|---|---|---|---|---|---|---|
| XLOC_012457 | XLOC_012457 | ZEB2-AS1 | chr2:145141941-145278465 | NT | MOF | OK | 0.331001 | 0.93651 | 2.8293268 | 1.50046 | 0.106229 | 0.4682 | 0.983699 | no |
| XLOC_000712 | XLOC_000712 | LCE1F | chr1:152748847-152749445 | NT | MOF | OK | 0.358684 | 1.0073 | 2.8083215 | 1.4897 | 0.905609 | 0.1576 | 0.737417 | no |
| XLOC_018766 | XLOC_018766 | DDAH2 | chr6:31694816-31698039 | NT | MOF | OK | 0.417793 | 1.16717 | 2.7936562 | 1.48216 | 1.22921 | 0.044 | 0.407345 | no |
| XLOC_014117 | XLOC_014117 | LINC00161 | chr21:29911639-29912677 | NT | MOF | OK | 0.794518 | 2.21173 | 2.7837381 | 1.47702 | 1.22041 | 0.0246 | 0.29677 | no |
| XLOC_013965 | XLOC_013965 | SNORA71D | chr20:37049238-37064018 | NT | MOF | OK | 22.7597 | 63.3258 | 2.7823653 | 1.47631 | 0.484435 | 0.56155 | 0.999021 | no |
| XLOC_010231 | XLOC_010231 | SERPINB2 | chr18:61554938-61571124 | NT | MOF | OK | 23.4199 | 64.9992 | 2.7753833 | 1.47269 | 2.63064 | 5.00E-05 | 0.00368902 | yes |
| XLOC_016401 | XLOC_016401 | C4orf22 | chr4:81256873-81884910 | NT | MOF | OK | 0.738996 | 2.03323 | 2.751341 | 1.46014 | 1.20105 | 0.0278 | 0.319146 | no |
| XLOC_022759 | XLOC_022759 | IFNE | chr9:21454266-21559697 | NT | MOF | OK | 0.505497 | 1.37158 | 2.7133297 | 1.44006 | 0.496341 | 0.5845 | 0.999021 | no |
| XLOC_007614 | XLOC_007614 | RPS27L | chr15:63445538-63449741 | NT | MOF | OK | 40.8616 | 110.05 | 2.6932377 | 1.42934 | 3.04499 | 5.00E-05 | 0.00368902 | yes |
| XLOC_002978 | XLOC_002978 | UNC5B-AS1 | chr10:72972291-73062635 | NT | MOF | OK | 0.447087 | 1.18223 | 2.6442952 | 1.40289 | 0.196551 | 0.4566 | 0.979004 | no |
| XLOC_015794 | XLOC_015794 | CADPS | chr3:62384020-62861064 | NT | MOF | OK | 0.681062 | 1.80039 | 2.6435038 | 1.40245 | 1.77886 | 0.00305 | 0.080932 | no |
| XLOC_015405 | XLOC_015405 | RAP2B | chr3:152880028-152886263 | NT | MOF | OK | 7.82161 | 20.6184 | 2.6360813 | 1.3984 | 3.07472 | 5.00E-05 | 0.00368902 | yes |
| XLOC_015604 | XLOC_015604 | ZNF385D | chr3:21462489-21792816 | NT | MOF | OK | 3.34281 | 8.68663 | 2.5986012 | 1.37774 | 2.05986 | 0.00085 | 0.0324448 | yes |
| XLOC_006624 | XLOC_006624 | MEG9 | chr14:101536247-101539273 | NT | MOF | OK | 0.87218 | 2.24345 | 2.5722328 | 1.36302 | 1.62857 | 0.0076 | 0.146667 | no |
| XLOC_006847 | XLOC_006847 | SGPP1 | chr14:64150934-64194756 | NT | MOF | OK | 4.44514 | 11.3237 | 2.5474338 | 1.34905 | 2.52225 | 5.00E-05 | 0.00368902 | yes |
| XLOC_019200 | XLOC_019200 | DLL1 | chr6:170591293-170599697 | NT | MOF | OK | 0.576755 | 1.45417 | 2.5212959 | 1.33417 | 1.49738 | 0.0094 | 0.167907 | no |
| XLOC_016370 | XLOC_016370 | IL8 | chr4:74606222-74609433 | NT | MOF | OK | 3.33444 | 8.39583 | 2.5179131 | 1.33223 | 1.9541 | 0.00165 | 0.0535255 | no |
| XLOC_007126 | XLOC_007126 | ATPBD4-AS1 | chr15:35663169-36151202 | NT | MOF | OK | 0.503111 | 1.25768 | 2.4998062 | 1.32181 | 0.839268 | 0.1684 | 0.757374 | no |
| XLOC_012542 | XLOC_012542 | PPP1R1C | chr2:182818967-182996109 | NT | MOF | OK | 0.449916 | 1.11458 | 2.4773069 | 1.30878 | 1.00229 | 0.0584 | 0.475532 | no |
| XLOC_019128 | XLOC_019128 | STXBP5-AS1 | chr6:147162524-147711612 | NT | MOF | OK | 0.770983 | 1.90608 | 2.4722724 | 1.30583 | 0.468142 | 0.49695 | 0.990434 | no |
| XLOC_018363 | XLOC_018363 | PTP4A1 | chr6:64281919-64293489 | NT | MOF | OK | 28.0247 | 69.1551 | 2.4676482 | 1.30314 | 2.94418 | 5.00E-05 | 0.00368902 | yes |
| XLOC_013839 | XLOC_013839 | RASSF2 | chr20:4760668-4804291 | NT | MOF | OK | 2.74104 | 6.75686 | 2.4650717 | 1.30163 | 2.39312 | 5.00E-05 | 0.00368902 | yes |
| XLOC_020530 | XLOC_020530 | POM121 | chr7:72349935-72425302 | NT | MOF | OK | 5.44454 | 13.3345 | 2.4491509 | 1.29228 | 2.65015 | 5.00E-05 | 0.00368902 | yes |
| XLOC_022154 | XLOC_022154 | HAS2 | chr8:122625270-122657564 | NT | MOF | OK | 51.7304 | 126.133 | 2.4382761 | 1.28586 | 2.29839 | 0.0001 | 0.00661202 | yes |
| XLOC_011445 | XLOC_011445 | ZNF442 | chr19:12460184-12476475 | NT | MOF | OK | 0.33799 | 0.823752 | 2.4372082 | 1.28523 | 1.2978 | 0.02465 | 0.296781 | no |
| XLOC_013964 | XLOC_013964 | SNORA71C | chr20:37049238-37064018 | NT | MOF | OK | 16.9045 | 41.1583 | 2.4347541 | 1.28378 | 0.365145 | 0.5711 | 0.999021 | no |
| XLOC_018269 | XLOC_018269 | PI16 | chr6:36916038-36932613 | NT | MOF | OK | 9.77095 | 23.6696 | 2.4224461 | 1.27646 | 1.46743 | 0.17975 | 0.773737 | no |
| XLOC_015428 | XLOC_015428 | PPM1L | chr3:160473995-160788817 | NT | MOF | OK | 0.401134 | 0.967698 | 2.4124058 | 1.27047 | 1.36669 | 0.0218 | 0.276789 | no |
| XLOC_008148 | XLOC_008148 | MMP15 | chr16:58059281-58080804 | NT | MOF | OK | 1.08631 | 2.61954 | 2.4114111 | 1.26988 | 1.8692 | 0.0021 | 0.063525 | no |
| XLOC_013881 | XLOC_013881 | THBD | chr20:23026269-23030301 | NT | MOF | OK | 3.67876 | 8.8368 | 2.4021138 | 1.2643 | 1.76004 | 0.00195 | 0.0601913 | no |
| XLOC_023804 | XLOC_023804 | KLHL15 | chrX:24001832-24045303 | NT | MOF | OK | 1.38158 | 3.29545 | 2.3852763 | 1.25416 | 2.18599 | 0.00035 | 0.017008 | yes |
| XLOC_021118 | XLOC_021118 | POM121C | chr7:75039623-75115568 | NT | MOF | OK | 4.85044 | 11.4961 | 2.3701149 | 1.24495 | 2.56438 | 0.0001 | 0.00661202 | yes |
| XLOC_018374 | XLOC_018374 | KCNQ5 | chr6:73331570-73908573 | NT | MOF | OK | 1.99769 | 4.72923 | 2.3673493 | 1.24327 | 2.28124 | 5.00E-05 | 0.00368902 | yes |
| XLOC_010388 | XLOC_010388 | RNF152 | chr18:59482303-59560304 | NT | MOF | OK | 9.86665 | 23.3393 | 2.3654736 | 1.24213 | 2.44483 | 5.00E-05 | 0.00368902 | yes |
| XLOC_002092 | XLOC_002092 | LOC100131234 | chr1:198777131-198906558 | NT | MOF | OK | 0.414962 | 0.979527 | 2.3605222 | 1.23911 | 0.795272 | 0.38205 | 0.946768 | no |
| XLOC_014607 | XLOC_014607 | PNPLA3 | chr22:44319618-44343448 | NT | MOF | OK | 0.762722 | 1.79667 | 2.3556027 | 1.2361 | 1.41562 | 0.0212 | 0.272025 | no |
| XLOC_004081 | XLOC_004081 | RRAS2 | chr11:14299465-14386052 | NT | MOF | OK | 49.5057 | 115.99 | 2.3429625 | 1.22833 | 2.55916 | 5.00E-05 | 0.00368902 | yes |
| XLOC_021729 | XLOC_021729 | HAS2-AS1 | chr8:122625270-122657564 | NT | MOF | OK | 5.16758 | 12.0504 | 2.3319233 | 1.22152 | 0.554468 | 0.4526 | 0.975824 | no |
| XLOC_000984 | XLOC_000984 | BTG2 | chr1:203274663-203278729 | NT | MOF | OK | 12.3494 | 28.772 | 2.3298298 | 1.22023 | 2.54424 | 5.00E-05 | 0.00368902 | yes |
| XLOC_005677 | XLOC_005677 | SELPLG | chr12:109015679-109027670 | NT | MOF | OK | 1.65956 | 3.86498 | 2.3289185 | 1.21966 | 1.72836 | 0.00295 | 0.0793222 | no |
| XLOC_023919 | XLOC_023919 | EDA2R | chrX:65815481-65859140 | NT | MOF | OK | 4.32319 | 10.0443 | 2.3233538 | 1.2162 | 2.26422 | 0.00015 | 0.00902985 | yes |
| XLOC_005262 | XLOC_005262 | SLC2A3 | chr12:8071823-8088892 | NT | MOF | OK | 16.8439 | 39.1289 | 2.3230309 | 1.21601 | 2.70521 | 5.00E-05 | 0.00368902 | yes |

| | | | | | | | | | | | | | | |
|---|---|---|---|---|---|---|---|---|---|---|---|---|---|---|
| XLOC_008611 | XLOC_008611 | RRAD | chr16:66955581-66959439 | NT | MOF | OK | 7.24577 | 16.6508 | 2.2980028 | 1.20038 | 2.05395 | 0.00095 | 0.0354784 | yes |
| XLOC_005595 | XLOC_005595 | E2F7 | chr12:77415025-77459360 | NT | MOF | OK | 9.97418 | 22.8133 | 2.2872356 | 1.19361 | 2.59756 | 5.00E-05 | 0.00368902 | yes |
| XLOC_012394 | XLOC_012394 | RABL2A | chr2:114384816-114400975 | NT | MOF | OK | 0.735655 | 1.66442 | 2.2625008 | 1.17792 | 1.25696 | 0.028 | 0.319924 | no |
| XLOC_003035 | XLOC_003035 | FAS-AS1 | chr10:90694830-90775542 | NT | MOF | OK | 0.506797 | 1.14218 | 2.2537229 | 1.17231 | 0.101232 | 0.45155 | 0.975622 | no |
| XLOC_021171 | XLOC_021171 | PDK4 | chr7:95212808-95225925 | NT | MOF | OK | 0.746705 | 1.67933 | 2.2489872 | 1.16928 | 1.35392 | 0.0223 | 0.280197 | no |
| XLOC_016890 | XLOC_016890 | PITX2 | chr4:111538579-111563279 | NT | MOF | OK | 0.717085 | 1.6126 | 2.2488268 | 1.16917 | 0.753522 | 0.3455 | 0.925925 | no |
| XLOC_009726 | XLOC_009726 | KRT17 | chr17:39775691-39780882 | NT | MOF | OK | 0.458352 | 1.02606 | 2.2385852 | 1.16259 | 0.991876 | 0.089 | 0.577603 | no |
| XLOC_003670 | XLOC_003670 | GAL | chr11:68451982-68458643 | NT | MOF | OK | 21.8847 | 48.8849 | 2.2337478 | 1.15947 | 2.16698 | 0.0006 | 0.0251211 | yes |
| XLOC_010114 | XLOC_010114 | RAB31 | chr18:9708227-9862553 | NT | MOF | OK | 7.8916 | 17.6147 | 2.2320822 | 1.15839 | 2.33421 | 0.0001 | 0.00661202 | yes |
| XLOC_005177 | XLOC_005177 | TMEM132B | chr12:125811161-126143589 | NT | MOF | OK | 0.377678 | 0.841353 | 2.2276993 | 1.15556 | 1.46385 | 0.0152 | 0.223475 | no |
| XLOC_023348 | XLOC_023348 | MAOA | chrX:43514154-43606071 | NT | MOF | OK | 3.55926 | 7.89633 | 2.2185314 | 1.14961 | 2.19037 | 0.00015 | 0.00902985 | yes |
| XLOC_003544 | XLOC_003544 | RPLP0P2 | chr11:61382507-61406921 | NT | MOF | OK | 0.477287 | 1.05869 | 2.2181413 | 1.14936 | 1.26467 | 0.0345 | 0.355882 | no |
| XLOC_016571 | XLOC_016571 | CLCN3 | chr4:170541671-170644338 | NT | MOF | OK | 9.00351 | 19.9281 | 2.2133701 | 1.14624 | 2.5103 | 0.0001 | 0.00661202 | yes |
| XLOC_006972 | XLOC_006972 | RTL1 | chr14:101346991-101351184 | NT | MOF | OK | 1.11909 | 2.47501 | 2.2116273 | 1.14511 | 1.61999 | 0.0064 | 0.131254 | no |
| XLOC_020413 | XLOC_020413 | CREB5 | chr7:28338939-28865511 | NT | MOF | OK | 1.43338 | 3.16466 | 2.2078304 | 1.14262 | 2.02883 | 0.0006 | 0.0251211 | yes |
| XLOC_009988 | XLOC_009988 | RHBDF2 | chr17:74466974-74497509 | NT | MOF | OK | 1.28725 | 2.83866 | 2.2052127 | 1.14092 | 1.44777 | 0.0217 | 0.276681 | no |
| XLOC_023717 | XLOC_023717 | SNORA70 | chrX:153626570-153650063 | NT | MOF | OK | 477.538 | 1052.74 | 2.2045157 | 1.14046 | 0.148172 | 0.5013 | 0.991492 | no |
| XLOC_000008 | XLOC_000008 | PLEKHN1 | chr1:901876-910484 | NT | MOF | OK | 0.935538 | 2.0597 | 2.2016209 | 1.13857 | 1.35393 | 0.03045 | 0.334039 | no |
| XLOC_018516 | XLOC_018516 | TNFAIP3 | chr6:138144806-138204451 | NT | MOF | OK | 3.6905 | 8.12207 | 2.2008048 | 1.13803 | 2.17675 | 0.0002 | 0.0112558 | yes |
| XLOC_007675 | XLOC_007675 | C15orf59 | chr15:74032140-74043816 | NT | MOF | OK | 0.509684 | 1.12157 | 2.2005203 | 1.13785 | 0.94708 | 0.11295 | 0.64134 | no |
| XLOC_014815 | XLOC_014815 | BAIAP2L2 | chr22:38480895-38506676 | NT | MOF | OK | 0.614607 | 1.35148 | 2.1989336 | 1.1368 | 1.18724 | 0.041 | 0.391275 | no |
| XLOC_008621 | XLOC_008621 | TPPP3 | chr16:67423711-67427421 | NT | MOF | OK | 0.598788 | 1.31187 | 2.1908756 | 1.1315 | 0.910738 | 0.1043 | 0.619858 | no |
| XLOC_010151 | XLOC_010151 | IMPACT | chr18:22006608-22033494 | NT | MOF | OK | 4.52331 | 9.90496 | 2.1897593 | 1.13077 | 2.16858 | 0.0004 | 0.0189062 | yes |
| XLOC_014996 | XLOC_014996 | GPD1L | chr3:32148002-32210207 | NT | MOF | OK | 1.38121 | 3.01858 | 2.1854606 | 1.12794 | 1.71774 | 0.0032 | 0.0829122 | no |
| XLOC_021343 | XLOC_021343 | HIPK2 | chr7:139246315-139477693 | NT | MOF | OK | 12.3007 | 26.8273 | 2.1809572 | 1.12495 | 2.48352 | 5.00E-05 | 0.00368902 | yes |
| XLOC_020704 | XLOC_020704 | CAV2 | chr7:116139654-116148595 | NT | MOF | OK | 54.5088 | 118.707 | 2.1777585 | 1.12284 | 2.47832 | 5.00E-05 | 0.00368902 | yes |
| XLOC_017758 | XLOC_017758 | FBXL17 | chr5:107194733-107717799 | NT | MOF | OK | 3.52388 | 7.65444 | 2.1721625 | 1.11913 | 2.20025 | 0.0004 | 0.0189063 | yes |
| XLOC_004225 | XLOC_004225 | LPXN | chr11:58294343-58345639 | NT | MOF | OK | 13.5101 | 29.3333 | 2.1712126 | 1.1185 | 2.2363 | 5.00E-05 | 0.00368902 | yes |
| XLOC_002922 | XLOC_002922 | ARHGAP22 | chr10:49654067-49864310 | NT | MOF | OK | 11.9578 | 25.8813 | 2.1643864 | 1.11396 | 2.42577 | 5.00E-05 | 0.00368902 | yes |
| XLOC_009231 | XLOC_009231 | SNORA76 | chr17:62223698-62223831 | NT | MOF | OK | 29.8791 | 64.6506 | 2.1637399 | 1.11352 | 10.7905 | 0.4175 | 0.958864 | no |
| XLOC_023277 | XLOC_023277 | TCEANC | chrX:13671306-13682247 | NT | MOF | OK | 0.35687 | 0.770748 | 2.1597444 | 1.11086 | 1.00914 | 0.1001 | 0.608954 | no |
| XLOC_000987 | XLOC_000987 | ATP2B4 | chr1:203595914-203713209 | NT | MOF | OK | 61.8092 | 133.364 | 2.1576723 | 1.10948 | 2.20126 | 0.0002 | 0.0112558 | yes |
| XLOC_005846 | XLOC_005846 | STARD13-AS | chr13:33677271-34250932 | NT | MOF | OK | 0.449546 | 0.968387 | 2.1541444 | 1.10712 | 0.135906 | 0.5681 | 0.999021 | no |
| XLOC_016855 | XLOC_016855 | ADH1B | chr4:100227526-100242572 | NT | MOF | OK | 27.4371 | 58.9147 | 2.1472641 | 1.1025 | 2.46136 | 5.00E-05 | 0.00368902 | yes |
| XLOC_010212 | XLOC_010212 | LOC100505549 | chr18:55297533-55470327 | NT | MOF | OK | 0.441759 | 0.948016 | 2.1460027 | 1.10165 | 0.134656 | 0.4744 | 0.985274 | no |
| XLOC_005564 | XLOC_005564 | RPSAP52 | chr12:66151799-66360071 | NT | MOF | OK | 1.18035 | 2.52557 | 2.1396789 | 1.09739 | 0.274281 | 0.65855 | 0.999021 | no |
| XLOC_017696 | XLOC_017696 | GCNT4 | chr5:74323288-74326724 | NT | MOF | OK | 0.687472 | 1.46567 | 2.1319705 | 1.09218 | 1.26891 | 0.0366 | 0.367214 | no |
| XLOC_020337 | XLOC_020337 | ELFN1 | chr7:1748797-1787590 | NT | MOF | OK | 0.791271 | 1.68513 | 2.1296496 | 1.09061 | 1.21921 | 0.03605 | 0.364415 | no |
| XLOC_021631 | XLOC_021631 | RDH10 | chr8:74206836-74237520 | NT | MOF | OK | 55.3271 | 117.548 | 2.1246008 | 1.0872 | 2.17415 | 0.00045 | 0.0205472 | yes |
| XLOC_006252 | XLOC_006252 | PNP | chr14:20937537-20946165 | NT | MOF | OK | 8.38543 | 17.7099 | 2.1119847 | 1.0786 | 2.10748 | 0.0007 | 0.0283278 | yes |
| XLOC_022222 | XLOC_022222 | FAM83H | chr8:144806102-144815914 | NT | MOF | OK | 0.990622 | 2.09174 | 2.111542 | 1.0783 | 1.61589 | 0.00565 | 0.121646 | no |
| XLOC_012490 | XLOC_012490 | B3GALT1 | chr2:168675181-168727366 | NT | MOF | OK | 0.786502 | 1.66071 | 2.111514 | 1.07828 | 1.16297 | 0.05015 | 0.441963 | no |

| | | | | | | | | | | | | | |
|---|---|---|---|---|---|---|---|---|---|---|---|---|---|
| XLOC_009510 | XLOC_009510 | PMP22 | chr17:15133095-15168644 | NT | MOF | OK | 22.053 | 46.5404 | 2.1103886 | 1.07751 | 2.25839 | 5.00E-05 | 0.00368902 yes |
| XLOC_014563 | XLOC_014563 | APOBEC3G | chr22:39473009-39483748 | NT | MOF | OK | 0.874172 | 1.84331 | 2.1086354 | 1.07631 | 1.09178 | 0.06155 | 0.488044 no |
| XLOC_001073 | XLOC_001073 | CNIH3 | chr1:224804178-224928249 | NT | MOF | OK | 2.18718 | 4.60957 | 2.1075403 | 1.07556 | 1.58371 | 0.0077 | 0.148124 no |
| XLOC_006076 | XLOC_006076 | TRPC4 | chr13:38210772-38443939 | NT | MOF | OK | 0.483688 | 1.01836 | 2.1054068 | 1.0741 | 1.01026 | 0.04 | 0.385965 no |
| XLOC_015613 | XLOC_015613 | NEK10 | chr3:27257096-27410912 | NT | MOF | OK | 0.752547 | 1.58347 | 2.1041476 | 1.07323 | 1.24821 | 0.02985 | 0.329849 no |
| XLOC_003949 | XLOC_003949 | PIDD | chr11:799178-805250 | NT | MOF | OK | 5.48514 | 11.5397 | 2.1038114 | 1.07301 | 2.0255 | 0.0005 | 0.0219203 yes |
| XLOC_011323 | XLOC_011323 | STAP2 | chr19:4324039-4338847 | NT | MOF | OK | 0.382022 | 0.803192 | 2.1024758 | 1.07209 | 0.882936 | 0.1235 | 0.665042 no |
| XLOC_009212 | XLOC_009212 | C17orf82 | chr17:59489111-59490641 | NT | MOF | OK | 1.50545 | 3.16402 | 2.1017105 | 1.07156 | 1.28774 | 0.02655 | 0.309779 no |
| XLOC_016482 | XLOC_016482 | USP53 | chr4:120133781-120216673 | NT | MOF | OK | 13.8739 | 29.1468 | 2.1008368 | 1.07096 | 2.39979 | 5.00E-05 | 0.00368902 yes |
| XLOC_005378 | XLOC_005378 | CPNE8 | chr12:39046001-39299420 | NT | MOF | OK | 2.01298 | 4.22773 | 2.1002345 | 1.07055 | 1.69984 | 0.0046 | 0.104624 no |
| XLOC_013827 | XLOC_013827 | SLC4A11 | chr20:3208062-3219887 | NT | MOF | OK | 0.601352 | 1.26285 | 2.100018 | 1.0704 | 1.2645 | 0.0297 | 0.329395 no |
| XLOC_006544 | XLOC_006544 | MEG8 | chr14:101361106-101373305 | NT | MOF | OK | 1.33222 | 2.79705 | 2.0995406 | 1.07007 | 0.81388 | 0.1748 | 0.768703 no |
| XLOC_016489 | XLOC_016489 | FGF2 | chr4:123747862-123844159 | NT | MOF | OK | 75.787 | 159.058 | 2.0987504 | 1.06953 | 1.84661 | 0.00095 | 0.0354784 yes |
| XLOC_013094 | XLOC_013094 | MALL | chr2:110841446-110874143 | NT | MOF | OK | 4.9228 | 10.3284 | 2.0980743 | 1.06906 | 1.91592 | 0.00105 | 0.0382681 yes |
| XLOC_005425 | XLOC_005425 | PRKAG1 | chr12:49396054-49412629 | NT | MOF | OK | 11.8882 | 24.8991 | 2.0944382 | 1.06656 | 2.05264 | 0.0004 | 0.0189063 yes |
| XLOC_010105 | XLOC_010105 | ARHGAP28 | chr18:6834431-6915712 | NT | MOF | OK | 0.610832 | 1.27905 | 2.0939473 | 1.06622 | 1.36939 | 0.02435 | 0.296116 no |
| XLOC_018266 | XLOC_018266 | CDKN1A | chr6:36644236-36655116 | NT | MOF | OK | 346.435 | 724.496 | 2.0912899 | 1.06439 | 2.14189 | 0.0003 | 0.0149383 yes |
| XLOC_018471 | XLOC_018471 | GJA1 | chr6:121756744-121770873 | NT | MOF | OK | 88.0181 | 183.69 | 2.0869571 | 1.0614 | 2.32262 | 5.00E-05 | 0.00368902 yes |
| XLOC_000639 | XLOC_000639 | LIX1L | chr1:145477084-145499091 | NT | MOF | OK | 15.6152 | 32.5759 | 2.086166 | 1.06085 | 2.06181 | 0.0004 | 0.0189063 yes |
| XLOC_016931 | XLOC_016931 | ELF2 | chr4:139978870-140060630 | NT | MOF | OK | 3.67291 | 7.65538 | 2.0842819 | 1.05955 | 1.94309 | 0.00135 | 0.0457563 yes |
| XLOC_004742 | XLOC_004742 | KIAA1467 | chr12:13197314-13236383 | NT | MOF | OK | 1.89901 | 3.9509 | 2.0805051 | 1.05693 | 1.80177 | 0.00265 | 0.0738825 no |
| XLOC_017914 | XLOC_017914 | GEMIN5 | chr5:154266975-154317776 | NT | MOF | OK | 2.68909 | 5.57779 | 2.0742296 | 1.05258 | 2.00127 | 0.00095 | 0.0354784 yes |
| XLOC_024024 | XLOC_024024 | ACSL4 | chrX:108884563-108976621 | NT | MOF | OK | 25.1988 | 52.2489 | 2.0734678 | 1.05205 | 2.37906 | 5.00E-05 | 0.00368902 yes |
| XLOC_020702 | XLOC_020702 | MDFIC | chr7:114562208-114659970 | NT | MOF | OK | 11.9958 | 24.8721 | 2.0734007 | 1.05199 | 2.1167 | 5.00E-05 | 0.00368902 yes |
| XLOC_009956 | XLOC_009956 | FDXR | chr17:72858618-72869156 | NT | MOF | OK | 9.06628 | 18.7786 | 2.0712575 | 1.05051 | 1.99355 | 0.00085 | 0.0324448 yes |
| XLOC_015407 | XLOC_015407 | ARHGEF26 | chr3:153742189-153975616 | NT | MOF | OK | 1.21678 | 2.51608 | 2.0678183 | 1.04811 | 1.64173 | 0.0044 | 0.101992 no |
| XLOC_006157 | XLOC_006157 | KCTD12 | chr13:77454303-77460540 | NT | MOF | OK | 14.4193 | 29.8125 | 2.0675414 | 1.04791 | 2.34487 | 5.00E-05 | 0.00368902 yes |
| XLOC_016068 | XLOC_016068 | ZMAT3 | chr3:178735010-178789656 | NT | MOF | OK | 17.8428 | 36.8743 | 2.0666207 | 1.04728 | 1.97843 | 0.0012 | 0.0422093 yes |
| XLOC_016491 | XLOC_016491 | SPRY1 | chr4:124317949-124324915 | NT | MOF | OK | 3.97329 | 8.21006 | 2.0663128 | 1.04706 | 1.69304 | 0.0024 | 0.0694737 no |
| XLOC_007290 | XLOC_007290 | CD276 | chr15:73976621-74006859 | NT | MOF | OK | 26.3866 | 54.5194 | 2.0661775 | 1.04696 | 2.3829 | 5.00E-05 | 0.00368902 yes |
| XLOC_017097 | XLOC_017097 | RAI14 | chr5:34656432-34832717 | NT | MOF | OK | 59.417 | 122.39 | 2.0598482 | 1.04254 | 2.24898 | 5.00E-05 | 0.00368902 yes |
| XLOC_018644 | XLOC_018644 | ADTRP | chr6:11713887-11779280 | NT | MOF | OK | 0.581555 | 1.19532 | 2.055386 | 1.03941 | 0.901371 | 0.10175 | 0.61344 no |
| XLOC_000344 | XLOC_000344 | CCDC24 | chr1:44457279-44497164 | NT | MOF | OK | 0.513497 | 1.05259 | 2.0498464 | 1.03551 | 0.352114 | 0.6796 | 0.999021 no |
| XLOC_009654 | XLOC_009654 | SNORA21 | chr17:37006320-37010053 | NT | MOF | OK | 14.5036 | 29.7232 | 2.0493671 | 1.03518 | 1.04901 | 0.59425 | 0.999021 no |
| XLOC_007738 | XLOC_007738 | RPS17L | chr15:83205503-83209208 | NT | MOF | OK | 0.352959 | 0.720314 | 2.0407866 | 1.02913 | 0.59415 | 0.3439 | 0.92512 no |
| XLOC_000812 | XLOC_000812 | PEA15 | chr1:160175124-160185162 | NT | MOF | OK | 107.074 | 218.387 | 2.0395894 | 1.02828 | 2.29088 | 5.00E-05 | 0.00368902 yes |
| XLOC_005580 | XLOC_005580 | PTPRB | chr12:70910631-71031220 | NT | MOF | OK | 0.712889 | 1.45189 | 2.0366284 | 1.02619 | 1.45802 | 0.01065 | 0.18227 no |
| XLOC_012567 | XLOC_012567 | SLC39A10 | chr2:196521531-196602426 | NT | MOF | OK | 9.27047 | 18.8751 | 2.0360456 | 1.02577 | 2.21738 | 5.00E-05 | 0.00368902 yes |
| XLOC_014937 | XLOC_014937 | PRRT3-AS1 | chr3:9987225-9996471 | NT | MOF | OK | 0.672162 | 1.36706 | 2.0338252 | 1.0242 | 0.323068 | 0.6278 | 0.999021 no |
| XLOC_004743 | XLOC_004743 | EMP1 | chr12:13349601-13369708 | NT | MOF | OK | 61.6468 | 125.156 | 2.0302108 | 1.02163 | 2.22315 | 0.0001 | 0.00661202 yes |
| XLOC_007018 | XLOC_007018 | PAR5 | chr15:25230006-25233379 | NT | MOF | OK | 1.92496 | 3.9062 | 2.029237 | 1.02094 | 1.59056 | 0.0052 | 0.115662 no |
| XLOC_017458 | XLOC_017458 | CCNG1 | chr5:162864576-162872022 | NT | MOF | OK | 55.3048 | 112.027 | 2.0256289 | 1.01837 | 2.19864 | 5.00E-05 | 0.00368902 yes |

| | | | | | | | | | | | | | | |
|---|---|---|---|---|---|---|---|---|---|---|---|---|---|---|
| XLOC_006991 | XLOC_006991 | AHNAK2 | chr14:105403590-105444694 | NT | MOF | OK | 2.92267 | 5.89604 | 2.0173472 | 1.01246 | 2.20435 | 0.00015 | 0.00902985 | yes |
| XLOC_021508 | XLOC_021508 | TNFRSF10C | chr8:22941867-22974950 | NT | MOF | OK | 2.08891 | 4.20376 | 2.012418 | 1.00893 | 1.27655 | 0.03125 | 0.336111 | no |
| XLOC_009690 | XLOC_009690 | KRTAP1-5 | chr17:39182278-39183454 | NT | MOF | OK | 8.88775 | 17.8683 | 2.0104413 | 1.00751 | 1.67164 | 0.00445 | 0.102367 | no |
| XLOC_003187 | XLOC_003187 | FAM196A | chr10:128594022-129250780 | NT | MOF | OK | 0.538652 | 1.08284 | 2.0102775 | 1.0074 | 0.307523 | 0.6438 | 0.999021 | no |
| XLOC_005366 | XLOC_005366 | TMTC1 | chr12:29653745-29937692 | NT | MOF | OK | 17.722 | 35.5304 | 2.0048753 | 1.00351 | 2.22322 | 0.0001 | 0.00661202 | yes |
| XLOC_000491 | XLOC_000491 | SH3GLB1 | chr1:87170252-87213867 | NT | MOF | OK | 15.7738 | 31.5871 | 2.0025042 | 1.0018 | 2.25022 | 0.00015 | 0.00902985 | yes |
| XLOC_016295 | XLOC_016295 | SHISA3 | chr4:42399855-42404504 | NT | MOF | OK | 2.61997 | 1.30909 | -2.0013674 | -1.001 | -1.1563 | 0.04405 | 0.407496 | no |
| XLOC_021701 | XLOC_021701 | ATP6V1C1 | chr8:104033247-104085285 | NT | MOF | OK | 15.2819 | 7.62936 | -2.0030383 | -1.0022 | -2.19204 | 0.0003 | 0.0149383 | yes |
| XLOC_017341 | XLOC_017341 | REEP2 | chr5:137774689-137782658 | NT | MOF | OK | 7.95881 | 3.96749 | -2.0060063 | -1.0043 | -1.63708 | 0.00545 | 0.11925 | no |
| XLOC_023568 | XLOC_023568 | TMEM164 | chrX:109245862-109421016 | NT | MOF | OK | 3.40664 | 1.69756 | -2.0067862 | -1.0049 | -1.74742 | 0.00325 | 0.0834926 | no |
| XLOC_000492 | XLOC_000492 | HS2ST1 | chr1:87380334-87575681 | NT | MOF | OK | 15.5704 | 7.75277 | -2.008366 | -1.006 | -1.50777 | 0.00755 | 0.146168 | no |
| XLOC_022132 | XLOC_022132 | LRP12 | chr8:105501458-105601252 | NT | MOF | OK | 18.4257 | 9.15216 | -2.0132624 | -1.0095 | -2.13 | 0.0002 | 0.0112558 | yes |
| XLOC_016088 | XLOC_016088 | MAGEF1 | chr3:184428154-184429836 | NT | MOF | OK | 17.1257 | 8.50207 | -2.0142977 | -1.0103 | -1.88685 | 0.0013 | 0.0445609 | yes |
| XLOC_007877 | XLOC_007877 | CCNF | chr16:2479394-2508859 | NT | MOF | OK | 4.28593 | 2.1275 | -2.0145382 | -1.0105 | -1.73161 | 0.0032 | 0.0829122 | no |
| XLOC_017295 | XLOC_017295 | SLC12A2 | chr5:127419482-127525380 | NT | MOF | OK | 7.66869 | 3.80439 | -2.0157476 | -1.0113 | -2.07253 | 0.00025 | 0.0132096 | yes |
| XLOC_018082 | XLOC_018082 | GMNN | chr6:24775158-24786325 | NT | MOF | OK | 6.83618 | 3.38611 | -2.0188889 | -1.0136 | -1.4099 | 0.01645 | 0.233074 | no |
| XLOC_009547 | XLOC_009547 | MFAP4 | chr17:19240866-19290532 | NT | MOF | OK | 76.4994 | 37.8677 | -2.0201755 | -1.0145 | -1.60471 | 0.01065 | 0.18227 | no |
| XLOC_014574 | XLOC_014574 | TNRC6B | chr22:40440820-40731812 | NT | MOF | OK | 5.84188 | 2.88858 | -2.0224055 | -1.0161 | -2.19831 | 0.0003 | 0.0149383 | yes |
| XLOC_015254 | XLOC_015254 | CD200 | chr3:112051915-112081658 | NT | MOF | OK | 3.71582 | 1.83712 | -2.0226333 | -1.0162 | -1.33152 | 0.01915 | 0.257946 | no |
| XLOC_021046 | XLOC_021046 | NACAD | chr7:45120035-45128493 | NT | MOF | OK | 1.86227 | 0.920468 | -2.0231773 | -1.0166 | -1.42408 | 0.01275 | 0.200618 | no |
| XLOC_014528 | XLOC_014528 | MCM5 | chr22:35796115-35820495 | NT | MOF | OK | 8.05353 | 3.97986 | -2.0235712 | -1.0169 | -1.73547 | 0.0026 | 0.072993 | no |
| XLOC_008034 | XLOC_008034 | PRRT2 | chr16:29823408-29827202 | NT | MOF | OK | 1.17489 | 0.580324 | -2.0245415 | -1.0176 | -1.09418 | 0.0511 | 0.443985 | no |
| XLOC_002139 | XLOC_002139 | NUAK2 | chr1:205271190-205290883 | NT | MOF | OK | 6.85397 | 3.38145 | -2.0269322 | -1.0193 | -1.80942 | 0.00295 | 0.0793222 | no |
| XLOC_000470 | XLOC_000470 | FAM73A | chr1:78245308-78345225 | NT | MOF | OK | 4.03502 | 1.99038 | -2.0272611 | -1.0195 | -1.96356 | 0.0011 | 0.03997 | yes |
| XLOC_004954 | XLOC_004954 | SLC16A7 | chr12:59989820-60183635 | NT | MOF | OK | 7.17757 | 3.53721 | -2.0291614 | -1.0209 | -2.17988 | 0.0002 | 0.0112558 | yes |
| XLOC_001062 | XLOC_001062 | MARC1 | chr1:220960038-220987741 | NT | MOF | OK | 0.822372 | 0.405078 | -2.0301572 | -1.0216 | -0.976376 | 0.08565 | 0.569119 | no |
| XLOC_001975 | XLOC_001975 | NR1I3 | chr1:161195832-161208000 | NT | MOF | OK | 0.851913 | 0.419316 | -2.031673 | -1.0227 | -0.343299 | 0.1996 | 0.796294 | no |
| XLOC_022729 | XLOC_022729 | TTC39B | chr9:15170841-15307358 | NT | MOF | OK | 3.99958 | 1.96725 | -2.0330817 | -1.0237 | -1.61646 | 0.0031 | 0.0815435 | no |
| XLOC_016034 | XLOC_016034 | TRIM59 | chr3:160153290-160167626 | NT | MOF | OK | 4.40908 | 2.16732 | -2.0343466 | -1.0246 | -1.70932 | 0.0047 | 0.106299 | no |
| XLOC_019001 | XLOC_019001 | MMS22L | chr6:97372495-97862283 | NT | MOF | OK | 1.04778 | 0.512837 | -2.0431053 | -1.0308 | -1.46703 | 0.01245 | 0.197957 | no |
| XLOC_000207 | XLOC_000207 | HMGN2 | chr1:26798901-26803133 | NT | MOF | OK | 43.8444 | 21.4501 | -2.0440184 | -1.0314 | -2.22514 | 0.00015 | 0.00902985 | yes |
| XLOC_012821 | XLOC_012821 | DNAJC27 | chr2:25166504-25194963 | NT | MOF | OK | 1.01865 | 0.497959 | -2.0456503 | -1.0326 | -1.28198 | 0.02505 | 0.298626 | no |
| XLOC_005050 | XLOC_005050 | GAS2L3 | chr12:100967488-101018685 | NT | MOF | OK | 2.26922 | 1.10897 | -2.0462411 | -1.033 | -1.2701 | 0.0312 | 0.336111 | no |
| XLOC_020756 | XLOC_020756 | MEST | chr7:130126015-130371406 | NT | MOF | OK | 37.621 | 18.3761 | -2.0472788 | -1.0337 | -1.7853 | 0.0042 | 0.0984884 | no |
| XLOC_023286 | XLOC_023286 | GRPR | chrX:16141423-16171641 | NT | MOF | OK | 1.47057 | 0.718004 | -2.0481362 | -1.0343 | -1.14231 | 0.04345 | 0.403488 | no |
| XLOC_003168 | XLOC_003168 | CUZD1,FAM24B | chr10:124591670-124658230 | NT | MOF | OK | 1.22064 | 0.595869 | -2.0485039 | -1.0346 | -0.688899 | 0.18715 | 0.780867 | no |
| XLOC_002102 | XLOC_002102 | TMEM9 | chr1:201103899-201123632 | NT | MOF | OK | 18.1046 | 8.82821 | -2.0507668 | -1.0362 | -1.91083 | 0.00115 | 0.0411686 | yes |
| XLOC_005224 | XLOC_005224 | FOXM1 | chr12:2945981-2998691 | NT | MOF | OK | 8.29392 | 4.04385 | -2.050996 | -1.0363 | -1.44368 | 0.01765 | 0.244633 | no |
| XLOC_016975 | XLOC_016975 | C4orf46 | chr4:159587830-159593202 | NT | MOF | OK | 3.43251 | 1.67332 | -2.0513171 | -1.0366 | -1.56268 | 0.00775 | 0.148144 | no |
| XLOC_019047 | XLOC_019047 | TSPYL4 | chr6:116571130-116575261 | NT | MOF | OK | 7.36372 | 3.58958 | -2.0514155 | -1.0366 | -1.98679 | 0.0013 | 0.0445609 | yes |
| XLOC_009967 | XLOC_009967 | GRB2 | chr17:73314156-73401790 | NT | MOF | OK | 36.6602 | 17.8514 | -2.0536316 | -1.0382 | -2.33251 | 5.00E-05 | 0.00368902 | yes |
| XLOC_013238 | XLOC_013238 | CHN1 | chr2:175664041-175870107 | NT | MOF | OK | 53.9547 | 26.2093 | -2.058609 | -1.0417 | -2.13492 | 0.00025 | 0.0132096 | yes |

| | | | | | | | | | | | | | | |
|---|---|---|---|---|---|---|---|---|---|---|---|---|---|---|
| XLOC_005040 | XLOC_005040 | TMPO | chr12:98906750-98944157 | NT | MOF | OK | 9.32925 | 4.52974 | -2.0595553 | -1.0423 | -1.96086 | 0.00075 | 0.0295603 | yes |
| XLOC_009834 | XLOC_009834 | FAM117A | chr17:47787686-47841518 | NT | MOF | OK | 1.2411 | 0.602312 | -2.06056 | -1.043 | -1.10397 | 0.06195 | 0.488654 | no |
| XLOC_001671 | XLOC_001671 | SASS6 | chr1:100549101-100598511 | NT | MOF | OK | 1.17891 | 0.572118 | -2.0606064 | -1.0431 | -1.24731 | 0.0273 | 0.3149 | no |
| XLOC_010799 | XLOC_010799 | ARHGAP33 | chr19:36266416-36279724 | NT | MOF | OK | 0.943969 | 0.456428 | -2.0681663 | -1.0484 | -1.13658 | 0.0472 | 0.424309 | no |
| XLOC_004118 | XLOC_004118 | CCDC34 | chr11:27360060-27384795 | NT | MOF | OK | 3.35565 | 1.62181 | -2.0690771 | -1.049 | -1.26374 | 0.0369 | 0.369 | no |
| XLOC_022809 | XLOC_022809 | FANCG | chr9:35073834-35080013 | NT | MOF | OK | 2.40736 | 1.16185 | -2.0720059 | -1.051 | -1.36836 | 0.01725 | 0.241022 | no |
| XLOC_010266 | XLOC_010266 | ENOSF1 | chr18:596997-712662 | NT | MOF | OK | 2.89123 | 1.39334 | -2.0750355 | -1.0531 | -1.25556 | 0.02855 | 0.323763 | no |
| XLOC_007196 | XLOC_007196 | SEMA6D | chr15:47476402-48066420 | NT | MOF | OK | 4.89096 | 2.35 | -2.0812596 | -1.0575 | -1.7891 | 0.00165 | 0.0535255 | no |
| XLOC_012663 | XLOC_012663 | SPEG | chr2:220299699-220358354 | NT | MOF | OK | 7.22019 | 3.4664 | -2.0829073 | -1.0586 | -1.41256 | 0.01825 | 0.24952 | no |
| XLOC_004811 | XLOC_004811 | TMEM106C | chr12:48357329-48362661 | NT | MOF | OK | 11.8763 | 5.69544 | -2.0852296 | -1.0602 | -1.75378 | 0.00405 | 0.0957129 | no |
| XLOC_019089 | XLOC_019089 | SLC2A12 | chr6:134308718-134373789 | NT | MOF | OK | 1.58725 | 0.760516 | -2.0870698 | -1.0615 | -1.49945 | 0.00985 | 0.172983 | no |
| XLOC_016502 | XLOC_016502 | C4orf33 | chr4:130014828-130033843 | NT | MOF | OK | 2.1017 | 1.00507 | -2.0910981 | -1.0643 | -1.16341 | 0.0465 | 0.420202 | no |
| XLOC_004405 | XLOC_004405 | CHRDL2 | chr11:74407473-74442186 | NT | MOF | OK | 1.34237 | 0.641208 | -2.0935016 | -1.0659 | -0.879525 | 0.3382 | 0.92125 | no |
| XLOC_015748 | XLOC_015748 | POC1A | chr3:52109248-52188706 | NT | MOF | OK | 2.58669 | 1.23498 | -2.0945197 | -1.0666 | -1.28839 | 0.02635 | 0.30865 | no |
| XLOC_000519 | XLOC_000519 | SLC44A3 | chr1:95285897-95360803 | NT | MOF | OK | 0.751667 | 0.358853 | -2.0946376 | -1.0667 | -0.83452 | 0.0777 | 0.543451 | no |
| XLOC_017872 | XLOC_017872 | PRELID2 | chr5:145138581-145214899 | NT | MOF | OK | 1.26644 | 0.60378 | -2.097519 | -1.0687 | -1.11983 | 0.04575 | 0.417163 | no |
| XLOC_000573 | XLOC_000573 | GSTM5 | chr1:110254863-110260890 | NT | MOF | OK | 0.917562 | 0.437387 | -2.0978264 | -1.0689 | -0.867399 | 0.14165 | 0.701582 | no |
| XLOC_023908 | XLOC_023908 | SPIN4 | chrX:62567106-62571218 | NT | MOF | OK | 1.54561 | 0.735353 | -2.1018613 | -1.0717 | -1.40378 | 0.01515 | 0.223011 | no |
| XLOC_023549 | XLOC_023549 | TMSB15B | chrX:103139054-103401708 | NT | MOF | OK | 1.88914 | 0.897704 | -2.104413 | -1.0734 | -0.494109 | 0.435 | 0.969292 | no |
| XLOC_021481 | XLOC_021481 | PDGFRL | chr8:17433941-17500642 | NT | MOF | OK | 8.68779 | 4.12809 | -2.1045544 | -1.0735 | -1.70901 | 0.00475 | 0.10703 | no |
| XLOC_008489 | XLOC_008489 | SULT1A1 | chr16:28616907-28634907 | NT | MOF | OK | 2.42305 | 1.15011 | -2.1067985 | -1.0751 | -1.00177 | 0.0607 | 0.485119 | no |
| XLOC_023252 | XLOC_023252 | GYG2 | chrX:2746862-2800861 | NT | MOF | OK | 2.99052 | 1.41532 | -2.1129639 | -1.0793 | -1.43521 | 0.0137 | 0.20957 | no |
| XLOC_015855 | XLOC_015855 | KIAA1524 | chr3:108268717-108413693 | NT | MOF | OK | 2.4978 | 1.18005 | -2.11669 | -1.0818 | -1.11368 | 0.06715 | 0.504666 | no |
| XLOC_006759 | XLOC_006759 | SPTSSA | chr14:34902143-34931468 | NT | MOF | OK | 11.6346 | 5.48702 | -2.1203859 | -1.0843 | -2.04596 | 0.00065 | 0.0267517 | yes |
| XLOC_017607 | XLOC_017607 | LMBRD2 | chr5:36103413-36152015 | NT | MOF | OK | 4.98675 | 2.34987 | -2.1221387 | -1.0855 | -1.8115 | 0.00205 | 0.0626389 | no |
| XLOC_012699 | XLOC_012699 | PTMA | chr2:232573234-232578250 | NT | MOF | OK | 253.009 | 119.203 | -2.1225053 | -1.0858 | -2.31049 | 5.00E-05 | 0.00368902 | yes |
| XLOC_008766 | XLOC_008766 | GSG2 | chr17:3617918-3704537 | NT | MOF | OK | 1.81633 | 0.85547 | -2.1231954 | -1.0862 | -0.844362 | 0.17385 | 0.768275 | no |
| XLOC_006777 | XLOC_006777 | SEC23A | chr14:39501122-39572437 | NT | MOF | OK | 116.654 | 54.8108 | -2.1283032 | -1.0897 | -2.43262 | 5.00E-05 | 0.00368902 | yes |
| XLOC_022998 | XLOC_022998 | EPB41L4B | chr9:111934253-112083021 | NT | MOF | OK | 0.787576 | 0.370007 | -2.1285435 | -1.0899 | -1.14486 | 0.04825 | 0.428968 | no |
| XLOC_013167 | XLOC_013167 | MCM6 | chr2:136597195-136634047 | NT | MOF | OK | 5.64486 | 2.64811 | -2.1316562 | -1.092 | -1.87857 | 0.00225 | 0.0668919 | no |
| XLOC_018461 | XLOC_018461 | KPNA5 | chr6:117002366-117063030 | NT | MOF | OK | 3.82932 | 1.79299 | -2.1357174 | -1.0947 | -1.48913 | 0.013 | 0.202968 | no |
| XLOC_004402 | XLOC_004402 | PGM2L1 | chr11:74041360-74109502 | NT | MOF | OK | 6.10849 | 2.85851 | -2.136949 | -1.0956 | -2.24091 | 0.00015 | 0.00902985 | yes |
| XLOC_003545 | XLOC_003545 | DAGLA | chr11:61447904-61514474 | NT | MOF | OK | 2.56037 | 1.19701 | -2.1389713 | -1.0969 | -1.73254 | 0.00235 | 0.0685181 | no |
| XLOC_018543 | XLOC_018543 | UST | chr6:149068270-149398126 | NT | MOF | OK | 9.96506 | 4.65767 | -2.1394946 | -1.0973 | -2.21391 | 0.0002 | 0.0112558 | yes |
| XLOC_003899 | XLOC_003899 | CHEK1 | chr11:125495030-125550793 | NT | MOF | OK | 5.90273 | 2.75555 | -2.1421241 | -1.099 | -1.7472 | 0.0036 | 0.088357 | no |
| XLOC_004119 | XLOC_004119 | LGR4 | chr11:27387507-27494334 | NT | MOF | OK | 4.21483 | 1.96742 | -2.1423133 | -1.0992 | -1.84451 | 0.00205 | 0.0626389 | no |
| XLOC_008899 | XLOC_008899 | TRIM16L | chr17:18625401-18639431 | NT | MOF | OK | 2.49223 | 1.16324 | -2.1424899 | -1.0993 | -1.32038 | 0.02075 | 0.268817 | no |
| XLOC_007509 | XLOC_007509 | MRPL42P5 | chr15:40824082-40824749 | NT | MOF | OK | 0.749728 | 0.349403 | -2.14574 | -1.1015 | -0.73738 | 0.24125 | 0.844166 | no |
| XLOC_007362 | XLOC_007362 | TM6SF1 | chr15:83776323-83806111 | NT | MOF | OK | 2.56863 | 1.19536 | -2.1488338 | -1.1036 | -1.28029 | 0.03125 | 0.336111 | no |
| XLOC_005553 | XLOC_005553 | LOC100506844 | chr12:58325231-58329947 | NT | MOF | OK | 4.16536 | 1.93358 | -2.1542217 | -1.1072 | -1.13086 | 0.04985 | 0.440281 | no |
| XLOC_006896 | XLOC_006896 | ACYP1 | chr14:75519927-75530736 | NT | MOF | OK | 2.95896 | 1.37314 | -2.1548859 | -1.1076 | -0.971699 | 0.09095 | 0.581965 | no |
| XLOC_023772 | XLOC_023772 | FANCB | chrX:14861528-14891184 | NT | MOF | OK | 0.88491 | 0.409407 | -2.1614433 | -1.112 | -1.13825 | 0.0394 | 0.381392 | no |

| | | | | | | | | | | | | | | | |
|---|---|---|---|---|---|---|---|---|---|---|---|---|---|---|---|
| XLOC_012222 | XLOC_012222 | GMCL1 | chr2:70056817-70106727 | NT | MOF | OK | 6.08234 | 2.80889 | -2.1653892 | -1.1146 | -1.76573 | 0.0017 | 0.054418 | no |
| XLOC_018680 | XLOC_018680 | HIST1H2AD,HIST | chr6:26197011-26199521 | NT | MOF | OK | 51.5936 | 23.8084 | -2.1670335 | -1.1157 | -1.94146 | 0.0013 | 0.0445609 | yes |
| XLOC_006915 | XLOC_006915 | CEP128 | chr14:80962820-81405884 | NT | MOF | OK | 0.855629 | 0.394804 | -2.1672247 | -1.1159 | -1.259 | 0.03495 | 0.357686 | no |
| XLOC_010613 | XLOC_010613 | RNASEH2A | chr19:12917427-12924462 | NT | MOF | OK | 8.53267 | 3.9339 | -2.1690104 | -1.117 | -1.55608 | 0.0065 | 0.132185 | no |
| XLOC_011368 | XLOC_011368 | PEX11G | chr19:7541755-7553924 | NT | MOF | OK | 1.39374 | 0.642514 | -2.1691979 | -1.1172 | -0.926204 | 0.09655 | 0.597879 | no |
| XLOC_018054 | XLOC_018054 | EDN1 | chr6:12290528-12297427 | NT | MOF | OK | 4.4973 | 2.07126 | -2.171287 | -1.1186 | -1.60391 | 0.0066 | 0.133769 | no |
| XLOC_023427 | XLOC_023427 | GPR173 | chrX:53078505-53109796 | NT | MOF | OK | 1.55107 | 0.712587 | -2.1766746 | -1.1221 | -1.43055 | 0.01785 | 0.245846 | no |
| XLOC_022083 | XLOC_022083 | LOC100127983 | chr8:91803920-91997485 | NT | MOF | OK | 8.71858 | 4.00406 | -2.1774349 | -1.1226 | -1.19191 | 0.0473 | 0.424577 | no |
| XLOC_023069 | XLOC_023069 | GOLGA1 | chr9:127640572-127703386 | NT | MOF | OK | 5.48413 | 2.51332 | -2.1820262 | -1.1257 | -2.09321 | 0.00055 | 0.0234331 | yes |
| XLOC_000111 | XLOC_000111 | PDPN | chr1:13910251-13944452 | NT | MOF | OK | 0.721276 | 0.33035 | -2.1833692 | -1.1266 | -0.885787 | 0.1288 | 0.675221 | no |
| XLOC_016260 | XLOC_016260 | PI4K2B | chr4:25235652-25280831 | NT | MOF | OK | 11.1199 | 5.06314 | -2.1962458 | -1.135 | -2.26025 | 0.00015 | 0.00902985 | yes |
| XLOC_011966 | XLOC_011966 | IL11 | chr19:55875749-55881831 | NT | MOF | OK | 11.0847 | 5.04106 | -2.1988828 | -1.1368 | -1.70813 | 0.00275 | 0.0759703 | no |
| XLOC_017257 | XLOC_017257 | CAMK4 | chr5:110559946-110820748 | NT | MOF | OK | 2.04694 | 0.930228 | -2.2004713 | -1.1378 | -1.29091 | 0.03235 | 0.343063 | no |
| XLOC_007388 | XLOC_007388 | FANCI | chr15:89787193-89878026 | NT | MOF | OK | 4.42274 | 2.00919 | -2.2012552 | -1.1383 | -0.862073 | 0.18275 | 0.77725 | no |
| XLOC_012948 | XLOC_012948 | C1D | chr2:68269331-68290159 | NT | MOF | OK | 9.89837 | 4.49329 | -2.2029226 | -1.1394 | -1.65352 | 0.0065 | 0.132185 | no |
| XLOC_007111 | XLOC_007111 | ARHGAP11B | chr15:30918878-30931013 | NT | MOF | OK | 0.96541 | 0.437538 | -2.2064598 | -1.1417 | -0.851528 | 0.16015 | 0.741322 | no |
| XLOC_011308 | XLOC_011308 | TBXA2R | chr19:3594503-3606831 | NT | MOF | OK | 2.04705 | 0.925376 | -2.2121278 | -1.1454 | -1.39449 | 0.0179 | 0.245846 | no |
| XLOC_016781 | XLOC_016781 | ADAMTS3 | chr4:73146685-73434516 | NT | MOF | OK | 0.805335 | 0.363946 | -2.2127871 | -1.1459 | -1.38416 | 0.01745 | 0.242975 | no |
| XLOC_010258 | XLOC_010258 | KCNG2 | chr18:77623667-77659816 | NT | MOF | OK | 1.97182 | 0.89101 | -2.2130167 | -1.146 | -1.09809 | 0.0599 | 0.482871 | no |
| XLOC_008032 | XLOC_008032 | KIF22 | chr16:29802033-29816706 | NT | MOF | OK | 6.28237 | 2.83643 | -2.2148863 | -1.1472 | -1.75611 | 0.0028 | 0.077 | no |
| XLOC_005847 | XLOC_005847 | RFC3 | chr13:34392205-34540695 | NT | MOF | OK | 2.81044 | 1.26839 | -2.2157538 | -1.1478 | -1.48525 | 0.0106 | 0.181929 | no |
| XLOC_023528 | XLOC_023528 | ARMCX4 | chrX:100673250-100790975 | NT | MOF | OK | 2.27751 | 1.02603 | -2.2197304 | -1.1504 | -1.81653 | 0.0021 | 0.063525 | no |
| XLOC_017716 | XLOC_017716 | DHFR | chr5:79922044-80172634 | NT | MOF | OK | 3.62888 | 1.63442 | -2.2202861 | -1.1507 | -1.07605 | 0.0674 | 0.504666 | no |
| XLOC_010501 | XLOC_010501 | TNFAIP8L1 | chr19:4639526-4655580 | NT | MOF | OK | 0.85873 | 0.386355 | -2.222645 | -1.1523 | -1.24785 | 0.0272 | 0.314646 | no |
| XLOC_021170 | XLOC_021170 | PON2 | chr7:95034173-95064384 | NT | MOF | OK | 33.8603 | 15.1552 | -2.2342364 | -1.1598 | -2.39562 | 5.00E-05 | 0.00368902 | yes |
| XLOC_012803 | XLOC_012803 | TTC32 | chr2:20096513-20101744 | NT | MOF | OK | 1.4614 | 0.654034 | -2.2344404 | -1.1599 | -0.950614 | 0.1235 | 0.665042 | no |
| XLOC_007146 | XLOC_007146 | RAD51 | chr15:40987326-41024356 | NT | MOF | OK | 1.57214 | 0.702983 | -2.2363841 | -1.1612 | -1.22248 | 0.0247 | 0.297087 | no |
| XLOC_015762 | XLOC_015762 | MUSTN1,TMEM1 | chr3:52867130-52931597 | NT | MOF | OK | 7.1856 | 3.20758 | -2.2401935 | -1.1636 | -1.72967 | 0.00405 | 0.0957129 | no |
| XLOC_013518 | XLOC_013518 | MCM8 | chr20:5931297-5975831 | NT | MOF | OK | 2.6749 | 1.19203 | -2.2439871 | -1.1661 | -1.64187 | 0.0062 | 0.128239 | no |
| XLOC_022935 | XLOC_022935 | ECM2 | chr9:95059639-95432547 | NT | MOF | OK | 0.993407 | 0.442091 | -2.2470645 | -1.168 | -0.448652 | 0.5409 | 0.999021 | no |
| XLOC_015113 | XLOC_015113 | SEMA3B | chr3:50305039-50314572 | NT | MOF | OK | 8.93766 | 3.97549 | -2.2481908 | -1.1688 | -2.19611 | 0.0002 | 0.0112558 | yes |
| XLOC_021199 | XLOC_021199 | MCM7 | chr7:99690403-99723128 | NT | MOF | OK | 13.0166 | 5.7892 | -2.2484281 | -1.1689 | -1.09716 | 0.08045 | 0.553123 | no |
| XLOC_019177 | XLOC_019177 | LINC00473 | chr6:166337535-166403103 | NT | MOF | OK | 1.32894 | 0.589754 | -2.2533802 | -1.1721 | -0.784967 | 0.22285 | 0.827875 | no |
| XLOC_000766 | XLOC_000766 | SYT11 | chr1:155829259-155854990 | NT | MOF | OK | 23.9168 | 10.6058 | -2.255068 | -1.1732 | -2.35017 | 0.0002 | 0.0112558 | yes |
| XLOC_004865 | XLOC_004865 | ESPL1 | chr12:53662082-53687427 | NT | MOF | OK | 1.30983 | 0.579731 | -2.2593755 | -1.1759 | -1.58062 | 0.0088 | 0.16207 | no |
| XLOC_015310 | XLOC_015310 | MCM2 | chr3:127317199-127341278 | NT | MOF | OK | 4.07427 | 1.80164 | -2.2614229 | -1.1772 | -1.84695 | 0.00115 | 0.0411686 | yes |
| XLOC_010194 | XLOC_010194 | LIPG | chr18:47088426-47119278 | NT | MOF | OK | 1.43484 | 0.633968 | -2.2632688 | -1.1784 | -1.18383 | 0.04655 | 0.42034 | no |
| XLOC_022730 | XLOC_022730 | PSIP1 | chr9:15464064-15511003 | NT | MOF | OK | 18.5952 | 8.21079 | -2.2647273 | -1.1793 | -2.31756 | 5.00E-05 | 0.00368902 | yes |
| XLOC_000502 | XLOC_000502 | CDC7 | chr1:91966403-91991321 | NT | MOF | OK | 1.07721 | 0.474987 | -2.2678726 | -1.1813 | -1.3059 | 0.02445 | 0.296141 | no |
| XLOC_009756 | XLOC_009756 | BRCA1 | chr17:41196311-41277500 | NT | MOF | OK | 1.34126 | 0.590458 | -2.2715587 | -1.1837 | -1.41906 | 0.0147 | 0.218782 | no |
| XLOC_004537 | XLOC_004537 | CADM1 | chr11:115044344-115375241 | NT | MOF | OK | 8.44015 | 3.71496 | -2.2719356 | -1.1839 | -2.03365 | 0.00045 | 0.0205472 | yes |
| XLOC_019088 | XLOC_019088 | SLC18B1 | chr6:133090506-133119747 | NT | MOF | OK | 3.43026 | 1.50945 | -2.2725231 | -1.1843 | -1.6304 | 0.00755 | 0.146168 | no |

| | | | | | | | | | | | | | | |
|---|---|---|---|---|---|---|---|---|---|---|---|---|---|---|
| XLOC_020382 | XLOC_020382 | ITGB8 | chr7:20370724-20455382 | NT | MOF | OK | 11.4346 | 5.02977 | -2.2733843 | -1.1849 | -2.34391 | 0.00015 | 0.00902985 | yes |
| XLOC_021519 | XLOC_021519 | CDCA2 | chr8:25316512-25365425 | NT | MOF | OK | 3.42688 | 1.50624 | -2.2751222 | -1.1859 | -1.81937 | 0.00195 | 0.0601913 | no |
| XLOC_009933 | XLOC_009933 | ABCA8 | chr17:66863430-66951533 | NT | MOF | OK | 3.93829 | 1.72967 | -2.2769025 | -1.1871 | -2.12263 | 0.00025 | 0.0132096 | yes |
| XLOC_016598 | XLOC_016598 | TLR3 | chr4:186990308-187006252 | NT | MOF | OK | 1.72503 | 0.756698 | -2.2796809 | -1.1888 | -1.43083 | 0.01335 | 0.206303 | no |
| XLOC_023628 | XLOC_023628 | PHF6 | chrX:133507341-133562822 | NT | MOF | OK | 10.0194 | 4.39259 | -2.2809777 | -1.1897 | -2.26319 | 0.00015 | 0.00902985 | yes |
| XLOC_002473 | XLOC_002473 | ZNF365 | chr10:64133915-64431771 | NT | MOF | OK | 3.20233 | 1.4034 | -2.281837 | -1.1902 | -1.67286 | 0.00265 | 0.0738825 | no |
| XLOC_003622 | XLOC_003622 | KAT5 | chr11:65479472-65488409 | NT | MOF | OK | 10.6818 | 4.66844 | -2.2880877 | -1.1942 | -1.61408 | 0.01175 | 0.191611 | no |
| XLOC_005371 | XLOC_005371 | DENND5B | chr12:31535156-31768285 | NT | MOF | OK | 3.18494 | 1.39177 | -2.2884097 | -1.1944 | -2.20218 | 0.00025 | 0.0132096 | yes |
| XLOC_021380 | XLOC_021380 | EZH2 | chr7:148504463-148581441 | NT | MOF | OK | 3.26528 | 1.42684 | -2.2884696 | -1.1944 | -1.63862 | 0.0047 | 0.106299 | no |
| XLOC_017734 | XLOC_017734 | LYSMD3 | chr5:89811444-89825401 | NT | MOF | OK | 11.2279 | 4.89041 | -2.2959016 | -1.1991 | -2.45921 | 5.00E-05 | 0.00368902 | yes |
| XLOC_010320 | XLOC_010320 | B4GALT6 | chr18:29202208-29264686 | NT | MOF | OK | 0.739517 | 0.322066 | -2.296166 | -1.1992 | -1.33139 | 0.0266 | 0.309779 | no |
| XLOC_004566 | XLOC_004566 | HYOU1 | chr11:118914895-118927925 | NT | MOF | OK | 55.2924 | 24.0486 | -2.2991941 | -1.2011 | -2.68147 | 5.00E-05 | 0.00368902 | yes |
| XLOC_012038 | XLOC_012038 | ID2 | chr2:8822112-8824583 | NT | MOF | OK | 59.204 | 25.7233 | -2.3015709 | -1.2026 | -2.45615 | 5.00E-05 | 0.00368902 | yes |
| XLOC_019144 | XLOC_019144 | FBXO5 | chr6:153291657-153304740 | NT | MOF | OK | 4.71106 | 2.04635 | -2.302177 | -1.203 | -1.78639 | 0.00235 | 0.0685181 | no |
| XLOC_018955 | XLOC_018955 | MB21D1 | chr6:74134855-74162043 | NT | MOF | OK | 2.59509 | 1.12706 | -2.3025305 | -1.2032 | -1.38477 | 0.0245 | 0.296154 | no |
| XLOC_017265 | XLOC_017265 | DCP2 | chr5:112312406-112824527 | NT | MOF | OK | 4.23184 | 1.83426 | -2.3071102 | -1.2061 | -1.44839 | 0.0179 | 0.245846 | no |
| XLOC_015009 | XLOC_015009 | GOLGA4 | chr3:37284681-37408370 | NT | MOF | OK | 30.0791 | 13.0286 | -2.3086978 | -1.2071 | -2.68295 | 5.00E-05 | 0.00368902 | yes |
| XLOC_007429 | XLOC_007429 | MEF2A | chr15:100106132-100256629 | NT | MOF | OK | 17.4028 | 7.53558 | -2.3094175 | -1.2075 | -2.39817 | 5.00E-05 | 0.00368902 | yes |
| XLOC_016974 | XLOC_016974 | FAM198B | chr4:159045731-159094202 | NT | MOF | OK | 13.4618 | 5.8207 | -2.3127459 | -1.2096 | -2.36722 | 5.00E-05 | 0.00368902 | yes |
| XLOC_015660 | XLOC_015660 | LZTFL1 | chr3:45864809-45957216 | NT | MOF | OK | 2.7472 | 1.18413 | -2.3200155 | -1.2141 | -1.77693 | 0.0029 | 0.0786771 | no |
| XLOC_018615 | XLOC_018615 | SERPINB9 | chr6:2887499-2903546 | NT | MOF | OK | 4.26881 | 1.83894 | -2.3213427 | -1.215 | -1.80332 | 0.0033 | 0.0838866 | no |
| XLOC_016911 | XLOC_016911 | NDNF | chr4:121956781-121993673 | NT | MOF | OK | 1.37501 | 0.592088 | -2.3223068 | -1.2156 | -1.35661 | 0.0233 | 0.287978 | no |
| XLOC_016985 | XLOC_016985 | TRIM61 | chr4:165875597-165898818 | NT | MOF | OK | 1.05497 | 0.454094 | -2.3232414 | -1.2161 | -0.932772 | 0.1607 | 0.742164 | no |
| XLOC_003813 | XLOC_003813 | DIXDC1 | chr11:111797867-111893307 | NT | MOF | OK | 13.6013 | 5.85425 | -2.3233207 | -1.2162 | -2.52373 | 5.00E-05 | 0.00368902 | yes |
| XLOC_000643 | XLOC_000643 | ANKRD35 | chr1:145549208-145568526 | NT | MOF | OK | 1.98848 | 0.851264 | -2.3359146 | -1.224 | -1.58774 | 0.00525 | 0.116346 | no |
| XLOC_018406 | XLOC_018406 | PM20D2 | chr6:89855768-89875288 | NT | MOF | OK | 1.401 | 0.597895 | -2.3432208 | -1.2285 | -1.5238 | 0.01175 | 0.191611 | no |
| XLOC_003878 | XLOC_003878 | GRAMD1B | chr11:123396527-123493518 | NT | MOF | OK | 0.906248 | 0.386109 | -2.34713 | -1.2309 | -1.0998 | 0.0635 | 0.49539 | no |
| XLOC_018100 | XLOC_018100 | HIST1H2AE | chr6:26217147-26217711 | NT | MOF | OK | 42.7844 | 18.1833 | -2.3529502 | -1.2345 | -1.97172 | 0.0008 | 0.0310256 | yes |
| XLOC_002594 | XLOC_002594 | HHEX | chr10:94449680-94455408 | NT | MOF | OK | 11.9121 | 5.0594 | -2.3544491 | -1.2354 | -2.06788 | 0.00055 | 0.0234331 | yes |
| XLOC_016702 | XLOC_016702 | RELL1 | chr4:37455551-37687999 | NT | MOF | OK | 15.1814 | 6.44435 | -2.355769 | -1.2362 | -2.5499 | 5.00E-05 | 0.00368902 | yes |
| XLOC_019156 | XLOC_019156 | EZR | chr6:159186772-159240456 | NT | MOF | OK | 47.8332 | 20.3024 | -2.3560367 | -1.2364 | -2.77011 | 5.00E-05 | 0.00368902 | yes |
| XLOC_012403 | XLOC_012403 | TMEM37 | chr2:120189445-120196096 | NT | MOF | OK | 0.77051 | 0.326755 | -2.3580664 | -1.2376 | -1.00197 | 0.0891 | 0.577603 | no |
| XLOC_003398 | XLOC_003398 | CD44 | chr11:35160416-35253949 | NT | MOF | OK | 614.604 | 260.366 | -2.3605386 | -1.2391 | -2.16023 | 0.0005 | 0.0219203 | yes |
| XLOC_013069 | XLOC_013069 | AFF3 | chr2:100163715-100759037 | NT | MOF | OK | 0.728398 | 0.308312 | -2.3625354 | -1.2403 | -1.53237 | 0.00965 | 0.170709 | no |
| XLOC_023535 | XLOC_023535 | GPRASP1 | chrX:101854095-101972661 | NT | MOF | OK | 0.837072 | 0.35372 | -2.366482 | -1.2427 | -0.736554 | 0.2065 | 0.804977 | no |
| XLOC_010308 | XLOC_010308 | OSBPL1A | chr18:21742010-21977833 | NT | MOF | OK | 16.9855 | 7.1725 | -2.3681422 | -1.2438 | -2.56215 | 5.00E-05 | 0.00368902 | yes |
| XLOC_001981 | XLOC_001981 | OLFML2B | chr1:161952981-161993644 | NT | MOF | OK | 2.32896 | 0.980278 | -2.3758158 | -1.2484 | -1.60603 | 0.0103 | 0.178298 | no |
| XLOC_002048 | XLOC_002048 | SEC16B | chr1:177898241-177939050 | NT | MOF | OK | 1.4761 | 0.620498 | -2.3788957 | -1.2503 | -1.57611 | 0.0099 | 0.173357 | no |
| XLOC_001663 | XLOC_001663 | F3 | chr1:94994731-95007413 | NT | MOF | OK | 167.777 | 70.4094 | -2.3828779 | -1.2527 | -2.61777 | 5.00E-05 | 0.00368902 | yes |
| XLOC_006700 | XLOC_006700 | SALL2 | chr14:21989231-22005337 | NT | MOF | OK | 1.71818 | 0.720472 | -2.3847977 | -1.2539 | -1.67402 | 0.003 | 0.079956 | no |
| XLOC_001099 | XLOC_001099 | GALNT2 | chr1:230202955-230417875 | NT | MOF | OK | 127.464 | 53.3937 | -2.3872479 | -1.2553 | -2.78986 | 5.00E-05 | 0.00368902 | yes |
| XLOC_021582 | XLOC_021582 | MCM4 | chr8:48872762-48890719 | NT | MOF | OK | 7.59543 | 3.17628 | -2.3912974 | -1.2578 | -2.45212 | 5.00E-05 | 0.00368902 | yes |

| | | | | | | | | | | | | | | |
|---|---|---|---|---|---|---|---|---|---|---|---|---|---|---|
| XLOC_013348 | XLOC_013348 | PECR | chr2:216903110-216946539 | NT | MOF | OK | 1.45871 | 0.609991 | -2.3913632 | -1.2578 | -1.24735 | 0.0396 | 0.383022 | no |
| XLOC_005440 | XLOC_005440 | RACGAP1 | chr12:50382944-50419307 | NT | MOF | OK | 8.0276 | 3.35615 | -2.3919074 | -1.2582 | -2.18027 | 0.00025 | 0.0132096 | yes |
| XLOC_014186 | XLOC_014186 | ABCG1 | chr21:43619798-43717354 | NT | MOF | OK | 2.93486 | 1.2264 | -2.3930691 | -1.2589 | -1.67654 | 0.0037 | 0.0900805 | no |
| XLOC_016324 | XLOC_016324 | KIAA1211 | chr4:57036360-57196890 | NT | MOF | OK | 1.42036 | 0.59319 | -2.3944436 | -1.2597 | -1.67948 | 0.00355 | 0.0878425 | no |
| XLOC_006049 | XLOC_006049 | SLC46A3 | chr13:29274217-29293150 | NT | MOF | OK | 1.05771 | 0.441494 | -2.3957517 | -1.2605 | -1.36197 | 0.0204 | 0.266278 | no |
| XLOC_010090 | XLOC_010090 | TYMS | chr18:596997-712662 | NT | MOF | OK | 18.8872 | 7.87562 | -2.3981858 | -1.2619 | -1.96054 | 0.001 | 0.0366667 | yes |
| XLOC_001690 | XLOC_001690 | PSRC1 | chr1:109822175-109825790 | NT | MOF | OK | 4.35002 | 1.80846 | -2.4053725 | -1.2663 | -1.72294 | 0.00255 | 0.0719231 | no |
| XLOC_017479 | XLOC_017479 | NEURL1B | chr5:172068275-172118533 | NT | MOF | OK | 1.50864 | 0.626813 | -2.4068422 | -1.2671 | -1.80837 | 0.0019 | 0.0589487 | no |
| XLOC_000557 | XLOC_000557 | GPSM2 | chr1:109419602-109506111 | NT | MOF | OK | 6.11427 | 2.53651 | -2.410505 | -1.2693 | -1.12529 | 0.06375 | 0.495742 | no |
| XLOC_022782 | XLOC_022782 | SMU1 | chr9:33041849-33076714 | NT | MOF | OK | 8.94476 | 3.70474 | -2.4144096 | -1.2717 | -2.67561 | 5.00E-05 | 0.00368902 | yes |
| XLOC_012960 | XLOC_012960 | LOC100133985 | chr2:70351167-70352448 | NT | MOF | OK | 0.830265 | 0.34156 | -2.4308028 | -1.2814 | -0.724857 | 0.2307 | 0.832033 | no |
| XLOC_005407 | XLOC_005407 | SENP1 | chr12:48436680-48540187 | NT | MOF | OK | 4.47003 | 1.83488 | -2.436143 | -1.2846 | -1.09709 | 0.1083 | 0.628504 | no |
| XLOC_000388 | XLOC_000388 | CDKN2C | chr1:51434366-51440309 | NT | MOF | OK | 6.03851 | 2.47482 | -2.4399795 | -1.2869 | -1.73526 | 0.006 | 0.125823 | no |
| XLOC_015441 | XLOC_015441 | SKIL | chr3:170075472-170114637 | NT | MOF | OK | 20.8041 | 8.51062 | -2.444487 | -1.2895 | -2.73439 | 5.00E-05 | 0.00368902 | yes |
| XLOC_022060 | XLOC_022060 | IMPA1 | chr8:82569150-82598589 | NT | MOF | OK | 4.87657 | 1.9926 | -2.4473402 | -1.2912 | -2.10213 | 0.00035 | 0.017008 | yes |
| XLOC_000455 | XLOC_000455 | CTH | chr1:70876900-70905534 | NT | MOF | OK | 1.761 | 0.718678 | -2.4503324 | -1.293 | -1.33876 | 0.0216 | 0.275987 | no |
| XLOC_018126 | XLOC_018126 | HIST1H3H | chr6:27777841-27778314 | NT | MOF | OK | 35.5217 | 14.4795 | -2.4532408 | -1.2947 | -1.76671 | 0.0039 | 0.0934455 | no |
| XLOC_023184 | XLOC_023184 | SAPCD2 | chr9:139956578-139965028 | NT | MOF | OK | 0.745568 | 0.303319 | -2.4580326 | -1.2975 | -1.3554 | 0.02215 | 0.279182 | no |
| XLOC_017054 | XLOC_017054 | TRIP13 | chr5:892968-918164 | NT | MOF | OK | 6.24873 | 2.53987 | -2.4602558 | -1.2988 | -2.01619 | 0.0007 | 0.0283278 | yes |
| XLOC_011152 | XLOC_011152 | CACNG7 | chr19:54415990-54446969 | NT | MOF | OK | 1.37778 | 0.559109 | -2.4642422 | -1.3011 | -1.14377 | 0.04355 | 0.404107 | no |
| XLOC_007118 | XLOC_007118 | SCG5 | chr15:32933869-32989298 | NT | MOF | OK | 9.22836 | 3.72799 | -2.4754251 | -1.3077 | -1.68495 | 0.00565 | 0.121646 | no |
| XLOC_017415 | XLOC_017415 | ARHGEF37 | chr5:148961134-149014527 | NT | MOF | OK | 0.737915 | 0.297386 | -2.4813374 | -1.3111 | -1.44193 | 0.01575 | 0.227416 | no |
| XLOC_007117 | XLOC_007117 | ARHGAP11A | chr15:32907690-32931868 | NT | MOF | OK | 6.95228 | 2.80065 | -2.4823809 | -1.3117 | -2.37236 | 5.00E-05 | 0.00368902 | yes |
| XLOC_007343 | XLOC_007343 | KIAA1199 | chr15:81071711-81243999 | NT | MOF | OK | 189.759 | 76.4415 | -2.4824081 | -1.3117 | -2.65383 | 5.00E-05 | 0.00368902 | yes |
| XLOC_016535 | XLOC_016535 | ARFIP1 | chr4:153701111-153833063 | NT | MOF | OK | 10.5089 | 4.23054 | -2.4840564 | -1.3127 | -2.39488 | 0.0001 | 0.00661202 | yes |
| XLOC_009583 | XLOC_009583 | DHRS13 | chr17:27224798-27230089 | NT | MOF | OK | 0.914473 | 0.367164 | -2.4906391 | -1.3165 | -1.18315 | 0.04195 | 0.395323 | no |
| XLOC_018093 | XLOC_018093 | HIST1H4C | chr6:26104175-26104565 | NT | MOF | OK | 101.525 | 40.7185 | -2.4933384 | -1.3181 | -2.20644 | 0.0003 | 0.0149383 | yes |
| XLOC_013320 | XLOC_013320 | GPR1 | chr2:207040041-207082771 | NT | MOF | OK | 4.61265 | 1.84795 | -2.4960903 | -1.3197 | -1.80668 | 0.003 | 0.079956 | no |
| XLOC_016033 | XLOC_016033 | IFT80 | chr3:159974773-160117320 | NT | MOF | OK | 8.59112 | 3.44006 | -2.497375 | -1.3204 | -2.51617 | 5.00E-05 | 0.00368902 | yes |
| XLOC_009527 | XLOC_009527 | RASD1 | chr17:17397752-17399709 | NT | MOF | OK | 1.92118 | 0.76824 | -2.500755 | -1.3224 | -1.3323 | 0.0206 | 0.267734 | no |
| XLOC_018089 | XLOC_018089 | HIST1H3A | chr6:26020717-26021186 | NT | MOF | OK | 14.3376 | 5.73284 | -2.5009594 | -1.3225 | -1.47604 | 0.01515 | 0.223011 | no |
| XLOC_020577 | XLOC_020577 | PHTF2 | chr7:77428108-77586821 | NT | MOF | OK | 20.5108 | 8.19228 | -2.5036742 | -1.3241 | -2.58585 | 5.00E-05 | 0.00368902 | yes |
| XLOC_010414 | XLOC_010414 | MBP | chr18:74690788-74844774 | NT | MOF | OK | 2.61817 | 1.04371 | -2.5085225 | -1.3268 | -1.91053 | 0.0005 | 0.0219203 | yes |
| XLOC_010576 | XLOC_010576 | ATG4D | chr19:10654646-10676702 | NT | MOF | OK | 9.0326 | 3.57365 | -2.5275559 | -1.3377 | -1.6081 | 0.00695 | 0.138771 | no |
| XLOC_021167 | XLOC_021167 | SGCE | chr7:94214535-94285521 | NT | MOF | OK | 21.0716 | 8.33311 | -2.5286598 | -1.3384 | -2.51256 | 5.00E-05 | 0.00368902 | yes |
| XLOC_000259 | XLOC_000259 | TSSK3 | chr1:32827861-32829924 | NT | MOF | OK | 1.00017 | 0.395424 | -2.5293609 | -1.3388 | -1.08811 | 0.06585 | 0.502387 | no |
| XLOC_016037 | XLOC_016037 | B3GALNT1 | chr3:160801670-160823160 | NT | MOF | OK | 2.23133 | 0.881696 | -2.5307249 | -1.3396 | -1.72545 | 0.00335 | 0.0844479 | no |
| XLOC_008480 | XLOC_008480 | IL21R-AS1 | chr16:27413482-27464714 | NT | MOF | OK | 0.840712 | 0.332161 | -2.5310377 | -1.3397 | -0.806682 | 0.1893 | 0.784183 | no |
| XLOC_022580 | XLOC_022580 | DNM1 | chr9:130928343-131017527 | NT | MOF | OK | 5.96109 | 2.35445 | -2.5318397 | -1.3402 | -0.631952 | 0.41375 | 0.958788 | no |
| XLOC_009891 | XLOC_009891 | BRIP1 | chr17:59756546-59940920 | NT | MOF | OK | 1.61206 | 0.636141 | -2.5341237 | -1.3415 | -2.0708 | 0.0005 | 0.0219203 | yes |
| XLOC_007271 | XLOC_007271 | GLCE | chr15:69116302-69564544 | NT | MOF | OK | 18.1094 | 7.13661 | -2.5375353 | -1.3434 | -2.83058 | 5.00E-05 | 0.00368902 | yes |
| XLOC_021184 | XLOC_021184 | TMEM130 | chr7:98444110-98467673 | NT | MOF | OK | 4.06232 | 1.5997 | -2.5394261 | -1.3445 | -1.7241 | 0.00275 | 0.0759703 | no |

| | | | | | | | | | | | | | | |
|---|---|---|---|---|---|---|---|---|---|---|---|---|---|---|
| XLOC_002644 | XLOC_002644 | SEMA4G | chr10:102732285-102747272 | NT | MOF | OK | 0.844104 | 0.332307 | -2.5401331 | -1.3449 | -0.69219 | 0.29795 | 0.896447 | no |
| XLOC_005641 | XLOC_005641 | TMPO-AS1 | chr12:98906750-98944157 | NT | MOF | OK | 0.911499 | 0.356194 | -2.5589959 | -1.3556 | -0.653775 | 0.48745 | 0.989872 | no |
| XLOC_000869 | XLOC_000869 | C1orf112 | chr1:169764549-169863100 | NT | MOF | OK | 1.09046 | 0.426071 | -2.5593387 | -1.3558 | -1.00198 | 0.09905 | 0.605 | no |
| XLOC_004650 | XLOC_004650 | LOC100507424 | chr12:2945981-2998691 | NT | MOF | OK | 1.51822 | 0.591688 | -2.5659131 | -1.3595 | -0.369786 | 0.5779 | 0.999021 | no |
| XLOC_018117 | XLOC_018117 | HIST1H4I | chr6:27106071-27114637 | NT | MOF | OK | 11.4298 | 4.45042 | -2.568252 | -1.3608 | -0.335178 | 0.5255 | 0.999021 | no |
| XLOC_023525 | XLOC_023525 | CENPI | chrX:100354797-100417978 | NT | MOF | OK | 1.765 | 0.687029 | -2.5690327 | -1.3612 | -1.53481 | 0.014 | 0.212814 | no |
| XLOC_001679 | XLOC_001679 | COL11A1 | chr1:103342022-103574052 | NT | MOF | OK | 3.23185 | 1.25587 | -2.5733953 | -1.3637 | -2.37875 | 0.00015 | 0.00902985 | yes |
| XLOC_001514 | XLOC_001514 | TESK2 | chr1:45794913-45956840 | NT | MOF | OK | 1.06499 | 0.413614 | -2.5748403 | -1.3645 | -0.866249 | 0.13745 | 0.692688 | no |
| XLOC_018701 | XLOC_018701 | HIST1H3I | chr6:27839622-27840099 | NT | MOF | OK | 35.0978 | 13.5984 | -2.5810242 | -1.3679 | -1.88044 | 0.0017 | 0.054418 | no |
| XLOC_011427 | XLOC_011427 | SPC24 | chr19:11257830-11266484 | NT | MOF | OK | 4.56888 | 1.76797 | -2.584252 | -1.3698 | -1.28045 | 0.03595 | 0.363708 | no |
| XLOC_004494 | XLOC_004494 | PDGFD | chr11:103777913-104035027 | NT | MOF | OK | 4.36532 | 1.68775 | -2.5864731 | -1.371 | -2.01854 | 0.0012 | 0.0422093 | yes |
| XLOC_004450 | XLOC_004450 | NOX4 | chr11:89057521-89322779 | NT | MOF | OK | 0.81267 | 0.313441 | -2.5927368 | -1.3745 | -1.34305 | 0.01075 | 0.183204 | no |
| XLOC_022529 | XLOC_022529 | CNTRL | chr9:123850573-123939886 | NT | MOF | OK | 0.905303 | 0.349117 | -2.5931221 | -1.3747 | -1.76672 | 0.00535 | 0.1177 | no |
| XLOC_001495 | XLOC_001495 | LEPRE1 | chr1:43212005-43232755 | NT | MOF | OK | 68.5034 | 26.3964 | -2.5951796 | -1.3758 | -3.08105 | 5.00E-05 | 0.00368902 | yes |
| XLOC_017160 | XLOC_017160 | CENPH | chr5:68485374-68506184 | NT | MOF | OK | 3.44061 | 1.32508 | -2.59653 | -1.3766 | -1.55952 | 0.01345 | 0.207055 | no |
| XLOC_013884 | XLOC_013884 | NAPB | chr20:23355155-23402156 | NT | MOF | OK | 3.62228 | 1.38831 | -2.6091291 | -1.3836 | -2.14922 | 0.00025 | 0.0132096 | yes |
| XLOC_006207 | XLOC_006207 | KDELC1 | chr13:103436630-103528351 | NT | MOF | OK | 11.9519 | 4.57692 | -2.6113413 | -1.3848 | -0.986991 | 0.1058 | 0.621137 | no |
| XLOC_013973 | XLOC_013973 | JPH2 | chr20:42740336-42816218 | NT | MOF | OK | 1.29838 | 0.49707 | -2.6120667 | -1.3852 | -1.6138 | 0.00505 | 0.11274 | no |
| XLOC_018101 | XLOC_018101 | HIST1H3E | chr6:26225382-26225844 | NT | MOF | OK | 9.60461 | 3.66987 | -2.6171527 | -1.388 | -1.35701 | 0.02655 | 0.309779 | no |
| XLOC_022245 | XLOC_022245 | RECQL4 | chr8:145736666-145743210 | NT | MOF | OK | 2.28643 | 0.869958 | -2.6282073 | -1.3941 | -1.89141 | 0.00165 | 0.0535255 | no |
| XLOC_017484 | XLOC_017484 | CREBRF | chr5:172483354-172566291 | NT | MOF | OK | 5.83285 | 2.21488 | -2.6334835 | -1.397 | -2.50827 | 0.00015 | 0.00902985 | yes |
| XLOC_018674 | XLOC_018674 | HIST1H2AB | chr6:26033319-26033796 | NT | MOF | OK | 12.0562 | 4.57485 | -2.6353214 | -1.398 | -1.49167 | 0.0133 | 0.206056 | no |
| XLOC_004120 | XLOC_004120 | LIN7C | chr11:27515964-27528326 | NT | MOF | OK | 31.1902 | 11.8274 | -2.6371138 | -1.399 | -3.05374 | 5.00E-05 | 0.00368902 | yes |
| XLOC_017320 | XLOC_017320 | UBE2B | chr5:133706869-133727799 | NT | MOF | OK | 18.8713 | 7.1457 | -2.6409309 | -1.4011 | -2.81625 | 5.00E-05 | 0.00368902 | yes |
| XLOC_001398 | XLOC_001398 | SNORA16A | chr1:28879528-28908366 | NT | MOF | OK | 42.0708 | 15.9269 | -2.6414933 | -1.4014 | -5.03709 | 0.53875 | 0.999021 | no |
| XLOC_015680 | XLOC_015680 | CDC25A | chr3:48198667-48229801 | NT | MOF | OK | 2.11974 | 0.802387 | -2.6417926 | -1.4015 | -1.87009 | 0.0023 | 0.0677129 | no |
| XLOC_001642 | XLOC_001642 | GBP2 | chr1:89573309-89591799 | NT | MOF | OK | 11.4828 | 4.34508 | -2.6427131 | -1.402 | -2.51299 | 5.00E-05 | 0.00368902 | yes |
| XLOC_017431 | XLOC_017431 | GRIA1 | chr5:152870083-153193429 | NT | MOF | OK | 0.741449 | 0.280317 | -2.6450376 | -1.4033 | -1.43853 | 0.0062 | 0.128239 | no |
| XLOC_018102 | XLOC_018102 | HIST1H4F | chr6:26240653-26241021 | NT | MOF | OK | 22.3105 | 8.42594 | -2.6478351 | -1.4048 | -1.45486 | 0.01775 | 0.245738 | no |
| XLOC_002895 | XLOC_002895 | CXCL12 | chr10:44865604-44880545 | NT | MOF | OK | 75.7362 | 28.5654 | -2.6513264 | -1.4067 | -2.71228 | 5.00E-05 | 0.00368902 | yes |
| XLOC_004127 | XLOC_004127 | IMMP1L | chr11:31391376-31531169 | NT | MOF | OK | 1.02732 | 0.387109 | -2.6538262 | -1.4081 | -0.404807 | 0.59165 | 0.999021 | no |
| XLOC_007269 | XLOC_007269 | NOX5,SPESP1 | chr15:69116302-69564544 | NT | MOF | OK | 1.6873 | 0.634875 | -2.6576885 | -1.4102 | -0.46911 | 0.42735 | 0.966147 | no |
| XLOC_007597 | XLOC_007597 | MNS1 | chr15:56657643-56757335 | NT | MOF | OK | 0.765797 | 0.288089 | -2.6581959 | -1.4105 | -0.874301 | 0.1393 | 0.697653 | no |
| XLOC_007878 | XLOC_007878 | C16orf59 | chr16:2510114-2514964 | NT | MOF | OK | 1.28366 | 0.482706 | -2.6592999 | -1.4111 | -1.31636 | 0.0347 | 0.357336 | no |
| XLOC_001793 | XLOC_001793 | SV2A | chr1:149874871-149889434 | NT | MOF | OK | 5.56026 | 2.07972 | -2.6735618 | -1.4188 | -2.44906 | 5.00E-05 | 0.00368902 | yes |
| XLOC_017436 | XLOC_017436 | CNOT8 | chr5:154238197-154256352 | NT | MOF | OK | 17.0697 | 6.37654 | -2.6769533 | -1.4206 | -2.79724 | 5.00E-05 | 0.00368902 | yes |
| XLOC_008859 | XLOC_008859 | MYOCD | chr17:12569206-12670651 | NT | MOF | OK | 2.8305 | 1.05404 | -2.685382 | -1.4251 | -1.71091 | 0.001 | 0.0366667 | yes |
| XLOC_016454 | XLOC_016454 | CYP2U1 | chr4:108852716-108874613 | NT | MOF | OK | 6.26027 | 2.32959 | -2.687284 | -1.4262 | -2.62272 | 5.00E-05 | 0.00368902 | yes |
| XLOC_012224 | XLOC_012224 | MXD1 | chr2:70142172-70170076 | NT | MOF | OK | 6.66048 | 2.46475 | -2.7022944 | -1.4342 | -2.7133 | 5.00E-05 | 0.00368902 | yes |
| XLOC_004570 | XLOC_004570 | MCAM | chr11:119179233-119187840 | NT | MOF | OK | 3.00014 | 1.10818 | -2.7072678 | -1.4368 | -2.00284 | 0.00075 | 0.0295603 | yes |
| XLOC_007604 | XLOC_007604 | MYO1E | chr15:59428167-59665071 | NT | MOF | OK | 12.5358 | 4.62965 | -2.7077209 | -1.4371 | -2.84164 | 5.00E-05 | 0.00368902 | yes |
| XLOC_023434 | XLOC_023434 | TRO | chrX:54946995-54957866 | NT | MOF | OK | 5.19084 | 1.91386 | -2.712236 | -1.4395 | -2.07126 | 0.00025 | 0.0132096 | yes |

| | | | | | | | | | | | | | | |
|---|---|---|---|---|---|---|---|---|---|---|---|---|---|---|
| XLOC_018960 | XLOC_018960 | TMEM30A | chr6:75962637-75994632 | NT | MOF | OK | 114.36 | 42.1459 | -2.7134312 | -1.4401 | -3.22311 | 5.00E-05 | 0.00368902 | yes |
| XLOC_007217 | XLOC_007217 | MAPK6 | chr15:52311410-52358462 | NT | MOF | OK | 27.55 | 10.147 | -2.7150882 | -1.441 | -3.15302 | 5.00E-05 | 0.00368902 | yes |
| XLOC_003547 | XLOC_003547 | FEN1 | chr11:61560108-61564714 | NT | MOF | OK | 8.17778 | 3.00036 | -2.7255996 | -1.4466 | -2.27825 | 0.0002 | 0.0112558 | yes |
| XLOC_021687 | XLOC_021687 | MATN2 | chr8:98881310-99048946 | NT | MOF | OK | 11.1734 | 4.09031 | -2.7316756 | -1.4498 | -2.63503 | 5.00E-05 | 0.00368902 | yes |
| XLOC_012577 | XLOC_012577 | SGOL2 | chr2:201390864-201448818 | NT | MOF | OK | 3.67105 | 1.34122 | -2.7370976 | -1.4526 | -2.23711 | 0.00035 | 0.017008 | yes |
| XLOC_014144 | XLOC_014144 | IFNAR1 | chr21:34697213-34732128 | NT | MOF | OK | 13.1321 | 4.79265 | -2.7400499 | -1.4542 | -3.11394 | 5.00E-05 | 0.00368902 | yes |
| XLOC_003947 | XLOC_003947 | CEND1 | chr11:787109-790126 | NT | MOF | OK | 5.71719 | 2.08398 | -2.7433996 | -1.456 | -1.829 | 0.0019 | 0.0589487 | no |
| XLOC_012470 | XLOC_012470 | ARL6IP6 | chr2:153574406-153617767 | NT | MOF | OK | 6.06381 | 2.20918 | -2.7448239 | -1.4567 | -2.25566 | 0.0005 | 0.0219203 | yes |
| XLOC_001552 | XLOC_001552 | SLC1A7 | chr1:53552854-53608289 | NT | MOF | OK | 0.968915 | 0.35096 | -2.7607562 | -1.4651 | -1.31953 | 0.03485 | 0.357686 | no |
| XLOC_014385 | XLOC_014385 | C21orf58 | chr21:47721046-47743785 | NT | MOF | OK | 1.30113 | 0.470449 | -2.7657196 | -1.4677 | -1.61778 | 0.0077 | 0.148124 | no |
| XLOC_014369 | XLOC_014369 | SUMO3 | chr21:46225531-46238044 | NT | MOF | OK | 43.9181 | 15.8785 | -2.7658847 | -1.4677 | -3.09478 | 5.00E-05 | 0.00368902 | yes |
| XLOC_021420 | XLOC_021420 | NCAPG2 | chr7:158424002-158497520 | NT | MOF | OK | 5.95727 | 2.15185 | -2.7684411 | -1.4691 | -2.51403 | 0.0001 | 0.00661202 | yes |
| XLOC_003896 | XLOC_003896 | PKNOX2 | chr11:125034558-125303285 | NT | MOF | OK | 2.23456 | 0.804813 | -2.7764959 | -1.4733 | -1.64992 | 0.00625 | 0.128833 | no |
| XLOC_008085 | XLOC_008085 | ORC6 | chr16:46723557-46732306 | NT | MOF | OK | 3.91386 | 1.40864 | -2.7784672 | -1.4743 | -1.83075 | 0.00275 | 0.0759703 | no |
| XLOC_005654 | XLOC_005654 | IGF1 | chr12:102789644-102874378 | NT | MOF | OK | 2.28853 | 0.822643 | -2.7819236 | -1.4761 | -1.04038 | 0.03535 | 0.359743 | no |
| XLOC_001533 | XLOC_001533 | STIL | chr1:47715810-47779819 | NT | MOF | OK | 2.32976 | 0.836924 | -2.7837175 | -1.477 | -2.20156 | 0.0005 | 0.0219203 | yes |
| XLOC_005454 | XLOC_005454 | KRT80 | chr12:52562779-52585784 | NT | MOF | OK | 1.57191 | 0.563528 | -2.7894089 | -1.48 | -1.8072 | 0.00375 | 0.0903884 | no |
| XLOC_017452 | XLOC_017452 | PTTG1 | chr5:159848864-159855746 | NT | MOF | OK | 17.5124 | 6.27596 | -2.7903938 | -1.4805 | -2.01874 | 0.00125 | 0.0434626 | yes |
| XLOC_002206 | XLOC_002206 | LBR | chr1:225589203-225616557 | NT | MOF | OK | 8.43229 | 3.01453 | -2.7972155 | -1.484 | -2.78784 | 5.00E-05 | 0.00368902 | yes |
| XLOC_009971 | XLOC_009971 | H3F3B | chr17:73772514-73775860 | NT | MOF | OK | 115.104 | 41.0963 | -2.8008361 | -1.4859 | -3.35579 | 5.00E-05 | 0.00368902 | yes |
| XLOC_014037 | XLOC_014037 | AURKA | chr20:54944444-54967351 | NT | MOF | OK | 12.9179 | 4.59602 | -2.810671 | -1.4909 | -2.59403 | 5.00E-05 | 0.00368902 | yes |
| XLOC_006792 | XLOC_006792 | POLE2 | chr14:50110269-50155098 | NT | MOF | OK | 0.900255 | 0.319534 | -2.8173997 | -1.4944 | -1.23354 | 0.01955 | 0.260237 | no |
| XLOC_008836 | XLOC_008836 | SCARNA21 | chr17:7788122-7816075 | NT | MOF | OK | 95.4911 | 33.8296 | -2.8227085 | -1.4971 | -0.097654 | 0.43745 | 0.970151 | no |
| XLOC_004789 | XLOC_004789 | DDX11 | chr12:31173696-31257725 | NT | MOF | OK | 1.55165 | 0.549311 | -2.8247204 | -1.4981 | -1.68126 | 0.00605 | 0.126434 | no |
| XLOC_016998 | XLOC_016998 | HMGB2 | chr4:174252526-174255595 | NT | MOF | OK | 19.2783 | 6.79132 | -2.8386676 | -1.5052 | -2.62873 | 5.00E-05 | 0.00368902 | yes |
| XLOC_007391 | XLOC_007391 | TICRR | chr15:90118817-90198682 | NT | MOF | OK | 0.853796 | 0.299966 | -2.8463092 | -1.5091 | -0.85757 | 0.1333 | 0.684025 | no |
| XLOC_005920 | XLOC_005920 | DIAPH3-AS1 | chr13:60239720-60738119 | NT | MOF | OK | 1.05608 | 0.369854 | -2.855397 | -1.5137 | -0.2353 | 0.3064 | 0.902712 | no |
| XLOC_012439 | XLOC_012439 | CCDC74A | chr2:132202795-132360468 | NT | MOF | OK | 2.28777 | 0.796963 | -2.8706101 | -1.5214 | -0.697845 | 0.2785 | 0.885853 | no |
| XLOC_014293 | XLOC_014293 | SNORA80 | chr21:33664123-33765312 | NT | MOF | OK | 93.9503 | 32.5956 | -2.8823001 | -1.5272 | -0.265986 | 0.4521 | 0.975639 | no |
| XLOC_016007 | XLOC_016007 | GPR87 | chr3:150804675-151176497 | NT | MOF | OK | 0.901043 | 0.312434 | -2.8839467 | -1.5281 | -0.405487 | 0.6172 | 0.999021 | no |
| XLOC_020477 | XLOC_020477 | C7orf69 | chr7:47814249-47988071 | NT | MOF | OK | 0.834583 | 0.288672 | -2.8911117 | -1.5316 | -0.540174 | 0.3673 | 0.940201 | no |
| XLOC_011514 | XLOC_011514 | HAUS8 | chr19:17160570-17186343 | NT | MOF | OK | 2.73432 | 0.94542 | -2.8921749 | -1.5322 | -1.63182 | 0.0078 | 0.148864 | no |
| XLOC_012805 | XLOC_012805 | MATN3 | chr2:20191812-20212455 | NT | MOF | OK | 1.4151 | 0.487976 | -2.8999377 | -1.536 | -1.64678 | 0.00655 | 0.132978 | no |
| XLOC_013917 | XLOC_013917 | TSPY26P | chr20:30776948-30778163 | NT | MOF | OK | 1.23145 | 0.422402 | -2.9153508 | -1.5437 | -1.30347 | 0.02965 | 0.329395 | no |
| XLOC_018118 | XLOC_018118 | HIST1H2AH | chr6:27114860-27115341 | NT | MOF | OK | 35.7822 | 12.1971 | -2.9336646 | -1.5527 | -2.12554 | 0.00025 | 0.0132096 | yes |
| XLOC_017449 | XLOC_017449 | ADRA1B | chr5:159343739-159400017 | NT | MOF | OK | 1.34199 | 0.455438 | -2.9465921 | -1.5591 | -1.62641 | 0.00635 | 0.13045 | no |
| XLOC_021576 | XLOC_021576 | SGK196 | chr8:42948656-42978323 | NT | MOF | OK | 11.5105 | 3.89748 | -2.9533186 | -1.5623 | -2.41519 | 0.0001 | 0.00661202 | yes |
| XLOC_018573 | XLOC_018573 | TMEM181 | chr6:158957467-159056467 | NT | MOF | OK | 18.0928 | 6.11054 | -2.9609167 | -1.5661 | -3.40796 | 5.00E-05 | 0.00368902 | yes |
| XLOC_022262 | XLOC_022262 | VLDLR | chr9:2535654-2654485 | NT | MOF | OK | 11.6582 | 3.93594 | -2.9619862 | -1.5666 | -2.98448 | 5.00E-05 | 0.00368902 | yes |
| XLOC_005438 | XLOC_005438 | FAIM2 | chr12:50260678-50297760 | NT | MOF | OK | 2.30135 | 0.773622 | -2.9747732 | -1.5728 | -1.76959 | 0.0035 | 0.0871399 | no |
| XLOC_022883 | XLOC_022883 | ALDH1A1 | chr9:75515577-75568233 | NT | MOF | OK | 20.4077 | 6.81223 | -2.9957444 | -1.5829 | -2.87441 | 5.00E-05 | 0.00368902 | yes |
| XLOC_012587 | XLOC_012587 | CDK15 | chr2:202671151-202760273 | NT | MOF | OK | 3.57514 | 1.19304 | -2.996664 | -1.5834 | -2.12593 | 0.00065 | 0.0267517 | yes |

| | | | | | | | | | | | | | |
|---|---|---|---|---|---|---|---|---|---|---|---|---|---|
| XLOC_001785 | XLOC_001785 | HIST2H2BF | chr1:149754244-149783928 | NT | MOF | OK | 39.6643 | 13.2041 | -3.0039382 | -1.5869 | -2.25324 | 0.00025 | 0.0132096 yes |
| XLOC_012724 | XLOC_012724 | CXCR7 | chr2:237478379-237490994 | NT | MOF | OK | 0.771234 | 0.255094 | -3.0233326 | -1.5961 | -1.37875 | 0.02135 | 0.27366 no |
| XLOC_009197 | XLOC_009197 | PRR11 | chr17:57232859-57284070 | NT | MOF | OK | 4.66903 | 1.54373 | -3.0245121 | -1.5967 | -2.83163 | 5.00E-05 | 0.00368902 yes |
| XLOC_006161 | XLOC_006161 | EDNRB | chr13:78469615-78549674 | NT | MOF | OK | 2.30049 | 0.756622 | -3.0404746 | -1.6043 | -1.54286 | 0.00365 | 0.0892222 no |
| XLOC_023540 | XLOC_023540 | TCEAL7 | chrX:102585113-102587251 | NT | MOF | OK | 5.42644 | 1.78346 | -3.0426474 | -1.6053 | -1.83557 | 0.00315 | 0.0819677 no |
| XLOC_013926 | XLOC_013926 | E2F1 | chr20:32263291-32274210 | NT | MOF | OK | 3.78026 | 1.23706 | -3.0558421 | -1.6116 | -2.19073 | 0.00055 | 0.0234331 yes |
| XLOC_002112 | XLOC_002112 | UBE2T | chr1:202300784-202311094 | NT | MOF | OK | 5.47736 | 1.77845 | -3.0798504 | -1.6229 | -1.78406 | 0.0034 | 0.0848247 no |
| XLOC_002943 | XLOC_002943 | ZWINT | chr10:58117198-58121034 | NT | MOF | OK | 8.24612 | 2.67482 | -3.0828691 | -1.6243 | -2.3764 | 0.00045 | 0.0205472 yes |
| XLOC_022019 | XLOC_022019 | MYBL1 | chr8:67474409-67525480 | NT | MOF | OK | 8.8525 | 2.86373 | -3.0912481 | -1.6282 | -2.84049 | 5.00E-05 | 0.00368902 yes |
| XLOC_024323 | XLOC_024323 | TTTY14 | chrY:21094584-21239302 | NT | MOF | OK | 1.90335 | 0.61509 | -3.0944252 | -1.6297 | -0.991298 | 0.30285 | 0.90059 no |
| XLOC_015392 | XLOC_015392 | LOC646903 | chr3:149689065-149691029 | NT | MOF | OK | 0.859754 | 0.274964 | -3.1267875 | -1.6447 | -1.38089 | 0.0214 | 0.274011 no |
| XLOC_013568 | XLOC_013568 | GINS1 | chr20:25388322-25429191 | NT | MOF | OK | 1.5247 | 0.486107 | -3.1365522 | -1.6492 | -1.8442 | 0.00355 | 0.0878425 no |
| XLOC_016930 | XLOC_016930 | SLC7A11 | chr4:138948576-139163503 | NT | MOF | OK | 24.3094 | 7.73134 | -3.1442674 | -1.6527 | -3.64806 | 5.00E-05 | 0.00368902 yes |
| XLOC_000528 | XLOC_000528 | LPPR4 | chr1:99729847-99775138 | NT | MOF | OK | 1.85298 | 0.585195 | -3.1664317 | -1.6629 | -2.29357 | 0.00035 | 0.017008 yes |
| XLOC_016909 | XLOC_016909 | MAD2L1 | chr4:120980578-120988013 | NT | MOF | OK | 4.59899 | 1.45238 | -3.1665198 | -1.6629 | -2.01991 | 0.0009 | 0.0342453 yes |
| XLOC_003732 | XLOC_003732 | C11orf82 | chr11:82612736-82645699 | NT | MOF | OK | 1.21703 | 0.383463 | -3.1737873 | -1.6662 | -1.83349 | 0.0031 | 0.0815435 no |
| XLOC_007273 | XLOC_007273 | KIF23 | chr15:69706626-69740764 | NT | MOF | OK | 10.2585 | 3.22063 | -3.1852464 | -1.6714 | -2.97299 | 5.00E-05 | 0.00368902 yes |
| XLOC_012903 | XLOC_012903 | CALM2 | chr2:47387220-47403740 | NT | MOF | OK | 539.744 | 169.209 | -3.1898067 | -1.6735 | -3.60024 | 5.00E-05 | 0.00368902 yes |
| XLOC_020953 | XLOC_020953 | CDCA7L | chr7:21582832-21985542 | NT | MOF | OK | 5.62281 | 1.76162 | -3.1918405 | -1.6744 | -2.43815 | 5.00E-05 | 0.00368902 yes |
| XLOC_016916 | XLOC_016916 | CCNA2 | chr4:122722471-122745088 | NT | MOF | OK | 7.29066 | 2.27062 | -3.2108675 | -1.683 | -1.82159 | 0.00445 | 0.102367 no |
| XLOC_013582 | XLOC_013582 | TPX2 | chr20:30326903-30389603 | NT | MOF | OK | 11.8284 | 3.65132 | -3.239486 | -1.6958 | -3.1143 | 5.00E-05 | 0.00368902 yes |
| XLOC_014952 | XLOC_014952 | CAND2 | chr3:12838170-12876313 | NT | MOF | OK | 1.36377 | 0.420436 | -3.2437042 | -1.6976 | -1.98833 | 0.00285 | 0.0774944 no |
| XLOC_008067 | XLOC_008067 | KAT8 | chr16:31128984-31142714 | NT | MOF | OK | 7.22806 | 2.21345 | -3.2655176 | -1.7073 | -2.30738 | 0.0005 | 0.0219203 yes |
| XLOC_000431 | XLOC_000431 | EFCAB7 | chr1:63989012-64038364 | NT | MOF | OK | 1.22235 | 0.371699 | -3.288548 | -1.7175 | -1.61206 | 0.016 | 0.229112 no |
| XLOC_013653 | XLOC_013653 | FAM83D | chr20:37554954-37581703 | NT | MOF | OK | 5.55562 | 1.68834 | -3.2905813 | -1.7184 | -2.44355 | 0.0001 | 0.00661202 yes |
| XLOC_007780 | XLOC_007780 | PRC1 | chr15:91509267-91537881 | NT | MOF | OK | 8.41526 | 2.5541 | -3.2948044 | -1.7202 | -2.83287 | 5.00E-05 | 0.00368902 yes |
| XLOC_004766 | XLOC_004766 | GOLT1B | chr12:21654698-21671337 | NT | MOF | OK | 21.4765 | 6.48637 | -3.3110199 | -1.7273 | -3.46705 | 5.00E-05 | 0.00368902 yes |
| XLOC_017159 | XLOC_017159 | CCNB1 | chr5:68462836-68474070 | NT | MOF | OK | 11.0834 | 3.34452 | -3.3138986 | -1.7285 | -2.84835 | 5.00E-05 | 0.00368902 yes |
| XLOC_005729 | XLOC_005729 | CIT | chr12:120123594-120315095 | NT | MOF | OK | 2.72178 | 0.818722 | -3.3244251 | -1.7331 | -1.10331 | 0.0046 | 0.104624 no |
| XLOC_022472 | XLOC_022472 | TGFBR1 | chr9:101867411-101916473 | NT | MOF | OK | 16.6857 | 5.01725 | -3.3256665 | -1.7337 | -3.61041 | 5.00E-05 | 0.00368902 yes |
| XLOC_008284 | XLOC_008284 | CDT1 | chr16:88870185-88875666 | NT | MOF | OK | 2.99535 | 0.89564 | -3.3443683 | -1.7417 | -2.19718 | 0.00065 | 0.0267517 yes |
| XLOC_018098 | XLOC_018098 | HIST1H2BF | chr6:26199786-26200216 | NT | MOF | OK | 19.7362 | 5.87274 | -3.360646 | -1.7487 | -1.824 | 0.006 | 0.125823 no |
| XLOC_006920 | XLOC_006920 | GALC | chr14:88399357-88460009 | NT | MOF | OK | 7.28629 | 2.16302 | -3.3685726 | -1.7521 | -3.05183 | 5.00E-05 | 0.00368902 yes |
| XLOC_021704 | XLOC_021704 | FZD6 | chr8:104310660-104345094 | NT | MOF | OK | 23.5357 | 6.89792 | -3.4119996 | -1.7706 | -3.47827 | 5.00E-05 | 0.00368902 yes |
| XLOC_012285 | XLOC_012285 | RMND5A | chr2:86730552-87005164 | NT | MOF | OK | 6.12969 | 1.79389 | -3.4169821 | -1.7727 | -1.15173 | 0.05525 | 0.462647 no |
| XLOC_009045 | XLOC_009045 | CDC6 | chr17:38444145-38459413 | NT | MOF | OK | 3.40874 | 0.992462 | -3.4346302 | -1.7802 | -2.37676 | 5.00E-05 | 0.00368902 yes |
| XLOC_017575 | XLOC_017575 | FAM173B | chr5:10225619-10250021 | NT | MOF | OK | 3.68594 | 1.06972 | -3.4457054 | -1.7848 | -2.36879 | 0.0001 | 0.00661202 yes |
| XLOC_015585 | XLOC_015585 | FGD5-AS1 | chr3:14984285-15106816 | NT | MOF | OK | 25.6876 | 7.43993 | -3.4526669 | -1.7877 | -2.31168 | 0.00025 | 0.0132096 yes |
| XLOC_014160 | XLOC_014160 | CHAF1B | chr21:37757688-37789125 | NT | MOF | OK | 1.85581 | 0.536619 | -3.4583382 | -1.7901 | -1.91388 | 0.0015 | 0.0495902 yes |
| XLOC_001075 | XLOC_001075 | SRP9 | chr1:225965514-225978168 | NT | MOF | OK | 50.5931 | 14.6204 | -3.4604457 | -1.791 | -3.71068 | 5.00E-05 | 0.00368902 yes |
| XLOC_022470 | XLOC_022470 | GALNT12 | chr9:101569980-101612363 | NT | MOF | OK | 3.497 | 1.00895 | -3.4659795 | -1.7933 | -2.324 | 0.0004 | 0.0189063 yes |
| XLOC_007622 | XLOC_007622 | KIAA0101 | chr15:64657210-64673702 | NT | MOF | OK | 4.05431 | 1.16504 | -3.4799749 | -1.7991 | -2.08134 | 0.00225 | 0.0668919 no |

| | | | | | | | | | | | | | | |
|---|---|---|---|---|---|---|---|---|---|---|---|---|---|---|
| XLOC_021542 | XLOC_021542 | MAK16 | chr8:33342684-33370703 | NT | MOF | OK | 13.397 | 3.82847 | -3.4993091 | -1.8071 | -2.88737 | 5.00E-05 | 0.00368902 | yes |
| XLOC_018097 | XLOC_018097 | HIST1H2BE | chr6:26184023-26184458 | NT | MOF | OK | 19.5465 | 5.56777 | -3.5106515 | -1.8117 | -2.01227 | 0.00135 | 0.0457563 | yes |
| XLOC_017844 | XLOC_017844 | SPATA24 | chr5:138732455-138739776 | NT | MOF | OK | 1.70188 | 0.484533 | -3.512413 | -1.8125 | -1.2529 | 0.04575 | 0.417163 | no |
| XLOC_014938 | XLOC_014938 | FANCD2 | chr3:10068112-10149915 | NT | MOF | OK | 2.03475 | 0.573044 | -3.5507745 | -1.8281 | -2.45162 | 0.00015 | 0.00902985 | yes |
| XLOC_005121 | XLOC_005121 | RNFT2 | chr12:117176095-117291436 | NT | MOF | OK | 0.745164 | 0.209354 | -3.5593492 | -1.8316 | -1.59545 | 0.0057 | 0.122504 | no |
| XLOC_007147 | XLOC_007147 | GCHFR | chr15:41056284-41059911 | NT | MOF | OK | 1.23211 | 0.343454 | -3.5874091 | -1.8429 | -1.25652 | 0.05055 | 0.442907 | no |
| XLOC_010385 | XLOC_010385 | LMAN1 | chr18:56995055-57026508 | NT | MOF | OK | 67.3058 | 18.7561 | -3.5884752 | -1.8434 | -4.08536 | 5.00E-05 | 0.00368902 | yes |
| XLOC_008908 | XLOC_008908 | RNF112 | chr17:19314490-19320589 | NT | MOF | OK | 2.64406 | 0.73569 | -3.5939866 | -1.8456 | -2.32414 | 0.0003 | 0.0149383 | yes |
| XLOC_002959 | XLOC_002959 | DNAJC12 | chr10:69556426-69597937 | NT | MOF | OK | 1.22327 | 0.340152 | -3.5962452 | -1.8465 | -1.27162 | 0.03 | 0.331204 | no |
| XLOC_004034 | XLOC_004034 | TAF10 | chr11:6624963-6633475 | NT | MOF | OK | 89.0368 | 24.7437 | -3.5983624 | -1.8473 | -1.28551 | 0.0254 | 0.301019 | no |
| XLOC_018116 | XLOC_018116 | HIST1H2AG | chr6:27100816-27101314 | NT | MOF | OK | 42.6834 | 11.7403 | -3.6356311 | -1.8622 | -2.62486 | 0.0002 | 0.0112558 | yes |
| XLOC_013284 | XLOC_013284 | TMEFF2 | chr2:192814746-193059644 | NT | MOF | OK | 1.88775 | 0.516519 | -3.6547542 | -1.8698 | -1.69986 | 0.00925 | 0.166803 | no |
| XLOC_001786 | XLOC_001786 | HIST2H3D | chr1:149784779-149785236 | NT | MOF | OK | 11.1733 | 3.01345 | -3.70781 | -1.8906 | -1.74654 | 0.0053 | 0.117239 | no |
| XLOC_016582 | XLOC_016582 | NEIL3 | chr4:178230990-178284092 | NT | MOF | OK | 1.22913 | 0.330373 | -3.7204311 | -1.8955 | -1.85008 | 0.00475 | 0.10703 | no |
| XLOC_002502 | XLOC_002502 | SGPL1 | chr10:72575703-72640932 | NT | MOF | OK | 11.7764 | 3.14778 | -3.7411763 | -1.9035 | -3.71144 | 5.00E-05 | 0.00368902 | yes |
| XLOC_018679 | XLOC_018679 | HIST1H4D | chr6:26188937-26189304 | NT | MOF | OK | 37.1686 | 9.93445 | -3.7413848 | -1.9036 | -2.20285 | 0.0006 | 0.0251211 | yes |
| XLOC_014546 | XLOC_014546 | H1F0 | chr22:38201113-38203443 | NT | MOF | OK | 37.1547 | 9.91754 | -3.7463625 | -1.9055 | -3.89278 | 5.00E-05 | 0.00368902 | yes |
| XLOC_002583 | XLOC_002583 | KIF20B | chr10:91461366-91534700 | NT | MOF | OK | 3.32064 | 0.885986 | -3.7479599 | -1.9061 | -3.03567 | 5.00E-05 | 0.00368902 | yes |
| XLOC_004824 | XLOC_004824 | TROAP | chr12:49716970-49725514 | NT | MOF | OK | 2.25532 | 0.599861 | -3.7597377 | -1.9106 | -1.08111 | 0.03195 | 0.340313 | no |
| XLOC_023299 | XLOC_023299 | MBTPS2 | chrX:21857655-21903541 | NT | MOF | OK | 10.9281 | 2.87916 | -3.7955862 | -1.9243 | -3.57709 | 5.00E-05 | 0.00368902 | yes |
| XLOC_017021 | XLOC_017021 | MLF1IP | chr4:185570766-185655286 | NT | MOF | OK | 2.89285 | 0.755501 | -3.8290485 | -1.937 | -1.89283 | 0.00335 | 0.0844479 | no |
| XLOC_017674 | XLOC_017674 | CENPK | chr5:64813592-64858995 | NT | MOF | OK | 3.36361 | 0.877539 | -3.8330034 | -1.9385 | -2.17408 | 0.00045 | 0.0205472 | yes |
| XLOC_004314 | XLOC_004314 | CDCA5 | chr11:64844926-64851615 | NT | MOF | OK | 4.44994 | 1.15464 | -3.8539631 | -1.9463 | -2.57707 | 0.0001 | 0.00661202 | yes |
| XLOC_009314 | XLOC_009314 | BIRC5 | chr17:76210276-76221716 | NT | MOF | OK | 8.94235 | 2.31715 | -3.859202 | -1.9483 | -3.05089 | 5.00E-05 | 0.00368902 | yes |
| XLOC_018671 | XLOC_018671 | HIST1H1A | chr6:26017259-26018040 | NT | MOF | OK | 4.37775 | 1.13008 | -3.8738408 | -1.9538 | -1.88976 | 0.00885 | 0.16225 | no |
| XLOC_009571 | XLOC_009571 | SPAG5 | chr17:26904582-26926056 | NT | MOF | OK | 4.34945 | 1.10164 | -3.9481591 | -1.9812 | -2.94224 | 5.00E-05 | 0.00368902 | yes |
| XLOC_018104 | XLOC_018104 | HIST1H2BI | chr6:26273203-26273640 | NT | MOF | OK | 34.7084 | 8.77909 | -3.9535305 | -1.9831 | -2.51455 | 5.00E-05 | 0.00368902 | yes |
| XLOC_018704 | XLOC_018704 | HIST1H2AM | chr6:27860476-27860963 | NT | MOF | OK | 40.5351 | 10.2282 | -3.9630727 | -1.9866 | -2.71816 | 5.00E-05 | 0.00368902 | yes |
| XLOC_023996 | XLOC_023996 | TMSB15A | chrX:101768609-101771699 | NT | MOF | OK | 1.23829 | 0.311857 | -3.9706981 | -1.9894 | -1.16884 | 0.0801 | 0.552256 | no |
| XLOC_002593 | XLOC_002593 | KIF11 | chr10:94352824-94415152 | NT | MOF | OK | 5.76094 | 1.43991 | -4.0009028 | -2.0003 | -3.31859 | 5.00E-05 | 0.00368902 | yes |
| XLOC_023785 | XLOC_023785 | SCML2 | chrX:18257432-18372844 | NT | MOF | OK | 0.932938 | 0.231853 | -4.0238341 | -2.0086 | -2.05427 | 0.0015 | 0.0495902 | yes |
| XLOC_019096 | XLOC_019096 | MTFR2 | chr6:136552167-136571449 | NT | MOF | OK | 1.1626 | 0.28281 | -4.1108872 | -2.0395 | -1.70214 | 0.0083 | 0.155705 | no |
| XLOC_018130 | XLOC_018130 | HIST1H2AL | chr6:27833106-27833576 | NT | MOF | OK | 34.2085 | 8.24017 | -4.1514313 | -2.0536 | -2.61192 | 5.00E-05 | 0.00368902 | yes |
| XLOC_001444 | XLOC_001444 | CLSPN | chr1:36197712-36235551 | NT | MOF | OK | 1.4913 | 0.359103 | -4.1528475 | -2.0541 | -2.66932 | 0.00015 | 0.00902985 | yes |
| XLOC_004938 | XLOC_004938 | INHBE | chr12:57849095-57851791 | NT | MOF | OK | 0.926358 | 0.222707 | -4.159537 | -2.0564 | -1.72218 | 0.01095 | 0.184791 | no |
| XLOC_008705 | XLOC_008705 | GINS2 | chr16:85711279-85722588 | NT | MOF | OK | 2.79646 | 0.667608 | -4.1887754 | -2.0665 | -1.86751 | 0.0025 | 0.0711765 | no |
| XLOC_020385 | XLOC_020385 | SP4 | chr7:21467688-21554151 | NT | MOF | OK | 1.08832 | 0.258964 | -4.2025919 | -2.0713 | -2.35508 | 0.0005 | 0.0219203 | yes |
| XLOC_008792 | XLOC_008792 | FAM64A | chr17:6347734-6354385 | NT | MOF | OK | 3.46094 | 0.818094 | -4.2304919 | -2.0808 | -2.08119 | 0.00125 | 0.0434626 | yes |
| XLOC_020442 | XLOC_020442 | ANLN | chr7:36363758-36493400 | NT | MOF | OK | 16.0554 | 3.78511 | -4.2417261 | -2.0847 | -4.00246 | 5.00E-05 | 0.00368902 | yes |
| XLOC_021477 | XLOC_021477 | EFHA2 | chr8:16884746-16980148 | NT | MOF | OK | 8.94735 | 2.0753 | -4.3113526 | -2.1081 | -3.69825 | 5.00E-05 | 0.00368902 | yes |
| XLOC_019023 | XLOC_019023 | OSTM1 | chr6:108362612-108395941 | NT | MOF | OK | 26.546 | 6.06882 | -4.3741617 | -2.129 | -4.53108 | 5.00E-05 | 0.00368902 | yes |
| XLOC_016872 | XLOC_016872 | CENPE | chr4:104026962-104119566 | NT | MOF | OK | 2.67542 | 0.609385 | -4.3903608 | -2.1343 | -3.25464 | 5.00E-05 | 0.00368902 | yes |

| | | | | | | | | | | | | | | |
|---|---|---|---|---|---|---|---|---|---|---|---|---|---|---|
| XLOC_018125 | XLOC_018125 | HIST1H2AI | chr6:27775976-27776445 | NT | MOF | OK | 24.304 | 5.51448 | -4.4073059 | -2.1399 | -2.3092 | 0.0008 | 0.0310256 | yes |
| XLOC_007518 | XLOC_007518 | OIP5 | chr15:41601465-41624819 | NT | MOF | OK | 1.11512 | 0.250963 | -4.4433642 | -2.1517 | -1.62833 | 0.0162 | 0.230883 | no |
| XLOC_003637 | XLOC_003637 | CNIH2 | chr11:66045671-66051685 | NT | MOF | OK | 1.66692 | 0.373349 | -4.4647769 | -2.1586 | -1.78052 | 0.0036 | 0.088357 | no |
| XLOC_009022 | XLOC_009022 | LINC00672 | chr17:37081420-37085637 | NT | MOF | OK | 0.886905 | 0.197113 | -4.4994749 | -2.1698 | -1.9631 | 0.00515 | 0.114761 | no |
| XLOC_014577 | XLOC_014577 | MCHR1 | chr22:41075181-41078818 | NT | MOF | OK | 1.04153 | 0.230733 | -4.5140054 | -2.1744 | -1.59964 | 0.01955 | 0.260237 | no |
| XLOC_018703 | XLOC_018703 | HIST1H3J | chr6:27858092-27858570 | NT | MOF | OK | 12.2737 | 2.70986 | -4.5292746 | -2.1793 | -2.11655 | 0.00215 | 0.0645533 | no |
| XLOC_005690 | XLOC_005690 | C12orf76 | chr12:110478982-110505500 | NT | MOF | OK | 2.41356 | 0.528239 | -4.5690682 | -2.1919 | -2.35405 | 0.00015 | 0.00902985 | yes |
| XLOC_014559 | XLOC_014559 | APOBEC3A,APOE | chr22:39353526-39388784 | NT | MOF | OK | 2.76366 | 0.601684 | -4.5932084 | -2.1995 | -2.09501 | 0.0003 | 0.0149383 | yes |
| XLOC_006356 | XLOC_006356 | CDKN3 | chr14:54863672-54886934 | NT | MOF | OK | 4.67718 | 1.01142 | -4.6243697 | -2.2093 | -1.98701 | 0.00395 | 0.0940846 | no |
| XLOC_018682 | XLOC_018682 | HIST1H1D | chr6:26234439-26235216 | NT | MOF | OK | 19.9705 | 4.30795 | -4.6357316 | -2.2128 | -2.9046 | 5.00E-05 | 0.00368902 | yes |
| XLOC_016252 | XLOC_016252 | NCAPG | chr4:17812435-18023483 | NT | MOF | OK | 3.76218 | 0.791242 | -4.7547779 | -2.2494 | -2.25888 | 0.00075 | 0.0295603 | yes |
| XLOC_018090 | XLOC_018090 | HIST1H4A | chr6:26021906-26022278 | NT | MOF | OK | 18.0513 | 3.79611 | -4.7552099 | -2.2495 | -2.55653 | 0.0034 | 0.0848247 | no |
| XLOC_013662 | XLOC_013662 | MYBL2 | chr20:42295708-42345122 | NT | MOF | OK | 4.32045 | 0.905239 | -4.7727175 | -2.2548 | -2.958 | 5.00E-05 | 0.00368902 | yes |
| XLOC_014853 | XLOC_014853 | CENPM | chr22:42334740-42343148 | NT | MOF | OK | 2.48454 | 0.519966 | -4.778274 | -2.2565 | -1.64231 | 0.0036 | 0.088357 | no |
| XLOC_018696 | XLOC_018696 | HIST1H2BL | chr6:27775256-27775709 | NT | MOF | OK | 21.2221 | 4.42407 | -4.796963 | -2.2621 | -2.51489 | 0.00025 | 0.0132096 | yes |
| XLOC_007230 | XLOC_007230 | CCNB2 | chr15:59397283-59417244 | NT | MOF | OK | 5.68711 | 1.17077 | -4.8575809 | -2.2802 | -2.67926 | 0.0002 | 0.0112558 | yes |
| XLOC_016498 | XLOC_016498 | PLK4 | chr4:128802015-128820377 | NT | MOF | OK | 1.86145 | 0.382761 | -4.8632175 | -2.2819 | -2.6334 | 5.00E-05 | 0.00368902 | yes |
| XLOC_010805 | XLOC_010805 | WDR62 | chr19:36545782-36596012 | NT | MOF | OK | 1.35467 | 0.276233 | -4.9040846 | -2.294 | -2.60444 | 0.0003 | 0.0149383 | yes |
| XLOC_022265 | XLOC_022265 | SLC1A1 | chr9:4490426-4587469 | NT | MOF | OK | 11.5408 | 2.35141 | -4.9080339 | -2.2952 | -4.16373 | 5.00E-05 | 0.00368902 | yes |
| XLOC_016169 | XLOC_016169 | TACC3 | chr4:1723216-1746905 | NT | MOF | OK | 7.48225 | 1.50764 | -4.962889 | -2.3112 | -3.33831 | 5.00E-05 | 0.00368902 | yes |
| XLOC_004658 | XLOC_004658 | RAD51AP1 | chr12:4647949-4669213 | NT | MOF | OK | 1.83557 | 0.368918 | -4.9755501 | -2.3149 | -2.23674 | 0.0005 | 0.0219203 | yes |
| XLOC_001093 | XLOC_001093 | HIST3H2BB | chr1:228645807-228646259 | NT | MOF | OK | 3.06509 | 0.602251 | -5.0893896 | -2.3475 | -1.45318 | 0.0262 | 0.307488 | no |
| XLOC_014626 | XLOC_014626 | GTSE1 | chr22:46692637-46726707 | NT | MOF | OK | 3.25843 | 0.627119 | -5.1958719 | -2.3774 | -2.83444 | 0.0001 | 0.00661202 | yes |
| XLOC_006023 | XLOC_006023 | SKA3 | chr13:21727733-21753220 | NT | MOF | OK | 2.54892 | 0.484937 | -5.2561879 | -2.394 | -1.33082 | 0.07535 | 0.534115 | no |
| XLOC_001132 | XLOC_001132 | EXO1 | chr1:242011492-242053241 | NT | MOF | OK | 1.5094 | 0.2838 | -5.3185342 | -2.411 | -2.42821 | 0.0001 | 0.00661202 | yes |
| XLOC_010196 | XLOC_010196 | SKA1 | chr18:47901391-47920538 | NT | MOF | OK | 2.03262 | 0.381611 | -5.3264188 | -2.4132 | -2.57325 | 0.0003 | 0.0149383 | yes |
| XLOC_015061 | XLOC_015061 | KIF15 | chr3:44803208-44894748 | NT | MOF | OK | 1.03808 | 0.194299 | -5.3426935 | -2.4176 | -2.49248 | 0.0004 | 0.0189063 | yes |
| XLOC_011479 | XLOC_011479 | ASF1B | chr19:14230320-14247440 | NT | MOF | OK | 2.34657 | 0.423023 | -5.5471452 | -2.4717 | -2.18933 | 0.00315 | 0.0819677 | no |
| XLOC_023465 | XLOC_023465 | KIF4A | chrX:69509878-69640774 | NT | MOF | OK | 2.98817 | 0.535073 | -5.5846025 | -2.4815 | -3.28968 | 5.00E-05 | 0.00368902 | yes |
| XLOC_002088 | XLOC_002088 | ASPM | chr1:197053256-197115824 | NT | MOF | OK | 3.49898 | 0.625247 | -5.5961564 | -2.4844 | -4.0375 | 5.00E-05 | 0.00368902 | yes |
| XLOC_002349 | XLOC_002349 | MCM10 | chr10:13203553-13253104 | NT | MOF | OK | 0.762966 | 0.134975 | -5.6526468 | -2.4989 | -1.99747 | 0.00625 | 0.128833 | no |
| XLOC_007179 | XLOC_007179 | WDR76 | chr15:44119111-44160617 | NT | MOF | OK | 1.83934 | 0.324712 | -5.6645273 | -2.502 | -2.85388 | 5.00E-05 | 0.00368902 | yes |
| XLOC_008552 | XLOC_008552 | SHCBP1 | chr16:46614467-46655311 | NT | MOF | OK | 6.94391 | 1.21881 | -5.6972867 | -2.5103 | -3.64136 | 5.00E-05 | 0.00368902 | yes |
| XLOC_002598 | XLOC_002598 | CEP55 | chr10:95256368-95288849 | NT | MOF | OK | 5.9692 | 1.04225 | -5.7272248 | -2.5178 | -3.37327 | 5.00E-05 | 0.00368902 | yes |
| XLOC_018091 | XLOC_018091 | HIST1H3C | chr6:26045638-26046097 | NT | MOF | OK | 38.1818 | 6.65386 | -5.7382933 | -2.5206 | -3.07039 | 5.00E-05 | 0.00368902 | yes |
| XLOC_018103 | XLOC_018103 | HIST1H2BH | chr6:26251878-26252303 | NT | MOF | OK | 31.0444 | 5.35812 | -5.7938979 | -2.5345 | -2.92563 | 5.00E-05 | 0.00368902 | yes |
| XLOC_018700 | XLOC_018700 | HIST1H1B | chr6:27834569-27835359 | NT | MOF | OK | 53.2578 | 9.1802 | -5.8013769 | -2.5364 | -4.00076 | 5.00E-05 | 0.00368902 | yes |
| XLOC_018131 | XLOC_018131 | HIST1H2BO | chr6:27861202-27861669 | NT | MOF | OK | 32.8823 | 5.66781 | -5.8015883 | -2.5365 | -3.04102 | 5.00E-05 | 0.00368902 | yes |
| XLOC_012045 | XLOC_012045 | RRM2 | chr2:10262694-10271546 | NT | MOF | OK | 13.122 | 2.20646 | -5.9470827 | -2.5722 | -4.14384 | 5.00E-05 | 0.00368902 | yes |
| XLOC_002605 | XLOC_002605 | HELLS | chr10:96305573-96361856 | NT | MOF | OK | 2.85284 | 0.473859 | -6.0204407 | -2.5899 | -3.04645 | 5.00E-05 | 0.00368902 | yes |
| XLOC_018702 | XLOC_018702 | HIST1H4L | chr6:27840925-27841289 | NT | MOF | OK | 28.3031 | 4.67148 | -6.0587009 | -2.599 | -2.57847 | 0.0003 | 0.0149383 | yes |
| XLOC_021527 | XLOC_021527 | ESCO2 | chr8:27632057-27662424 | NT | MOF | OK | 1.04509 | 0.171878 | -6.0804175 | -2.6042 | -2.48544 | 0.00075 | 0.0295603 | yes |

| | | | | | | | | | | | | | | |
|---|---|---|---|---|---|---|---|---|---|---|---|---|---|---|
| XLOC_015603 | XLOC_015603 | SGOL1 | chr3:20202084-20227919 | NT | MOF | OK | 1.06764 | 0.17526 | -6.0917494 | -2.6069 | -1.42196 | 5.00E-05 | 0.00368902 | yes |
| XLOC_001548 | XLOC_001548 | ORC1 | chr1:52838500-52870143 | NT | MOF | OK | 0.775667 | 0.126852 | -6.11474 | -2.6123 | -2.09042 | 0.0022 | 0.0657284 | no |
| XLOC_013687 | XLOC_013687 | UBE2C | chr20:44441254-44445596 | NT | MOF | OK | 15.1673 | 2.47702 | -6.1232045 | -2.6143 | -2.83522 | 5.00E-05 | 0.00368902 | yes |
| XLOC_018382 | XLOC_018382 | IRAK1BP1 | chr6:79577188-79608320 | NT | MOF | OK | 1.43649 | 0.234106 | -6.1360666 | -2.6173 | -1.83969 | 0.0135 | 0.207297 | no |
| XLOC_008372 | XLOC_008372 | PKMYT1 | chr16:3019341-3030540 | NT | MOF | OK | 2.0765 | 0.338281 | -6.1383879 | -2.6179 | -1.59897 | 0.0122 | 0.195523 | no |
| XLOC_001601 | XLOC_001601 | DEPDC1 | chr1:68939834-68962799 | NT | MOF | OK | 2.05908 | 0.334152 | -6.1621059 | -2.6234 | -3.2021 | 5.00E-05 | 0.00368902 | yes |
| XLOC_023936 | XLOC_023936 | ERCC6L | chrX:71401525-71483814 | NT | MOF | OK | 1.03013 | 0.166067 | -6.2030987 | -2.633 | -1.25444 | 0.08105 | 0.554412 | no |
| XLOC_006820 | XLOC_006820 | DLGAP5 | chr14:55614833-55658396 | NT | MOF | OK | 3.96913 | 0.637827 | -6.2228943 | -2.6376 | -3.25821 | 5.00E-05 | 0.00368902 | yes |
| XLOC_018127 | XLOC_018127 | HIST1H2BM | chr6:27782821-27783267 | NT | MOF | OK | 21.9995 | 3.49952 | -6.2864336 | -2.6522 | -2.69815 | 5.00E-05 | 0.00368902 | yes |
| XLOC_017291 | XLOC_017291 | LMNB1 | chr5:126112314-126172712 | NT | MOF | OK | 4.65396 | 0.738627 | -6.3008257 | -2.6555 | -3.09706 | 5.00E-05 | 0.00368902 | yes |
| XLOC_012310 | XLOC_012310 | NCAPH | chr2:97001483-97041274 | NT | MOF | OK | 1.12916 | 0.176242 | -6.4068724 | -2.6796 | -2.69855 | 5.00E-05 | 0.00368902 | yes |
| XLOC_022956 | XLOC_022956 | ZNF367 | chr9:99148224-99180669 | NT | MOF | OK | 2.13047 | 0.332304 | -6.4112078 | -2.6806 | -3.0558 | 0.0001 | 0.00661202 | yes |
| XLOC_013101 | XLOC_013101 | BUB1 | chr2:111395408-111435684 | NT | MOF | OK | 3.30617 | 0.514733 | -6.4230776 | -2.6833 | -3.27819 | 5.00E-05 | 0.00368902 | yes |
| XLOC_009476 | XLOC_009476 | AURKB | chr17:8108048-8113944 | NT | MOF | OK | 4.72325 | 0.732681 | -6.4465299 | -2.6885 | -2.68326 | 5.00E-05 | 0.00368902 | yes |
| XLOC_007145 | XLOC_007145 | CASC5 | chr15:40886446-40954881 | NT | MOF | OK | 1.85868 | 0.283099 | -6.5654771 | -2.7149 | -3.29985 | 5.00E-05 | 0.00368902 | yes |
| XLOC_007408 | XLOC_007408 | LOC100507118 | chr15:91509267-91537881 | NT | MOF | OK | 0.894528 | 0.136096 | -6.5727722 | -2.7165 | -0.80007 | 0.48905 | 0.989872 | no |
| XLOC_010092 | XLOC_010092 | NDC80 | chr18:2571509-2616634 | NT | MOF | OK | 3.1141 | 0.466115 | -6.6809693 | -2.7401 | -2.83267 | 0.00015 | 0.00902985 | yes |
| XLOC_017459 | XLOC_017459 | HMMR | chr5:162887516-162918953 | NT | MOF | OK | 3.04886 | 0.455573 | -6.6923632 | -2.7425 | -2.89862 | 5.00E-05 | 0.00368902 | yes |
| XLOC_017338 | XLOC_017338 | KIF20A | chr5:137514416-137549032 | NT | MOF | OK | 5.19653 | 0.767889 | -6.7672932 | -2.7586 | -2.02503 | 0.0062 | 0.128239 | no |
| XLOC_001911 | XLOC_001911 | IQGAP3 | chr1:156495196-156542396 | NT | MOF | OK | 3.24324 | 0.47821 | -6.7820414 | -2.7617 | -3.72793 | 5.00E-05 | 0.00368902 | yes |
| XLOC_018685 | XLOC_018685 | HIST1H3G | chr6:26271145-26271612 | NT | MOF | OK | 26.1059 | 3.80543 | -6.8601709 | -2.7783 | -3.13813 | 5.00E-05 | 0.00368902 | yes |
| XLOC_013427 | XLOC_013427 | HJURP | chr2:234745485-234763212 | NT | MOF | OK | 3.58554 | 0.515661 | -6.9532891 | -2.7977 | -3.34279 | 0.0001 | 0.00661202 | yes |
| XLOC_005531 | XLOC_005531 | PRIM1 | chr12:57125363-57146146 | NT | MOF | OK | 1.36825 | 0.196677 | -6.9568379 | -2.7984 | -2.03747 | 0.00535 | 0.1177 | no |
| XLOC_001049 | XLOC_001049 | CENPF | chr1:214776531-214837914 | NT | MOF | OK | 4.11137 | 0.571513 | -7.1938346 | -2.8468 | -4.57677 | 5.00E-05 | 0.00368902 | yes |
| XLOC_000847 | XLOC_000847 | NUF2 | chr1:163291722-163325553 | NT | MOF | OK | 2.75651 | 0.378839 | -7.2762044 | -2.8632 | -2.69971 | 0.0003 | 0.0149383 | yes |
| XLOC_015894 | XLOC_015894 | POLQ | chr3:121150272-121264853 | NT | MOF | OK | 0.732354 | 0.0981098 | -7.4646366 | -2.9001 | -3.01974 | 5.00E-05 | 0.00368902 | yes |
| XLOC_018673 | XLOC_018673 | HIST1H3B | chr6:26031816-26032288 | NT | MOF | OK | 89.9065 | 11.8771 | -7.569735 | -2.9202 | -4.05773 | 5.00E-05 | 0.00368902 | yes |
| XLOC_009671 | XLOC_009671 | TOP2A | chr17:38544772-38574202 | NT | MOF | OK | 15.1139 | 1.98553 | -7.612023 | -2.9283 | -5.16807 | 5.00E-05 | 0.00368902 | yes |
| XLOC_002469 | XLOC_002469 | CDK1 | chr10:62538088-62554610 | NT | MOF | OK | 4.708 | 0.617488 | -7.6244397 | -2.9306 | -2.99714 | 5.00E-05 | 0.00368902 | yes |
| XLOC_000291 | XLOC_000291 | CDCA8 | chr1:38158072-38175391 | NT | MOF | OK | 4.07832 | 0.52484 | -7.7705968 | -2.958 | -3.07548 | 0.0001 | 0.00661202 | yes |
| XLOC_016064 | XLOC_016064 | NCEH1 | chr3:172348434-172429008 | NT | MOF | OK | 34.4941 | 4.38761 | -7.861706 | -2.9748 | -6.22048 | 5.00E-05 | 0.00368902 | yes |
| XLOC_003502 | XLOC_003502 | FAM111B | chr11:58874657-58894888 | NT | MOF | OK | 1.44326 | 0.183462 | -7.8668062 | -2.9758 | -2.50468 | 0.0515 | 0.444472 | no |
| XLOC_000334 | XLOC_000334 | CDC20 | chr1:43824625-43828873 | NT | MOF | OK | 10.0184 | 1.26693 | -7.9076192 | -2.9832 | -3.85898 | 5.00E-05 | 0.00368902 | yes |
| XLOC_018675 | XLOC_018675 | HIST1H2BB | chr6:26043454-26043885 | NT | MOF | OK | 11.9973 | 1.51575 | -7.9150915 | -2.9846 | -2.47131 | 0.0023 | 0.0677129 | no |
| XLOC_000369 | XLOC_000369 | RAD54L | chr1:46713366-46769038 | NT | MOF | OK | 0.814761 | 0.10259 | -7.9419144 | -2.9895 | -0.434303 | 0.2271 | 0.830507 | no |
| XLOC_021958 | XLOC_021958 | STAR | chr8:38000217-38008600 | NT | MOF | OK | 1.03087 | 0.129252 | -7.97566 | -2.9956 | -2.30861 | 0.0025 | 0.0711765 | no |
| XLOC_007137 | XLOC_007137 | BUB1B,PAK6 | chr15:40453209-40569688 | NT | MOF | OK | 2.26632 | 0.283059 | -8.0065287 | -3.0012 | -3.09266 | 5.00E-05 | 0.00368902 | yes |
| XLOC_003189 | XLOC_003189 | MKI67 | chr10:129894924-129924468 | NT | MOF | OK | 5.29927 | 0.642871 | -8.2431312 | -3.0432 | -5.10791 | 5.00E-05 | 0.00368902 | yes |
| XLOC_013108 | XLOC_013108 | CKAP2L | chr2:113495443-113522254 | NT | MOF | OK | 2.92476 | 0.342572 | -8.5376505 | -3.0938 | -3.40181 | 5.00E-05 | 0.00368902 | yes |
| XLOC_024214 | XLOC_024214 | RAB39B | chrX:154487525-154493852 | NT | MOF | OK | 0.823187 | 0.0910444 | -9.0415995 | -3.1766 | -2.21861 | 0.00525 | 0.116346 | no |
| XLOC_014406 | XLOC_014406 | CDC45 | chr22:19467413-19508135 | NT | MOF | OK | 1.92999 | 0.21184 | -9.1106023 | -3.1875 | -2.35636 | 0.00035 | 0.017008 | yes |
| XLOC_007996 | XLOC_007996 | PLK1 | chr16:23690200-23724821 | NT | MOF | OK | 7.53254 | 0.770301 | -9.7786969 | -3.2896 | -4.01033 | 5.00E-05 | 0.00368902 | yes |

| | | | | | | | | | | | | | | |
|---|---|---|---|---|---|---|---|---|---|---|---|---|---|---|
| XLOC_002097 | XLOC_002097 | KIF14 | chr1:200520624-200589862 | NT | MOF | OK | 1.7658 | 0.179903 | -9.8152894 | -3.295 | -3.96142 | 5.00E-05 | 0.00368902 | yes |
| XLOC_021928 | XLOC_021928 | PBK | chr8:27667137-27695349 | NT | MOF | OK | 4.70272 | 0.478264 | -9.8328956 | -3.2976 | -3.49626 | 5.00E-05 | 0.00368902 | yes |
| XLOC_005248 | XLOC_005248 | CDCA3 | chr12:6957971-6960456 | NT | MOF | OK | 4.5444 | 0.448889 | -10.123661 | -3.3397 | -2.93862 | 0.0005 | 0.0219203 | yes |
| XLOC_007156 | XLOC_007156 | NUSAP1 | chr15:41624891-41673248 | NT | MOF | OK | 4.84938 | 0.471974 | -10.274676 | -3.361 | -3.84625 | 5.00E-05 | 0.00368902 | yes |
| XLOC_002170 | XLOC_002170 | NEK2 | chr1:211831598-211848972 | NT | MOF | OK | 2.21647 | 0.211263 | -10.49152 | -3.3912 | -2.6557 | 0.00025 | 0.0132096 | yes |
| XLOC_017805 | XLOC_017805 | C5orf15 | chr5:133291197-133304406 | NT | MOF | OK | 49.8543 | 4.4516 | -11.199187 | -3.4853 | -6.54428 | 5.00E-05 | 0.00368902 | yes |
| XLOC_001035 | XLOC_001035 | DTL | chr1:212113740-212278187 | NT | MOF | OK | 2.3943 | 0.2119 | -11.299198 | -3.4982 | -1.86335 | 0.1632 | 0.746037 | no |
| XLOC_018684 | XLOC_018684 | HIST1H3F | chr6:26250369-26250835 | NT | MOF | OK | 29.048 | 2.51853 | -11.533712 | -3.5278 | -3.58433 | 5.00E-05 | 0.00368902 | yes |
| XLOC_013219 | XLOC_013219 | SPC25 | chr2:169727400-169746944 | NT | MOF | OK | 2.03738 | 0.174232 | -11.693489 | -3.5476 | -2.43617 | 0.0097 | 0.171093 | no |
| XLOC_009782 | XLOC_009782 | KIF18B | chr17:43002078-43025079 | NT | MOF | OK | 2.66212 | 0.225385 | -11.811434 | -3.5621 | -3.42771 | 5.00E-05 | 0.00368902 | yes |
| XLOC_017666 | XLOC_017666 | DEPDC1B | chr5:59892738-59995993 | NT | MOF | OK | 1.38564 | 0.116974 | -11.845709 | -3.5663 | -2.57411 | 0.00045 | 0.0205472 | yes |
| XLOC_000350 | XLOC_000350 | KIF2C | chr1:45205489-45233438 | NT | MOF | OK | 3.78516 | 0.282751 | -13.386902 | -3.7428 | -3.67212 | 5.00E-05 | 0.00368902 | yes |
| XLOC_012086 | XLOC_012086 | CENPA | chr2:27008881-27017455 | NT | MOF | OK | 2.22204 | 0.156147 | -14.230437 | -3.8309 | -2.68152 | 0.00185 | 0.0581429 | no |
| XLOC_018697 | XLOC_018697 | HIST1H2AJ | chr6:27782079-27782518 | NT | MOF | OK | 10.8303 | 0.71513 | -15.144519 | -3.9207 | -3.10713 | 0.0111 | 0.185768 | no |
| XLOC_018386 | XLOC_018386 | TTK | chr6:80714321-80752244 | NT | MOF | OK | 3.29726 | 0.212398 | -15.523969 | -3.9564 | -3.56393 | 5.00E-05 | 0.00368902 | yes |
